# Supplementary material for: Cluster randomised controlled trial of screening for atrial fibrillation in people aged 70 years and over to reduce stroke: protocol for the pilot study for the SAFER trial
Source: BMJ Open. 2022 Sep 9;12(9):e065066. doi: 10.1136/bmjopen-2022-065066 (PMC9472173; doi:10.1136/bmjopen-2022-065066)
Supplement: Supplementary data [file bmjopen-2022-065066supp001.pdf]

PRACTICE HEADED PAPER
PRACTICE ADDRESS
PRACTICE PARTNERS

Participant ID: <<Trial_ID>>

<<Title>> <<First name>> <<Surname>>
<<Address 1>>
<<Address 2>>
<<Address 3>>
<<Address 4>>
<<Address 5>>

<<Date>>

Dear <<Title>> <<First name>> <<Surname>>

Invitation to join a research trial:

The SAFER Trial

We are writing to let you know about a research trial that we are involved with. We are working with researchers from the University of Cambridge.

The trial aims to test whether screening at home for a common heart rate condition, Atrial Fibrillation (AF) would prevent people having strokes and heart attacks.

We are inviting you because you are in the age range where atrial fibrillation is more common. You have been selected at random from patients at your practice in this age range. You will not need to attend an appointment at the practice. You may still be able to take part if you already have atrial fibrillation.

You do not have to participate. Your decision will not affect any future health care you receive.

Enclosed with this letter is:

1. **Participant Information Sheet**
2. **Consent Form (including contact details) (complete and return to take part)**
3. **'I do not wish to take part' Reply Slip (ONLY complete and return if you DO NOT want to take part)**
4. **Freepost envelope**

Please read the **Participant Information Sheet,** the **Consent Form** and **Reply Slip** carefully. Please take your time to consider all the information. You can talk to others about your decision to take part, and contact the researchers to ask them any questions you have, or talk to your GP.

Once you have made your decision, please see instructions at the bottom of page 2 for what you need to do next. (**Note:** If the research team does not hear from you we may send you a **reminder letter**.)

PRACTICE HEADED PAPER
PRACTICE ADDRESS
PRACTICE PARTNERS

If you would like more information

If you have any questions please contact the SAFER Trial team by **phone** on **01223 763491** (working hours are Monday to Friday 9am-5pm, there is an answer phone on this number if they miss your call), or you can contact them by **email**, safer@medschl.cam.ac.uk. There is also more information about the trial on the website - https://www.safer.phpc.cam.ac.uk/.

Thank you for taking the time to read this letter.

Yours sincerely,

<Signature>

<Name of GP>

What to do next:

✓ I WOULD like to take part

If you would like to take part please complete the trial consent form. You can do this either:

Online: by following this secure link bit.ly/saferconsent and entering the code below to fill in your consent form online:

Code: <<Token>>

OR;

By post: by completing the enclosed **Consent Form**, checking that the contact details on the second page of the form are accurate. Please amend and add any if applicable. Please return the completed **Consent Form** to the research team in the Freepost envelope provided (no stamp required).

X I DO NOT want to take part

If you do not wish to take part, we would appreciate it if you would complete the '**I do not wish to take part**' Reply Slip enclosed and return it to the research team in the Freepost envelope provided (no stamp required). We would appreciate it if you would let the researchers know the reasons why you do not wish to participate in the SAFER trial.

Williams K, *et al. BMJ Open* 2022; 12:e065066. doi: 10.1136/bmjopen-2022-065066
