# Supplementary material for: Cluster randomised controlled trial of screening for atrial fibrillation in people aged 70 years and over to reduce stroke: protocol for the pilot study for the SAFER trial
Source: BMJ Open. 2022 Sep 9;12(9):e065066. doi: 10.1136/bmjopen-2022-065066 (PMC9472173; doi:10.1136/bmjopen-2022-065066)
Supplement: Supplementary data [file bmjopen-2022-065066supp002.pdf]

UNIVERSITY OF CAMBRIDGE
Public Health and Primary Care
The Primary Care Unit

SAFER trial

# The SAFER Trial –

## Screening for Atrial Fibrillation with ECG to Reduce stroke

# Participant Information Sheet

*V1.3 03-02-2021_remote*

## We invite you to take part in a research trial about screening for atrial fibrillation to reduce stroke

- Before you decide whether to take part, it is important for you to understand why the research is being done and what it will involve.
- Please take your time to read the following information carefully. You can discuss it with your friends and relatives if you wish.
- You are free to decide whether or not to take part in this trial. If you choose not to take part, it will not affect the care that you receive from your doctors.
- Please don't hesitate to ask us any questions you may have, if anything is unclear, or if you would like more information.

FUNDED BY
NIHR | National Institute for Health Research

This project is funded by the National Institute for Health Research (NIHR) [Programme Grants for Applied Research (grant reference number RP-PG-0217-20007)]. The views expressed are those of the author(s) and not necessarily those of the NIHR or the Department of Health and Social Care.

# Part 1: Trial Summary

## A brief summary of the trial

- Atrial fibrillation (AF) is a common irregular heartbeat, and is a major risk factor for stroke unless treated with medication.
- We (researchers at the University of Cambridge and the University of Oxford) want to find out whether a national screening programme for atrial fibrillation (i) is possible/achievable, (ii) will prevent strokes and (iii) is good value for money for the NHS.
- We are inviting patients aged 70 years and over to take part in a research trial that aims to answer these questions. If you have atrial fibrillation you may still be able to take part.
- If you agree to take part, we will ask for your consent to give us access to relevant information from your medical and other health-related records. This will be information related to stroke and associated conditions (you can read more about this later in this information sheet). We will make sure that this information is looked after securely, remains confidential and is only used for the purposes described – all in accordance with the General Data Protection Regulations.
- Your practice may or may not offer screening for atrial fibrillation as part of the trial. If your practice does offer atrial fibrillation screening we will send you further information about this and you can choose then whether you would like to be screened or not.
- You can also visit the trial website at https://www.safer.phpc.cam.ac.uk
- If you have any questions you can phone us on **01223 763491** or email at safer@medschl.cam.ac.uk

## Contents

Part 1
Trial Summary........*Page 2*

Part 2
Further Trial Details........*Page 3*

Part 3
Data Confidentiality........*Page 8*

Part 4
Contact Us........*Page 11*

## How to contact us

If you have any questions you can call us on
**01223 763491**
(Monday to Friday 9am-5pm)

Or email us at
safer@medschl.cam.ac.uk

You can also visit the trial website at
www.safer.phpc.cam.ac.uk

# *Part 2: Further Trial Details*

## Why are we doing this trial?

Atrial fibrillation (AF) is a heart condition that causes an irregular heartbeat. It affects over 1 in 10 people over the age of 70 but does not necessarily cause symptoms.

Having atrial fibrillation increases the risk of having a stroke 5-fold, but treatment with medication can significantly lower this risk as well as lowering your risk of having a heart attack.

We want to find out whether screening people over the age of 70 years for atrial fibrillation and treating them with medication:

1. is possible/achievable in GP practices (**no GP practice visit needed**);
2. prevents the number of strokes we think it will;
3. is good value for money for the NHS.

The results will help the NHS decide whether to start a national screening programme for atrial fibrillation to reduce the number of strokes.

## Why have I been invited?

You have been invited because you are in the age range where atrial fibrillation is more common. You have been selected, at random, from patients at your practice in this age range.

## I have a heart condition. Can I take part?

Yes. If you have the following you can still take part:

- Atrial fibrillation and are **not** taking blood thinning (anticoagulation) medication like warfarin
- A pacemaker
- If you are taking aspirin or clopidogrel

## Do I have to take part?

No. You do not have to take part. If you decide not to take part, or change your mind at any stage, your care at your GP practice will not be affected.



Williams K, *et al. BMJ Open* 2022; 12:e065066. doi: 10.1136/bmjopen-2022-065066

# What will I have to do?

## Consent

We will ask you to confirm your consent to take part. You can do this online by following the instructions in the covering letter. Alternatively, you can sign the enclosed paper consent form and post it back to us in the Freepost envelope provided.

We are asking you to give permission for specific information from your medical records to be shared with the research team at various points over the next few years. This information will be collected from your GP practice records and from other health-related records. The kind of information we will collect will include the medications you take, your use of health care services and stroke and cardiovascular disease related factors (for example weight, age, blood pressure, other medical conditions, and how well your kidneys and liver work). You will not need to do anything to provide this information. We will collect this information from everyone who consents to be part of the trial, whether they are screened for atrial fibrillation or not. Collecting this information will help us to understand whether screening for atrial fibrillation makes a difference, by comparing information about people who do and do not undergo this screening.

# What else might I be invited to do?

## Possibly take part in screening for atrial fibrillation

You may be invited to be screened for atrial fibrillation. This would be done at home. You would be sent the ECG recording device and instructions. There would be no need to attend an appointment at your GP practice or for anyone to visit you at home. Screening involves simply holding a small, safe, non-invasive recording device in your hands for 30 seconds at a time.

**We will send you more information about what is involved and the potential benefits and harms of screening at the time if you are invited**.

You are under no obligation to take part.

Not all the practices that are taking part in the trial will offer atrial fibrillation screening. Whether your practice does or not is determined by chance, so it may be that you are not invited to have screening.

### Possibly complete questionnaires

On up to 3 separate occasions we may send you a questionnaire to complete and return in a Freepost envelope (or complete online). You are under no obligation to complete this questionnaire.

### Possibly be invited to take part in other studies

Some people may be invited to take part in optional interviews and/or other studies related to atrial fibrillation and/or screening. If this is the case, you will be given further information about what these would involve. You will be free to decide at that point whether or not you want to do them.

## Are there any benefits of taking part?

There may not be any direct benefit to you of taking part. However, you may find it rewarding to know that you are contributing to research that aims to prevent stroke and heart attacks in the future.

## Are there any risks involved in taking part?

Identifiable medical data from your practice will be shared with the research team. All our data collection, storage and handling processes will comply with the relevant security policies and regulations. Every effort will be made to ensure the security and confidentiality of your data. Your usual medical care will not be affected by your participation in the trial.

## What if I change my mind about giving access to medical and health-related records?

If you change your mind about taking part you can withdraw your consent at any time. If this is the case, please telephone us or email us using the contact details in part 4 of this information sheet. Any of your information that has been collected up to that point will be kept and used for the purposes described in this information sheet. We will not collect any further health-related information about you and your medical care at your GP practice will not be affected.



Williams K, *et al. BMJ Open* 2022; 12:e065066. doi: 10.1136/bmjopen-2022-065066

## Who is organising and funding the trial?

The trial is being organised by the University of Cambridge working with the University of Oxford. The University of Cambridge and Cambridgeshire and Peterborough Clinical Commissioning Group (CCG) are the co-sponsors for the trial. The sponsors have overall responsibility for the conduct of the trial. It is funded by the National Institute for Health Research.

## Who has reviewed / approved the trial?

To protect your interests, all research in the NHS is looked at by an independent group of people, called a Research Ethics Committee. This trial has been reviewed and given favourable opinion by the London-Central NHS Research Ethics Committee. The science has been reviewed by experts in atrial fibrillation, stroke and screening in the NHS.

## How have patients and the public been involved in the trial?

Patient representatives and members of the public have been involved with the design of the trial and/or this information sheet. Patients are represented on a number of the trial research committees, and one is a co-investigator on the research grant.

## What will happen if something goes wrong?

If you have any concerns about any aspect of this trial, you should ask to speak to us (the research team) and we will do our best to answer your questions:

Telephone: 01223 763491

Email: safer@medschl.cam.ac.uk

Post: The SAFER Trial, University of Cambridge, Primary Care Unit, Strangeways Research Laboratory, 2 Worts Causeway, Cambridge, CB1 8RN

If you remain unhappy and wish to complain formally, please first contact Cambridgeshire and Peterborough Clinical Commissioning Group patient experience team.

FREEPHONE: 0800 279 2535 or 01223 725 588

Email: CAPCCG.pet@nhs.net

Post: Patient Experience Team, Lockton House, Clarendon Road, Cambridge, CB2 8FH



Williams K, *et al. BMJ Open* 2022; 12:e065066. doi: 10.1136/bmjopen-2022-065066

*Please note that due to the Covid-19 pandemic the patient experience team may not currently be able to respond to your query in a timely manner. Due to staff working remotely, contact by phone or email in the first instance is advised.*

If you are unhappy with a primary care service, such as your GP practice or pharmacist, you can complain either directly to the Practice Manager of the practice or if you prefer to NHS England, the organisation which manages complaints for these services:

Telephone: 0300 311 22 33 (Monday to Friday 8am to 6pm, excluding bank holidays)

Email: england.contactus@nhs.net

Write to: NHS England, PO Box 16738, Redditch B97 9PT

In the event that something does go wrong and you are harmed during the research and this is due to someone's negligence then you may have grounds for legal action for compensation against the University of Cambridge or the NHS or an individual through their professional indemnity (if appropriate) but you may have to pay your legal costs. The normal National Health Service complaints mechanisms will still be available to you (if appropriate). The University of Cambridge has arranged insurance in case something goes wrong and you are harmed but it is not due to anybody's fault (no-fault compensation).

## How will I find out the results of the trial?

At the end of this trial the results will be available to read on our trial website – please see our contact details in part 4 for the link. If you would like us to send you a copy of the results please get in touch with us.

# *Part 3: Data Confidentiality*

## How will information about me be kept confidential?

Your personal details will be collected from your GP practice including your name, address, contact details, date of birth, NHS number – "personal data". We will also collect information about you during your participation in the trial, some relating to your health from various sources – "trial data".

Your trial data will not include your personal data, and will be stored separately using a unique trial identification number.

All information about you (including your personal data) will be stored securely with access restricted to authorised members of the research team from the University of Cambridge and the University of Oxford. Only these people will access your personal data as they need to manage your participation in the trial, collect information from your medical and health records, or audit the data collection process. In addition, authorised staff who work for or with the sponsors of the trial or relevant regulatory authorities may require access to your personal data, your trial data and/or your medical records. This would be to check the accuracy of the trial data and ensure that it is being conducted in accordance with the relevant regulations. All information will be treated in the strictest confidence during that review process.

We will inform your GP that you are taking part in the trial.

## Information from medical and health records

The research team will collect information from your GP practice medical record. Usually your practice will pass this information securely to the research team. Sometimes your GP practice will allow the research team to access your medical records directly, or use a contracted third party. This is only done with the appropriate security checks and confidentiality agreements in place.

The research team will also collect information from other centrally held health-related records. The records we will use are Hospital Episodes Statistics data, civil registration mortality data (both held by the appropriate governing body, currently NHS Digital), the Sentinel Stroke National Audit Programme (SSNAP) database, and the Myocardial Ischaemia National Audit Project (MINAP)

database. To link this information the research team need to send identifying information such as your name, address, NHS number and date of birth to NHS Digital, SSNAP and MINAP so they can identify your health records correctly. It is possible that in the future we may need to link to another health record or registry that we consider to be relevant to the purposes of the research. We will use central NHS records to provide us with your current GP practice and your address, so that we can continue to collect follow-up information should you move. We will continue to collect health record data in the event of your death during the trial and follow-up period.

Only information relevant to the purpose of the trial or understanding how to screen for atrial fibrillation will be collected. We might use this information to decide whether to invite you to take part in other related research studies.

## General Data Protection Regulation

The University of Cambridge and NHS Cambridgeshire and Peterborough CCG are the co-sponsors for this trial based in the UK. The University of Cambridge will be using information from you and your medical records in order to undertake this trial and will act as the data controller for this trial. This means that we are responsible for looking after your information and using it properly. The SAFER Trial is part of an important long-term programme of research that relies on long-term follow-up of participants. We will retain your personal and trial data indefinitely to meet the purposes of medical research and any legal, accounting or reporting requirements.

Your rights to access, change or move the information that we hold are limited, as we need to manage it in specific ways in order for the research to be reliable and accurate. To safeguard your rights, we will use the minimum personally identifiable information possible.

You can find out more about how we use your information here https://www.medschl.cam.ac.uk/research/privacy-notice-how-we-use-your-research-data/

We will share your information with collaborating research organisations working with us (both internal and external to this trial) and commercial partners. Only trial data that cannot identify you will be shared. This will be governed by appropriate agreements. Some organisations may be outside of the European Economic Area, where data security regulations may be less stringent than those in the UK. The transfer of information will be done securely and in accordance with local security policies and the Data Protection Act 2018.



Williams K, *et al. BMJ Open* 2022; 12:e065066. doi: 10.1136/bmjopen-2022-065066

During the trial we will not be able to monitor your ability to consent to continued participation. We will keep any data already collected about you and will continue to collect data from your medical and other health records, in accordance with the consent that you granted at the start of the trial. If, however, we find out as a result of arranging your screening or through your involvement in optional interviews that you are unwell such that you are no longer able to consent to continued participation, we will withdraw you from the trial, while keeping any data about you that we have already collected.

Future updates relating to data confidentiality will be posted on the trial website https://www.safer.phpc.cam.ac.uk/. It is recommended that you visit the website regularly to keep up to date with the progress of the trial and data confidentiality information.



Williams K, *et al. BMJ Open* 2022; 12:e065066. doi: 10.1136/bmjopen-2022-065066

# *Part 4: Contact Us*

## Who do I contact if I have any questions?

Please get in touch with us - our details are below. You can also visit the trial website: https://www.safer.phpc.cam.ac.uk/ for more information.

The trial is registered with the ISRCTN https://www.isrctn.com/ISRCTN72104369.

### Contact details:

If you or someone on your behalf needs to contact the research team you can do so as follows:

**Phone:** contact us using the following number during working hours (Monday to Friday 9am – 5pm): **01223 763491**. If we miss your call or if you call outside these hours, there is an answer phone on this number. If you leave a message we will respond to you at the earliest opportunity.

**Email:** safer@medschl.cam.ac.uk

**Address:**
The SAFER Trial
University of Cambridge
Primary Care Unit
Strangeways Research Laboratory
2 Worts Causeway
Cambridge
CB1 8RN

**Website:** https://www.safer.phpc.cam.ac.uk/

SAFER
trial



Williams K, *et al. BMJ Open* 2022; 12:e065066. doi: 10.1136/bmjopen-2022-065066
