# Supplementary material for: Cluster randomised controlled trial of screening for atrial fibrillation in people aged 70 years and over to reduce stroke: protocol for the pilot study for the SAFER trial
Source: BMJ Open. 2022 Sep 9;12(9):e065066. doi: 10.1136/bmjopen-2022-065066 (PMC9472173; doi:10.1136/bmjopen-2022-065066)
Supplement: Supplementary data [file bmjopen-2022-065066supp003.pdf]

*[TO BE PRINTED ON TRIAL HEADED PAPER]*

**Participant ID:** <<participant ID>> / barcode

## SAFER Trial

# Consent Form

Version 1.1 11-12-2020

**Please complete and return this form only if you wish to join the SAFER Trial**

**Title:** The SAFER Trial – Screening for Atrial Fibrillation with ECG to Reduce stroke

**Chief Investigator:** Professor Jonathan Mant, University of Cambridge

**IRAS project ID:** 272184

**Ethics Reference number**: 19/LO/1597 **Participant ID**: <<participant ID>> / barcode

If you are willing to take part in the SAFER Trial, please read the following statements and if you agree, sign and date overleaf.

| | |
|---|---|
| 1 | I have read and understood the Participant Information Sheet version XX, dated DATE (NAME) for the above trial. I have had the opportunity to ask questions and I am satisfied with the answers and explanations provided. |
| 2 | I understand that my participation in this trial is voluntary and that I am free to withdraw at any time, without giving a reason and without my medical care or legal rights being affected. |
| 3 | I understand that information from my medical records will be available to the research team as part of the trial. |
| 4 | I consent to my trial data being linked to Hospital Episodes Statistics (HES), civil registration mortality data, Sentinel Stroke National Audit Programme (SSNAP) and Myocardial Ischaemia National Audit Project (MINAP). This may involve sharing my personal data with these bodies. I understand that information held and managed by NHS Digital and the registries may be used in order to provide information about my health status (including after my death), my GP practice and my address (should I move). I understand that these details will be used for research purposes only. It is possible that in the future the research team may need to link to another health record or registry not listed that they consider to be relevant to the purposes of the research and I agree to this. |
| 5 | I understand that sections of my medical notes or information related directly to my participation in this trial may be looked at by responsible individuals from the sponsors, regulatory authorities and research personnel where it is relevant to my taking part in this research. I give permission for these individuals to have access to my records. |
| 6 | I understand that my GP will be informed of my participation in this trial. |
| 7 | I understand that my unidentifiable trial data will be shared with other researchers, both internal and external to this trial, and with commercial partners. These parties may be outside the European Economic Area. |
| 8 | I understand that I may be contacted about future, related research studies, and that I am under no obligation to take part. |
| 9 | I agree to participate in this trial. |



Williams K, *et al. BMJ Open* 2022; 12:e065066. doi: 10.1136/bmjopen-2022-065066

*[TO BE PRINTED ON TRIAL HEADED PAPER]*

**Participant ID:** <<participant ID>> / barcode

By signing this form you are consenting that you agree with all of the statements listed, and that the details listed below are correct.

| Name of participant | Signature | Date |
|---|---|---|
| | | |

**Please check the details below and amend/complete accordingly, then return this form to the trial team using the Freepost envelope enclosed. Alternatively you can complete this consent form online – please see the covering letter enclosed for instructions. As the trial will be conducted remotely, it will be helpful if you could please supply a phone number. If you also have an email address and are happy to provide this, please do so.**

| | |
|---|---|
| Title: | |
| First name: | |
| Surname: | |
| Date of birth (dd/mm/yyyy): | |
| Gender (M/F/Mx): | |
| Address: | |
| Postcode: | |
| Home Tel.: | |
| Mobile no.: | |
| Email: | |
| NHS no: | |
| GP Practice name:<br>**Please note:** if this is **not** your current practice and you have recently moved practice, you will not be able to take part at this point. It is possible that your new practice may take part in the future. | |

*The trial team will return a copy of this consent form to your GP practice for their records. If you would like a copy of your completed consent form please contact the trial team.*

*The trial team will only use these details in order to contact you for the purposes stated.*

1x copy to be retained by the research team; 1x copy to be sent to the participant's GP practice.



Williams K, *et al. BMJ Open* 2022; 12:e065066. doi: 10.1136/bmjopen-2022-065066
