# Supplementary material for: Cluster randomised controlled trial of screening for atrial fibrillation in people aged 70 years and over to reduce stroke: protocol for the pilot study for the SAFER trial
Source: BMJ Open. 2022 Sep 9;12(9):e065066. doi: 10.1136/bmjopen-2022-065066 (PMC9472173; doi:10.1136/bmjopen-2022-065066)
Supplement: Supplementary data [file bmjopen-2022-065066supp004.pdf]

UNIVERSITY OF CAMBRIDGE
Public Health and Primary Care
The Primary Care Unit

SAFER trial

Invitation to take part in screening for atrial fibrillation to reduce your risk of stroke

Screening Information Leaflet

V1.2 11-12-2020

FUNDED BY

NIHR | National Institute for Health Research

This project is funded by the National Institute for Health Research (NIHR) [Programme Grants for Applied Research (grant reference number RP-PG-0217-20007)]. The views expressed are those of the author(s) and not necessarily those of the NIHR or the Department of Health and Social Care.

Summary

This leaflet provides information on atrial fibrillation screening (also called AF screening) for people aged 70 and over who are already taking part in the SAFER research trial.

It explains what atrial fibrillation (AF) is and what happens when you take part in screening. It is important that you make a decision whether to take up this offer of screening based on all the available information.

Please read the information contained in this leaflet carefully. It will help you make this decision. If there is anything that you do not understand or have questions about, there is information at the end for where to go to ask any questions you may have. Screening is your choice.

Williams K, *et al. BMJ Open* 2022; 12:e065066. doi: 10.1136/bmjopen-2022-065066

Atrial fibrillation

Atrial fibrillation is a heart condition that causes an irregular and often abnormally fast heart rate. In some people this can cause symptoms like:

- palpitations
- breathlessness
- dizziness (feeling faint or light-headed),

but it may not cause any symptoms at all. Some people have atrial fibrillation all the time, for others it comes and goes. This can make it difficult to detect. About 3 in 100 (3%) people aged over 70 have atrial fibrillation without knowing it.

Risks of developing atrial fibrillation

Atrial fibrillation can affect adults of any age, but it is increasingly common as people grow older.

Screening for atrial fibrillation

Screening aims to identify atrial fibrillation, which can lead to strokes, heart failure, heart attacks and premature death. About 30% of strokes occur in people who have atrial fibrillation. Treating someone with atrial fibrillation can prevent this happening. It is also possible that atrial fibrillation may cause dementia and that management may reduce the likelihood of developing dementia.

Williams K, et al. BMJ Open 2022; 12:e065066. doi: 10.1136/bmjopen-2022-065066

Screening invitation

We are inviting you because your practice told us that you are over the age of 70 and are not on long term blood thinning (anticoagulant) medication, and you gave permission for us to include you in the SAFER research trial.

If you think you already have atrial fibrillation but do not take blood thinning (anticoagulant) medication you can still choose to be screened.

You can still take part if you take aspirin or clopidogrel. These are anti-platelet medications not anticoagulants – they thin the blood in a different way.

It is up to you to decide whether to be screened, you do not have to. Screening is your choice. If you decide not to be screened, your care at your GP practice will not be affected.

Williams K, *et al. BMJ Open* 2022; 12:e065066. doi: 10.1136/bmjopen-2022-065066

Screening test – 3 weeks screening at home

Screening is done using a simple, handheld device (called an electrocardiogram or 'ECG' recorder) that records your heart rhythm. The device is painless, safe, and easy to use. You simply place your thumbs on it for 30 seconds.

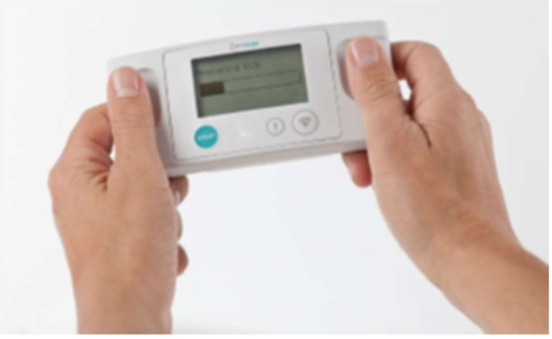

You will be contacted by the research team to check that it is convenient to send you the device. This will come with full instructions for how to use it. The device will be thoroughly cleaned before sending.

If you would like a second call from the research team after the device has arrived, to talk you through the instructions, just let them know this when they contact you about sending you the device.

You will be asked to keep the device at home for 3 weeks and use it 4 times a day and if you feel that your heart is beating irregularly.

Williams K, *et al. BMJ Open* 2022; 12:e065066. doi: 10.1136/bmjopen-2022-065066

There is a short video on the study website showing the ECG device being used https://vimeo.com/358042715. You may find it helpful to watch this although this is not essential as you will be sent full instructions for how to use it.

You are also welcome to contact the research team by phone during working hours (Monday to Friday 9am – 5pm) on **01223 763491** or by email **safer@medschl.cam.ac.uk**) if you have any questions about how to use the device.

The device stores the ECGs and transmits them over the mobile network. You do not need to have WiFi or a mobile phone to use the device.

At the end of the screening period you will need to return the device using the Freepost envelope that will be provided.

Next steps

The ECG traces will be analysed once the device is received back from you. The results will usually be available within 12 weeks from this date. If you have not heard by this time you may wish to contact the practice directly. If you move house, or GP practice, during this time please let the research team know.

Williams K, *et al. BMJ Open* 2022; 12:e065066. doi: 10.1136/bmjopen-2022-065066

It is very important that if at any point you have symptoms you seek medical help in the same way that you usually would, for example by calling 999, or contacting your GP. **Do not wait for the results of your screening test.**

Screening results

Your practice will inform you of your screening result and whether any action is required.

If you are found to have atrial fibrillation

If you are found to have atrial fibrillation, you will be invited to attend an appointment with your GP to discuss whether you need to start taking standard blood thinning (anticoagulant) medication. Your GP will help you make a decision about whether to start treatment, usually in the form of tablets.

It is likely that you will need to take this for the rest of your life. Your GP practice will arrange for appropriate monitoring of your medication.

Other health problems found by the screening

There is a very small chance that another abnormal rhythm will be detected. In this instance if any action is necessary your GP practice will contact you.

Williams K, *et al. BMJ Open* 2022; 12:e065066. doi: 10.1136/bmjopen-2022-065066

Potential benefits of being screened

If you are screened and found to have atrial fibrillation, you will be offered treatment which will greatly reduce your risk of having a stroke or heart attack and possibly dementia.

If another heart rhythm abnormality that is important to your health is diagnosed, you will be referred for further tests and / or treatment as necessary.

Potential harms of being screened

If you are found to have atrial fibrillation, and are started on treatment, this may increase your risk of bleeding, as your blood will not clot as well. This might include bleeding in the brain or the gut. You will have an opportunity when discussing treatment with your GP to weigh this risk up against the benefits of treatment.

Going through a screening process, like having any medical test, can cause anxiety in some people. You can speak to your nurse or doctor or the research team if you are anxious about screening. They can talk through any questions or concerns you have.

Being diagnosed with atrial fibrillation or another heart rhythm abnormality may affect any current or future insurance policies.

Reliability of the screening process

All the positive traces are reviewed by a cardiologist (heart specialist doctor), so the chance of an incorrect diagnosis of atrial fibrillation is very low. However, if you have the type of atrial fibrillation that comes and goes, it is possible that may not be detected if the ECG recording occurs at a point where atrial fibrillation is not present.

Storage of ECGs at the end of screening

They will be held on a secure database. If any of your ECGs show atrial fibrillation or another important abnormality they will also be stored by your practice. For more information about how we protect your data please refer to the participant information sheet sent by your practice at the start of the study. This is also available on the study website.

Williams K, *et al. BMJ Open* 2022; 12:e065066. doi: 10.1136/bmjopen-2022-065066

More information

If you have any questions about atrial fibrillation please visit the NHS Choices website https://www.nhs.uk/conditions/atrial-fibrillation

Alternatively you can speak to your GP.

If you have any questions about the SAFER Trial please visit the Trial website https://www.safer.phpc.cam.ac.uk/ or contact the research team.

Phone: contact us during working hours (Monday to Friday 9am – 5pm) on **01223 763491**. If you leave a message, we will respond to you at the earliest opportunity.

Email: safer@medschl.cam.ac.uk

Address:
The SAFER Trial
University of Cambridge
Primary Care Unit
Strangeways Research Laboratory
2 Worts Causeway
Cambridge
CB1 8RN

Williams K, *et al. BMJ Open* 2022; 12:e065066. doi: 10.1136/bmjopen-2022-065066

If you would like to take part in screening for atrial fibrillation, please see the accompanying letter for details of what you need to do next.

Williams K, *et al. BMJ Open* 2022; 12:e065066. doi: 10.1136/bmjopen-2022-065066

SAFER
trial

Williams K, *et al. BMJ Open* 2022; 12:e065066. doi: 10.1136/bmjopen-2022-065066
