# Supplementary material for: Cluster randomised controlled trial of screening for atrial fibrillation in people aged 70 years and over to reduce stroke: protocol for the pilot study for the SAFER trial
Source: BMJ Open. 2022 Sep 9;12(9):e065066. doi: 10.1136/bmjopen-2022-065066 (PMC9472173; doi:10.1136/bmjopen-2022-065066)
Supplement: Author's manuscript [file bmjopen-2022-065066.draft_revisions.pdf]

BMJ Open

BMJ Open is committed to open peer review. As part of this commitment we make the peer review history of every article we publish publicly available.

When an article is published we post the peer reviewers' comments and the authors' responses online. We also post the versions of the paper that were used during peer review. These are the versions that the peer review comments apply to.

The versions of the paper that follow are the versions that were submitted during the peer review process. They are not the versions of record or the final published versions. They should not be cited or distributed as the published version of this manuscript.

BMJ Open is an open access journal and the full, final, typeset and author-corrected version of record of the manuscript is available on our site with no access controls, subscription charges or pay-per-view fees (http://bmjopen.bmj.com).

If you have any questions on BMJ Open's open peer review process please email info.bmjopen@bmj.com

BMJ Open

A cluster randomised controlled trial of screening for atrial fibrillation in people aged 70 years and over to reduce stroke: protocol for the pilot study for the SAFER trial

Journal:	*BMJ Open*
Manuscript ID	bmjopen-2022-065066
Article Type:	Protocol
Date Submitted by the Author:	25-May-2022
Complete List of Authors:	Williams, Kate; University of Cambridge Primary Care Unit, Strangeways Research Laboratory, Department of Public Health and Primary Care<br>Modi, Rakesh; University of Cambridge Primary Care Unit, Strangeways Research Laboratory, Department of Public Health and Primary Care<br>Dymond, Andrew; University of Cambridge Primary Care Unit, Strangeways Research Laboratory, Department of Public Health and Primary Care<br>Hoare, Sarah; THIS Institute, University of Cambridge<br>Powell, Alison; THIS Institute, University of Cambridge<br>Burt, Jenni; THIS Institute, University of Cambridge<br>Edwards, Duncan; University of Cambridge Primary Care Unit, Strangeways Research Laboratory, Department of Public Health and Primary Care<br>Lund, Jenny; University of Cambridge Primary Care Unit, Strangeways Research Laboratory, Department of Public Health and Primary Care<br>Johnson, Rachel; University of Bristol Medical School<br>Lobban, Trudie; Heart Arrhythmia Alliance<br>`Lown, Mark; University of Southampton School of Primary Care Population Sciences and Medical Education, Primary Care and Population Sciences<br>Sweeting, Michael J.; University of Leicester Department of Health Sciences, Department of Health Sciences, University of Leicester<br>Thom, H; University of Bristol Medical School<br>Kaptoge, Stephen; University of Cambridge Department of Public Health and Primary Care, Cardiovascular Epidemiology Unit, Department of Public Health and Primary Care<br>Fusco, Francesco; University of Cambridge Primary Care Unit, Strangeways Research Laboratory, Department of Public Health and Primary Care<br>Morris, Stephen; University of Cambridge Primary Care Unit, Strangeways Research Laboratory, Department of Public Health and Primary Care<br>Lip, Gregory; Liverpool Centre for Cardiovascular Science, University of Liverpool, Liverpool John Moores University and Liverpool Heart & Chest Hospital, Liverpool; Aalborg University Department of Clinical Medicine<br>Armstrong, Natalie; University of Leicester Department of Health Sciences, Department of Health Sciences, University of Leicester, George Davies Centre<br>Cowie, Martin; Royal Brompton Hospital, Guy’s & St Thomas’ NHS

Foundation Trust; Kings College London Faculty of Life Sciences and Medicine; Imperial College London Faculty of Medicine
Fitzmaurice, David; University of Warwick Warwick Medical School, Health Sciences
Freedman, Ben; The University of Sydney Charles Perkins Centre, ben.freedman@sydney.edu.au
Griffin, Simon; University of Cambridge Primary Care Unit, Strangeways Research Laboratory, Department of Public Health and Primary Care; MRC Epidemiology Unit, School of Clinical Medicine, University of Cambridge
Sutton, Stephen; University of Cambridge Primary Care Unit, Strangeways Research Laboratory, Department of Public Health and Primary Care
Hobbs, Richard; University of Oxford Nuffield Department of Primary Care Health Sciences, Nuffield Department of Primary Care Health Sciences
McManus, Richard; University of Oxford Nuffield Department of Primary Care Health Sciences, Nuffield Department of Primary Care Health Sciences
Mant, Jonathan; University of Cambridge Primary Care Unit, Strangeways Research Laboratory, Department of Public Health and Primary Care
SAFER Authorship Group, The; University of Cambridge Primary Care Unit, Strangeways Research Laboratory, Department of Public Health and Primary Care

Keywords: Adult cardiology < CARDIOLOGY, PUBLIC HEALTH, PRIMARY CARE, STROKE MEDICINE

SCHOLARONE™ Manuscripts

A cluster randomised controlled trial of screening for atrial fibrillation in people aged 70 years and over to reduce stroke: protocol for the pilot study for the SAFER trial

Corresponding author

Dr Rakesh N Modi

Primary Care Unit, Department of Public Health and Primary Care, University of Cambridge, Strangeways Research Laboratory, 2 Worts' Causeway, Cambridge, UK, CB1 8RN

rnm30@medschl.cam.ac.uk

Authors

1. Dr Kate Williams, kmw36@medschl.cam.ac.uk[1]
2. Dr Rakesh N Modi, rnm30@medschl.cam.ac.uk[1]
3. Andrew Dymond, ad486@medschl.cam.ac.uk[1]
4. Dr Sarah Hoare, sarah.hoare@thisinstitute.cam.ac.uk[2]
5. Dr Alison Powell, alison.powell@thisinstitute.cam.ac.uk[2]
6. Dr Jenni Burt, jenni.burt@thisinstitute.cam.ac.uk[2]
7. Dr Duncan Edwards, dae31@medschl.cam.ac.uk[1]
8. Dr Jenny Lund, jl897@medschl.cam.ac.uk[1]
9. Dr Rachel Johnson, rachel.johnson@bristol.ac.uk[3]
10. Ms Trudie Lobban, trudie@heartrhythmalliance.org[4]
11. Dr Mark Lown, M.Lown@soton.ac.uk[5]
12. Dr Mike Sweeting, michael.sweeting@le.ac.uk[6]
13. Dr Howard Thom, howard.thom@bristol.ac.uk[3]

For peer review only - http://bmjopen.bmj.com/site/about/guidelines.xhtml

14. Dr Stephen Kaptoge, skk22@medschl.cam.ac.uk[7]

15. Dr Francesco Fusco, ff346@medschl.cam.ac.uk[1]

16. Professor Stephen Morris, sm2428@medschl.cam.ac.uk[1]

17. Professor Gregory Y.H. Lip, gregory.lip@liverpool.ac.uk[8]

18. Professor Natalie Armstrong, natalie.armstrong@le.ac.uk[6]

19. Professor Martin R Cowie, martin.cowie@kcl.ac.uk[9]

20. Professor David Fitzmaurice, D.Fitzmaurice@warwick.ac.uk[10]

21. Professor Ben Freedman, ben.freedman@sydney.edu.au[11]

22. Professor Simon J Griffin, profgp@medschl.cam.ac.uk[1, 12]

23. Professor Stephen Sutton, srs34@medschl.cam.ac.uk[1]

24. Professor F.D. Richard Hobbs, richard.hobbs@phc.ox.ac.uk[13]

25. Professor Richard J McManus, richard.mcmanus@phc.ox.ac.uk[13]

26. Professor Jonathan Mant, jm677@medschl.cam.ac.uk[1]

27. The SAFER author group

1. Primary Care Unit, Department of Public Health and Primary Care, University of Cambridge, Strangeways Research Laboratory, 2 Worts' Causeway, Cambridge, UK, CB1 8RN
2. THIS Institute (The Healthcare Improvement Studies Institute), University of Cambridge, Clifford Allbutt Building, Cambridge Biomedical Campus, Cambridge, UK, CB2 0AH
3. Bristol Medical School, University of Bristol, Canynge Hall, 39 Whatley Road, Bristol, UK, BS8 2PS
4. Heart Arrhythmia Alliance, Unit 6B, Essex House, Cromwell Business Park, Chipping Norton, UK, OX7 5SR
5. Primary Care and Population Sciences, Faculty of Medicine, University of Southampton, Aldermoor Health Centre, Aldermoor Close, Southampton, UK, SO16 5ST
6. Department of Health Sciences, University of Leicester, George Davies Centre, University Road, Leicester, UK, LE1 7RH
7. Cardiovascular Epidemiology Unit, Department of Public Health and Primary Care, University of Cambridge, East Forvie Building, Cambridge Biomedical Campus, Cambridge, UK, CB2 0SR
8. Liverpool Centre for Cardiovascular Science, University of Liverpool, Liverpool John Moores University and Liverpool Heart & Chest Hospital, Liverpool, UK; Department of Clinical Medicine, Aalborg University, Denmark.
9. Royal Brompton Hospital, Guy's & St Thomas' NHS Foundation Trust, Sydney Street, London SW3 6NP; Faculty of Life Sciences & Medicine, King's College London, London,

For peer review only - http://bmjopen.bmj.com/site/about/guidelines.xhtml

UK; Faculty of Medicine, National Heart & Lung Institute, Imperial College London, London, UK

10. Health Sciences, Warwick Medical School, University of Warwick, Coventry, UK, CV4 7AL
11. Heart Research Institute, University of Sydney, Room 3114, Level 3 East, D17 - Charles Perkins Centre, NSW, Australia, 2006
12. MRC Epidemiology Unit, School of Clinical Medicine, University of Cambridge, Cambridge Biomedical Campus, Cambridge, UK, CB2 0SL
13. Nuffield Department of Primary Care Health Sciences, University of Oxford, Radcliffe Observatory Quarter, Woodstock Road, Oxford, UK, OX2 6GG

For peer review only

For peer review only - http://bmjopen.bmj.com/site/about/guidelines.xhtml

ABSTRACT

Introduction

Atrial fibrillation (AF) is a common arrhythmia associated with 30% of strokes, as well as other cardiovascular disease, dementia and death. AF meets many criteria for screening, but there is limited evidence that AF screening reduces stroke. Consequently, no countries recommend national screening programmes for AF. The Screening for Atrial Fibrillation with ECG to Reduce stroke (SAFER) trial aims to determine whether screening for AF is effective at reducing risk of stroke. The aim of the pilot study is to assess feasibility of the main trial and inform implementation of screening and trial procedures.

Methods and analysis

SAFER is planned to be a pragmatic randomised controlled trial (RCT) of over 100,000 participants aged 70 years and over, not on long term anticoagulation therapy at baseline, with an average follow-up of five years. Participants are asked to record four traces every day for three weeks on a hand-held single-lead ECG device. Cardiologists remotely confirm episodes of AF identified by the device algorithm, and general practitioners follow-up with anticoagulation as appropriate. The pilot study is a cluster RCT in 36 UK general practices, randomised 2:1 control to intervention, recruiting approximately 12,600 participants. Pilot study outcomes include AF detection rate, anticoagulation uptake, and other parameters to incorporate into sample size calculations for the main trial. Questionnaires sent to a sample of participants will assess impact of screening on psychological health. Process evaluation and qualitative studies will underpin implementation of screening during the main trial. An economic evaluation using the pilot data will confirm whether it is plausible that screening might be cost-effective.

Ethics and dissemination

The London – Central Research Ethics Committee (19/LO/1597) and Confidentiality Advisory Group (19/CAG/0226) provided ethical approval. Dissemination will be via publications, patient-friendly summaries, reports and engagement with the UK National Screening Committee.

Trial registration number: ISRCTN72104369

KEYWORDS

Atrial fibrillation; screening; randomised controlled trial; primary care; stroke prevention

For peer review only

For peer review only - http://bmjopen.bmj.com/site/about/guidelines.xhtml

Word count: 3653 (excluding figures/tables/box and acknowledgements onwards)

For peer review only

For peer review only - http://bmjopen.bmj.com/site/about/guidelines.xhtml

ARTICLE SUMMARY

Strengths and limitations of this study

- SAFER is a large multicentre pragmatic randomised controlled trial (RCT) planned to be the largest trial of atrial fibrillation (AF) screening that has been performed.
- This internal pilot study will have good external validity, providing data on parameters for an AF screening programme in real-world conditions.
- The process evaluation of the pilot study will inform the implementation of a large-scale AF screening programme.
- Participant recruitment prior to cluster randomisation will ensure that intervention and control participants are similar, and are likely to take up screening if offered it.
- Despite the fact that anticoagulation is indicated in some people under 70, the SAFER trial is not screening in this age group.

INTRODUCTION

Atrial fibrillation (AF) is a cardiac arrhythmia present in approximately 10% of people aged over 65 years.[1] AF is increasing in prevalence,[2] and is associated with a five-fold increase in the risk of stroke,[3] as well as other negative health outcomes (such as heart failure, dementia and death).[4-8] Whilst 30% of strokes are associated with AF, 10% of strokes occur in people unaware that they have AF because it can be asymptomatic, intermittent ('paroxysmal AF') and/or undiagnosed.[9-12] AF-related strokes tend to be more severe than strokes due to other causes, imposing burdens on patient, family, and health and social care systems. [10 13]

AF is diagnosed on an electrocardiogram (ECG).[14 15] This has traditionally been achieved by a health professional interpreting a 12-lead ECG. However, 30 seconds on a single-lead ECG is now regarded as sufficient to diagnose AF.[16-20] Furthermore, acceptable and accessible portable technologies such as wearable patches, smart watches, and hand-held devices are available that can test for AF repeatedly over longer periods of time. [21 22] These technologies are sensitive to AF,[23] and can detect paroxysmal AF.[21 24]

Treatment with oral anticoagulation can effectively[25-27] and cost-effectively[28 29] reduce risk of stroke associated with AF, especially when part of an integrated care or holistic approach to AF management, as advocated in guidelines.[30 31] However, a sizeable minority of eligible patients are not taking anticoagulants.[21 32-36] With non-Vitamin-K antagonist oral anticoagulants (NOACs; also

For peer review only - http://bmjopen.bmj.com/site/about/guidelines.xhtml

called direct oral anticoagulants, DOACs) that require substantially less monitoring, and stronger recommendations for anticoagulation in clinical guidelines,[19 31] the rates of anticoagulation are increasing, but remain suboptimal.[37-39]

Undiagnosed AF is common and can be detected with simple and portable technology, and there are effective treatments available.[18 40-42] AF screening therefore fulfils many of the criteria for initiating a national systematic screening programme.[21 40 43 44]

However, no countries endorse national AF screening programmes.[14 31 33 45] Until recently, there was no evidence from randomised controlled trials of the impact of AF screening on stroke and mortality.[33] Two trials of different approaches to AF screening published in 2021 showed promising, but inconclusive results.[46-49] Both recruited much smaller numbers than is planned for SAFER (approximately 28000 for STROKESTOP and 6000 for LOOP).[46-49] Neither showed a reduction in ischaemic stroke associated with screening although STROKESTOP reported a reduction in a composite endpoint (ischaemic or haemorrhagic stroke, systemic embolism, death, and hospitalisation for bleeding). As a result in early 2022 the US Preventive Services Task Force (USPSTF) did not change its previous recommendation that there was insufficient evidence to determine whether there was greater benefit than harm for ECG screening for AF. Thus, evidence is required from a much larger randomised trial to inform guidelines and national screening body recommendations, a gap that SAFER is intended to fill.

The SAFER trial is a large, pragmatic, open-label, primary care-based RCT which will recruit around 100,000 participants and assess whether screening for AF is effective and cost-effective at reducing stroke and other outcomes.[50] It will randomise participants after consent and will investigate ways to improve implementation of screening. It will use intermittent monitoring via hand-held ECGs which will detect higher-burden AF associated with higher clinical risk than continuous monitoring.[48] It will examine harms as well as benefits of an AF screening programme.[45]

The internal pilot study detailed in this protocol, starting in March 2021, is a cluster RCT in 36 clusters (general practices), recruiting 12,600 participants who will be followed up during the main trial. The objectives of the internal pilot study are to assess intermediate outcomes such as AF detection rate and anticoagulation rate, reduce uncertainty concerning key parameters for the design, conduct and sample size calculations for the main trial, examine the psychological impact of screening, and investigate ways to optimise the delivery of the AF screening intervention.

For peer review only - http://bmjopen.bmj.com/site/about/guidelines.xhtml

For peer review only

METHODS AND ANALYSIS

Aim

To inform a decision to proceed to the main trial taking account of key intermediate outcomes (AF detection rate; anticoagulation uptake in screen detected AF), an economic analysis, and a revised sample size calculation. Also to assess any psychological impact of screening, and draw lessons for how best to implement screening in the main trial.

Design

A pragmatic, primary care-based, multi-centre, two-parallel arm, open-label, practice-level cluster RCT which aims to recruit 12,600 participants from 36 practices in a 2:1 ratio of usual care (control) to screening (intervention). Participants will be followed up immediately for pilot study outcomes, and also for an average of five years for main trial outcomes. There will be an embedded process evaluation and qualitative studies, and an economic evaluation.

Participants and setting

Participating practices will be drawn from a range of UK urban and rural settings, serving patients with a variety of different health and social needs. The vast majority of the UK population is registered with a practice that provides most AF care with referral to secondary care only for more complex cases.[19]

Eligibility

Participants

Broad eligibility criteria have been employed to maximise eternal validity (**Table 1**).

Table 1. Eligibility criteria for participants in the SAFER pilot and main trial

Inclusion criteria	Exclusion criteria (as coded on the primary care health record)
Participant has given valid informed consent	On long-term anticoagulation therapy
Aged 70 years or older	On the practice palliative care register
	Resident in a nursing or care or residential home
	Consented to another trial that will affect participation in SAFER
	Non-UK resident

For peer review only - http://bmjopen.bmj.com/site/about/guidelines.xhtml

According to guidelines, the vast majority of people aged 70 years or older with AF should be offered anticoagulation.[19] Participants with an existing diagnosis of AF but who are not being prescribed anticoagulation are included because screening these participants for AF may encourage anticoagulation use.[42 46 51]

Patients coded as resident in a nursing / care / residential home in the electronic search of patient records will be excluded due to practical difficulties.

Patients taking part in another trial will be excluded if participation in both trials could compromise either trial or affect patients' safety.

Recruitment

Practices

Practice recruitment will be managed by the National Institute for Health and Care Research (NIHR) Clinical Research Network (CRN) – a national network that coordinates and supports research delivery. The CRN will approach practices with information about the trial. Practices will express interest via an online form.

Participants

The practice will send approximately 1,200 randomly selected eligible patients an invitation pack consisting of a participant information sheet (PIS), consent form and Freepost envelope (see **Appendix A to C**). In initial practices a negative reply slip will be included in the pack so that reasons for non-participation can be analysed. Recruitment demographics will be monitored. If certain populations, e.g. the very elderly, are underrepresented, they will be over-sampled. The exact number invited will vary between practices to achieve recruitment targets based on their characteristics and any associations with recruitment (e.g. more invitations to people in more deprived areas).

To facilitate convenience, participants will have the option to return the consent form in a Freepost envelope, or to provide consent online. Reminder invitations, emails, Short Message Service (SMS), and/or invitation of additional eligible patients may be utilised if response rates are poor.

For peer review only - http://bmjopen.bmj.com/site/about/guidelines.xhtml

Randomisation and allocation

On the day after the recruited and consented participant target number is reached for a practice (350 participants), we will close recruitment and the practice will be randomised, stratified by practice location deprivation score[52] and prevalence of AF reported in the Quality and Outcomes Framework. No recruitment will take place in a practice once randomisation has occurred.

Randomisation will be implemented using a secure online randomisation system (Sortition®[53]) hosted by the University of Oxford Clinical Trials Unit (CTU). Practices will be randomised using random permuted blocks within 9 strata corresponding to 3 groups (tertiles) of practice location deprivation score and 3 groups (tertiles) of practice-level prevalence of AF. The block sizes will be known only to the trial statistician and the randomisation system programmer. All activity on the programme will have an audit trail.

Blinding of allocation to the trial team and to the practices will not be possible.

Intervention development

The screening intervention was developed with a range of stakeholders that included patient associations, patients, screening policy makers, GPs, and researchers. The intervention was tested in a feasibility study in 10 practices, which demonstrated that the intervention was feasible and acceptable to participants and practice staff.[54] In this feasibility study, practice staff conducted screening consultations in which participants were instructed how to use the ECG device. In response to the Covid-19 pandemic, a second feasibility study was undertaken in three practices, which showed that a 'remote model' of delivery of the intervention was feasible: participants could be instructed on how to use the ECG device through written instructions and video, and with optional telephone support from the study administrative team. This model ensured a low risk of Covid-19 transmission and reduced workload for primary care. Training of practices was also successfully delivered remotely. This included training on how to manage and discuss results with participants and online anticoagulation training to manage participants in line with current guidelines.[19 55]

The final intervention model is summarised in the logic model in **Figure 1**.

<<Figure 1. Logic model of the intervention in the SAFER trial>>

For peer review only - http://bmjopen.bmj.com/site/about/guidelines.xhtml

Screening intervention

Participants in intervention practices will receive an invitation to screening. Those who accept this will receive a call from the study team to arrange home delivery of the single-lead ECG device and written/video instructions, and to offer a subsequent screening consultation if required to provide support. In this consultation, the participants will be guided on use of the device and with the help of test ECG traces, how to produce a trace of acceptable quality.

Participants will undertake three weeks of intermittent screening (four 30 second traces each day) as well as when experiencing symptoms (e.g. palpitation, dizziness) using the portable Zenicor device (www.zenicor.com). They will transmit their ECG recordings via mobile network to a remote database by pressing a button on the device. If no traces have been received, or if more than 25% of traces are tagged by the algorithm as low quality, the trial team will contact the participant to offer further support.

Participants will be provided with a freepost envelope and asked to return the Zenicor device to the trial team at the end of the screening period.

Zenicor device

The screening device being used is the Zenicor hand-held single-lead ECG device. This device is usable in any location, allows repeated ECGs, and can store and transmit multiple ECG traces to a central system for analysis.[21 24] Photoplethysmography[56] and blood-pressure machines[57] have not proved accurate enough, and stakeholder discussion deemed patches less practical. The diagnostic model of the Zenicor device, its associated diagnostic algorithms, and subsequent cardiologist review have been used successfully at scale in the STROKESTOP AF screening trial in over 7000 participants, and showed a sensitivity of 98% and specificity of 92%.[42 58]

A photograph of the Zenicor device is shown in **Figure 2**.

<<Figure 2. Zenicor hand-held ECG device used to screen for AF in the SAFER trial>>

Screening results

For peer review only - http://bmjopen.bmj.com/site/about/guidelines.xhtml

A proprietary algorithm will analyse the ECG traces and place a digital flag on ECGs that might show AF. These will be reviewed by a cardiologist or cardiac technician who will determine whether AF or any other important rhythm disturbance is present. If there is uncertainty, the trace will be reviewed by another cardiologist. A confirmatory 12-lead ECG is not required.[59] The cardiologists will create a report with recommendations for the GP. Possible results are shown in **Table 2**.

Table 2 Categories of screening results as reported to general practices in the SAFER trial

**Diagnosis**	**Definition**
**1. AF ≥ 30 seconds**	AF is observed for a continuous period of 30 seconds. Sufficient readable beats (i.e. disregarding poor quality sections of an ECG) show AF
**2. Cannot exclude AF ≥ 30 seconds**	Indeterminate result – usually due to poor quality ECG traces
**3. Other significant arrhythmia**	This may include, but is not limited to:<br>➢ 2nd/3rd degree heart block<br>➢ Ventricular tachycardia<br>➢ Supraventricular tachycardia<br>➢ Any other significant arrhythmia
**4. No AF ≥ 30 seconds detected**	This will include, but is not limited to:<br>➢ Sinus rhythm<br>➢ AF < 30 seconds<br>➢ Bradycardia<br>➢ Ectopic beats
**5. Screening failure**	Unable to record any ECGs of sufficient quality for review

The trial team will send the screening results to the practice, including copies of relevant ECG traces for positive (AF or other) diagnoses. The GP can access ECG traces and reports for all of their patients freely via the Zenicor web-based system. Practice staff will notify participants of their screening result.

For results 1-3 (table 2), the practices will offer participants a consultation to discuss the result and its appropriate management. See **Figure 3** for a trial schematic.

<<Figure 3. SAFER Trial schematic>>

Outcomes

Primary and secondary outcomes are shown in **Box 1**. The internal pilot will specifically report on outcomes that are relevant for consideration of continuation of the trial. Participants in the internal pilot study will also be followed-up for an average of five years for main trial outcomes. The process

For peer review only - http://bmjopen.bmj.com/site/about/guidelines.xhtml

evaluation during the pilot (protocol to be published separately) will report outcomes to guide the successful delivery of the SAFER main trial and a national-scale AF screening programme.

A random sample of participants stratified by age and sex in both intervention and control arms will be sent questionnaires to assess possible psychological effects of screening. Qualitative work will also contribute to understanding the benefits and harms of screening, and participant experience.

Box 1. Primary and secondary outcomes assessed in the SAFER internal pilot study

Primary outcome:

- Atrial fibrillation
  - In intervention practices: the number of participants that had AF detected through screening.
  - In intervention and control practices: the number of newly detected AF patients in intervention practices compared with control practices.

Secondary outcomes:

- Uptake of anticoagulation
  - Proportion of participants with AF detected through screening in intervention practices who were started on anticoagulation.
  - Number of participants with newly detected AF that were started on anticoagulation in intervention and control practices.

- Parameters to refine the sample size calculation for the main trial (current assumptions in parentheses)
  - Proportion of consented participants in intervention practices who are screened over the screening period (85%)
  - Proportion of screened patients in whom newly diagnosed AF is detected (3%)
  - Proportion of participants with newly diagnosed AF from screening who commence anticoagulation (80%)
  - Proportion of participants with a known diagnosis of AF that is detected by screening who newly commence anticoagulation, despite previously not being prescribed anticoagulation (55%)

Sample size

Sample size calculations are based on 350 consented participants from each of the 12 intervention and 24 control practices, and the assumption that 85% of participants per intervention practice will be screened. This will provide a 90% power at 5% significance level to detect a 1.1% absolute difference in the frequency of diagnosis of new AF between intervention and control practices,

For peer review only - http://bmjopen.bmj.com/site/about/guidelines.xhtml

assuming 3% newly diagnosed AF is detected in screened patients[42] and an intraclass correlation coefficient of 0.001.

Sending the heath questionnaire to 1,800 participants will give us 90% power to detect a 4 point difference in the Spielberger questionnaire, assuming 60% respond – the rate achieved in the SAFE trial.[59]

Data collection

Baseline data collection

Baseline data detailed in **Table 3** will be collected from the GP electronic medical records for all individuals who have consented to participate in the trial.

Table 3. Baseline participant data to be collected for the SAFER pilot study

Category	Variable collected
**Demographics**	Age
	Sex
	Ethnicity
	Index of Multiple Deprivation (IMD) based on participant postcode
**Comorbidities**	Atrial fibrillation
	Stroke or transient ischaemic attack
	Coronary heart disease
	Peripheral arterial disease
	Heart failure
	Hypertension
	Diabetes mellitus
	Stroke or transient ischaemic attack
	Dementia
	Depression
**Clinical scores and indices**	CHA_2DS_2-VASc score
	HAS-BLED score
	ORBIT score
	Frailty index

For peer review only - http://bmjopen.bmj.com/site/about/guidelines.xhtml

**Other variables**	Height
	Weight
	Alcohol intake
	Smoking status
	Severe acute respiratory syndrome coronavirus 2 PCR result

Follow-up data

This section excludes outcomes for the main trial, which will be detailed in the main trial protocol.

Atrial fibrillation:

1. New diagnoses of AF picked up in both intervention and control practices since screening initiation using GP electronic data
2. Positive diagnoses of AF identified by screening (intervention practices only)

Uptake of anticoagulation:

1. For patients diagnosed with AF, whether or not they are prescribed anticoagulation (intervention and control practices) using GP electronic data
2. Initiation of anticoagulation in AF detected through screening programme (intervention practices only)

Process variables:

1. Whether patients agree to screening (intervention practices only)
2. Whether patients are screened (intervention practices only)

Psychological outcomes:

The psychological effects of screening and impact on functional status will be assessed by comparing responses to the Spielberger state anxiety inventory (SAI) short form,[60] EQ5-D-5L[61] and Short-form 8 (SF-8).[62] Changes in responses over time in both groups will be quantified as well as comparisons of responses according to uptake and outcome of screening. These generic measures may be relatively insensitive to some potential specific impacts of screening for atrial fibrillation, but as they do not include reference to the screening programme they enable comparison between screening and control groups. Furthermore, they facilitate comparisons with screening programmes for other conditions, and with other unrelated health service interventions. The questionnaires will be posted to a random (MS Access RND function) sample of participants (126 per intervention practice and 36

For peer review only - http://bmjopen.bmj.com/site/about/guidelines.xhtml

per control practice, matched for age (70 to 73; 74 to 77; 78 years and over) and sex in six groups. The target numbers in the intervention arm are raised from our sample size calculation to increase the data available from participants who screen positive. Questionnaires will be posted to the screening group alone at baseline (pre-invitation to screening). Both groups will receive questionnaires after 8 weeks and 6 months.

Data management

Data sent from practices to the trial team will be labelled with participant ID number (link-anonymisation), initials and partial date of birth. The local investigator at each site is responsible for case report form (CRF) integrity. We will offer secure online data capture (including e-consent), using an established secure system that complies with sponsor security policies (Qualtrics[63]).

ECG traces on the Zenicor system will be labelled with participant ID number, initials and partial date of birth.

Participant questionnaires will be link-anonymised and returned to the trial team by post or online prior to checking and entering.

Participant identifiable data will be stored, handled and processed securely and confidentially, in accordance with sponsor data security policies, UK data laws, and ethical guidelines. Access will be restricted to specific members of the trial team. Further information is accessible on the trial website (https://www.safer.phpc.cam.ac.uk/).

Statistical analysis

Data will be analysed according to CONSORT principles and its extension for cluster trials.[64] Outcomes will be analysed using an intention-to-treat principle for primary analysis. However, as both external and internal validity are important in the pilot study, secondary analysis will be conducted according to the per-protocol principle, when necessary and justified.

The proportion of those consented who took up screening, the proportion found to have AF (both new and previously known), and proportion who were anticoagulated will be calculated. The proportion of diagnoses of new AF participants in intervention and control practices and rate of anticoagulation will be compared. Clustering by practices will be accounted for with an adjusted Chi-square test for simple comparisons and mixed effects regression models for covariates.

For peer review only - http://bmjopen.bmj.com/site/about/guidelines.xhtml

Process evaluation and qualitative work

A mixed-methods process evaluation will be conducted to explore how AF screening is delivered and perceived at practice and patient levels. Qualitative work will seek to understand participant experiences of being invited to, and taking part in, the study.

These will contribute to refining the theory of the intervention, which will help provide recommendations for an acceptable and sustainable screening programme at scale.

Economic analysis

The pilot data will be used to update a published model, composed of a decision tree followed by a Markov model.[23] The purpose of this model is to confirm that it is plausible that screening might be cost-effective using the parameters obtained in this pilot trial. All patients entering the decision tree will incur an invitation cost and the test cost will be applied to those patients who accept screening. Screen-negative patients will not accrue any additional costs and Quality Adjusted Life Years (QALYs). The remaining patients are true positive and, thus, will enter the Markov model. This model will simulate their survival trajectories accounting for their condition and, their lifetime costs and QALYs, which will be discounted at a 3.5% annual rate and half-cycle corrected.

The costs needed to implement the screening programme will be calculated using a micro-costing approach to include all the relevant costs, such as the invitation cost and cost due to the device use (e.g. shipment of the device and the training to use the device).[65] Where needed, the costs incurred by the NHS will be updated using the most recent available data, such as the British National Formulary for the cost of anticoagulant therapies.[66]

The model will be employed to perform a probabilistic analysis and compute the total costs and QALYs. The differences in costs and QALYs between the SAFER intervention and usual care will be calculated and combined to obtain the incremental cost-effectiveness ratio (ICER). Likewise, the expected value of perfect information (EVPI) will be calculated by assuming that the value of one QALY is equal to £20,000, which reflects the cost-effectiveness threshold used by the National Institute for Health and Care Excellence (NICE). Then, the EVPI will be projected to the national level considering the eligible population for the screening and assuming that the screening programme will be provided for the next 10 years.[67]

For peer review only - http://bmjopen.bmj.com/site/about/guidelines.xhtml

Management and oversight

The University of Cambridge and NHS Cambridgeshire & Peterborough Clinical Commissioning Group (CCG) are co-sponsors. The trial management group (consisting of the chief investigator and researchers from each group) and the programme steering committee (PSC), which has an independent chair and four independent members, will appraise data and decide on continuation and course of the study in consultation with the NIHR. An active risk register has been compiled in consultation with the funder and sponsors, and will be monitored and updated throughout.

Patient and public involvement

The SAFER programme has been guided since inception by patient and public representatives who participate in all-investigator meetings. Trudie Lobban, Chief Executive and Founder of the Atrial Fibrillation Association (AFA), has been involved in the development of the research from the outset as a PPI member. The AFA represents over 64,000 people with atrial fibrillation.

Additional PPI members have been recruited independently of the AFA. Many are in the age range for AF screening; some of them either have AF or have a partner with AF. The PSC has an independent lay member who is a stroke survivor.

The PPI members are consulted throughout the trial on all aspects of the research, including: possible psychological harms of screening; participant-facing documents; how to approach participants; instructing participants on trials and screening procedures; web-based materials and qualitative data-collection material. The AFA will help with dissemination of the findings through its website and members.

For peer review only

ETHICS AND DISSEMINATION

Ethics

Ethical approval

The SAFER pilot trial has received a favourable ethical opinion from the London – Central NHS Research Ethics Committee (19/LO/1597) and the Confidentiality Advisory Group (19/CAG/0226). Modifications of the full protocol are detailed in amendments. Important modifications will be communicated to the sponsors, funder, collaborators, practices, participants, trial registries and disseminators as relevant.

For peer review only - http://bmjopen.bmj.com/site/about/guidelines.xhtml

Consent

Participants will be required to provide valid written informed consent, either via post or online. Consented participants from screening practices will be approached with an offer of AF screening.

Dissemination

The study will generate peer-reviewed publications to disseminate to academics, health professionals, policy-makers, patient organisations and the print and electronic media. After publication, data may be available to others according to data sharing agreements in compliance with the funder and sponsor policies. Summary documents will be made available to participants at the end of the study. PPI groups and media engagement will help disseminate findings. Accessible reports will be generated for national screening committees, commissioners and other decision makers. Funders' reports will be submitted in accordance with their policies.

ACKNOWLEDGEMENTS

We thank the NIHR Clinical Research Network for their support recruiting practices. We also thank the participating GP practices and SAFER participants for their contributions.

COMPETING INTERESTS

JM has performed consultancy work for BMS/Pfizer and Omron. FDRH reports occasional consultancy for BMS/Pfizer, Bayer and BI over the past 5 years. NA is a member of the UK National Screening Committee's Adult Reference Group. MS is a full-time employee of AstraZeneca. MRC reports consultancy for AstraZeneca, Abbott, Medtronic, Bayer, Novartis, Boehringer-Ingelheim-Lilly Alliance, Servier & Pfizer over the past 5 years. RMc's employer the University of Oxford receives consultancy and licencing payments from Omron and Sensyne for BP telemonitoring interventions. GYHL: Consultant and speaker for BMS/Pfizer, Boehringer Ingelheim and Daiichi-Sankyo. No fees are received personally. SJG has received honoraria from Astra Zeneca for lectures at postgraduate educational meetings for primary care teams about type 2 diabetes. BF has received speaker fees, honoraria, and non-financial support from the BMS and Pfizer Alliance; grants to the Institution for investigator-initiated studies from the BMS and Pfizer Alliance; and loan devices for investigator-initiated studies from Alivecor: all were unrelated to the present study but related to screening for AF.

For peer review only

For peer review only - http://bmjopen.bmj.com/site/about/guidelines.xhtml

FUNDING

The SAFER pilot study and main trial are funded by the National Institute for Health and Care Research (NIHR) [Programme Grants for Applied Research Programme (Reference Number RP-PG-0217-20007)]. The SAFER feasibility study is funded by the NIHR [School for Primary Care Research (SPCR-2014-10043, project 410)]. SAFER is a contributor to / partner of AFFECT-EU receiving funding from the European Union's Horizon 2020 research and innovation Programme under grant agreement NO. 847770. RM and JL are supported by the Wellcome Trust as part of the Wellcome Trust PhD Programme for Primary Care Clinicians [grant number 203921/Z/16/Z]. JB, SH and AP are based in The Healthcare Improvement Studies Institute (THIS Institute), University of Cambridge. THIS Institute is supported by the Health Foundation, an independent charity committed to bringing about better health and healthcare for people in the UK. JB is supported by the Health Foundation's grant to the University of Cambridge for The Healthcare Improvement Studies (THIS) Institute (RG88620). JM and FDRH are NIHR Senior Investigators. FDRH acknowledges support from NIHR ARC OTV and Oxford BRC (OUT). RJM is an NIHR Senior Investigator and acknowledges support from NIHR ARC OTV. NA is supported by a Health Foundation Improvement Science Fellowship and also by the NIHR Applied Research Collaboration East Midlands (ARC EM). RJ is an NIHR-funded Academic Clinical Lecturer. The University of Cambridge has received salary support in respect of SJG from the NHS in the East of England through the Clinical Academic Reserve. BF received funding from the Medical Research Future Fund International Clinical Trial Collaboration Grant to perform SAFER-AUS as part of SAFER, and a NSW Health Senior Researcher Cardiovascular Grant for work in AF. All the funders and sponsors had no involvement in the development of this protocol and will have no involvement in any aspect of the study itself. The views expressed are those of the author(s) and not necessarily those of the NHS, the Wellcome Trust, the NIHR or the UK Department of Health and Social Care.

For peer review only

AUTHORS' CONTRIBUTIONS

JM is the guarantor. RNM drafted the manuscript. KW and AD are coordinating, gaining ethical approval, and helping design the study. RNM, SH, AP, JB, RJ and NA designed the process evaluation and qualitative studies. JL designed the collection and analysis of some of the pilot outcome data collection. TL is a PPI representative that has informed design, outcomes and dissemination plan. SM, FF and HT designed the economic evaluation. MS and SK designed the statistical analysis. All other authors, including JB, NA and the SAFER author group contributed to conception and design of study, applying for funding, and writing of the protocol for the ethical approval. All authors reviewed and had the option to edit the final manuscript.

For peer review only - http://bmjopen.bmj.com/site/about/guidelines.xhtml

REFERENCES

1. Fitzmaurice DA, Hobbs FD, Jowett S, et al. Screening versus routine practice in detection of atrial fibrillation in patients aged 65 or over: cluster randomised controlled trial. *Bmj* 2007;335(7616):383. doi: 10.1136/bmj.39280.660567.55 [published Online First: 2007/08/04]
2. Krijthe BP, Kunst A, Benjamin EJ, et al. Projections on the number of individuals with atrial fibrillation in the European Union, from 2000 to 2060. *Eur Heart J* 2013;34(35):2746-51. doi: 10.1093/eurheartj/eht280 [published Online First: 2013/08/01]
3. Wolf PA, Abbott RD, Kannel WB. Atrial fibrillation as an independent risk factor for stroke: the Framingham Study. *Stroke* 1991;22(8):983-8. doi: 10.1161/01.str.22.8.983 [published Online First: 1991/08/01]
4. Guha K, McDonagh T. Heart failure epidemiology: European perspective. *Current cardiology reviews* 2013;9(2):123-27.
5. Violi F, Soliman EZ, Pignatelli P, et al. Atrial Fibrillation and Myocardial Infarction: A Systematic Review and Appraisal of Pathophysiologic Mechanisms. *Journal of the American Heart Association* 2016;5(5):e003347. doi: doi:10.1161/JAHA.116.003347
6. Singh-Manoux A, Fayosse A, Sabia S, et al. Atrial fibrillation as a risk factor for cognitive decline and dementia. *European Heart Journal* 2017;38(34):2612-18.
7. Benjamin EJ, Wolf PA, D'Agostino RB, et al. Impact of atrial fibrillation on the risk of death: the Framingham Heart Study. *Circulation* 1998;98(10):946-52.
8. Burdett P, Lip GYH. Atrial fibrillation in the UK: predicting costs of an emerging epidemic recognizing and forecasting the cost drivers of atrial fibrillation-related costs. *European Heart Journal - Quality of Care and Clinical Outcomes* 2020 doi: 10.1093/ehjqcco/qcaa093
9. Perera KS, Vanassche T, Bosch J, et al. Global Survey of the Frequency of Atrial Fibrillation-Associated Stroke: Embolic Stroke of Undetermined Source Global Registry. *Stroke* 2016;47(9):2197-202. doi: 10.1161/strokeaha.116.013378 [published Online First: 2016/08/11]
10. Hannon N, Daly L, Murphy S, et al. Acute hospital, community, and indirect costs of stroke associated with atrial fibrillation: population-based study. *Stroke* 2014;45(12):3670-74.
11. Xiong Q, Proietti M, Senoo K, et al. Asymptomatic versus symptomatic atrial fibrillation: A systematic review of age/gender differences and cardiovascular outcomes. *Int J Cardiol* 2015;191:172-7. doi: 10.1016/j.ijcard.2015.05.011 [published Online First: 2015/05/15]
12. Wallenhorst C, Martinez C, Freedman B. Risk of Ischemic Stroke in Asymptomatic Atrial Fibrillation Incidentally Detected in Primary Care Compared with Other Clinical Presentations. *Thromb Haemost* 2021(EFirst) [published Online First: 30.06.2021]
13. Stewart S, Murphy NF, Walker A, et al. Cost of an emerging epidemic: an economic analysis of atrial fibrillation in the UK. *Heart* 2004;90(3):286-92. doi: 10.1136/hrt.2002.008748 [published Online First: 2004/02/18]
14. Mairesse GH, Moran P, Van Gelder IC, et al. Screening for atrial fibrillation: a European Heart Rhythm Association (EHRA) consensus document endorsed by the Heart Rhythm Society (HRS), Asia Pacific Heart Rhythm Society (APHRS), and Sociedad Latinoamericana de Estimulación Cardíaca y Electrofisiología (SOLAECE). *EP Europace* 2017;19(10):1589-623. doi: 10.1093/europace/eux177
15. Calkins H, Hindricks G, Cappato R, et al. 2017 HRS/EHRA/ECAS/APHRS/SOLAECE expert consensus statement on catheter and surgical ablation of atrial fibrillation. *Heart rhythm* 2017;14(10):e275-e444. doi: 10.1016/j.hrthm.2017.05.012 [published Online First: 2017/05/17]

For peer review only - http://bmjopen.bmj.com/site/about/guidelines.xhtml

16. Mairesse GH, Moran P, Van Gelder IC, et al. Screening for atrial fibrillation: a European Heart Rhythm Association (EHRA) consensus document endorsed by the Heart Rhythm Society (HRS), Asia Pacific Heart Rhythm Society (APHRS), and Sociedad Latinoamericana de Estimulacion Cardiaca y Electrofisiologia (SOLAECE). *Europace* 2017;19(10):1589-623. doi: 10.1093/europace/eux177 [published Online First: 2017/10/20]
17. Freedman B, Camm J, Calkins H, et al. Screening for Atrial Fibrillation: A Report of the AF-SCREEN International Collaboration. *Circulation* 2017;135(19):1851-67. doi: 10.1161/circulationaha.116.026693 [published Online First: 2017/05/10]
18. Kirchhof P, Benussi S, Kotecha D, et al. 2016 ESC Guidelines for the management of atrial fibrillation developed in collaboration with EACTS. *Eur Heart J* 2016;37(38):2893-962. doi: 10.1093/eurheartj/ehw210 [published Online First: 2016/08/28]
19. National Institute for Health and Care Excellence. Atrial fibrillation: diagnosis and management (ng196): National Institute of Health and Care Excellence, 2021.
20. Sun W, Freedman B, Martinez C, et al. Atrial Fibrillation Detected by Single Time-Point Handheld Electrocardiogram Screening and the Risk of Ischemic Stroke. *Thromb Haemost* 2021(EFirst) [published Online First: 16.08.2021]
21. Jones NR, Taylor CJ, Hobbs FDR, et al. Screening for atrial fibrillation: a call for evidence. *European Heart Journal* 2019 doi: 10.1093/eurheartj/ehz834
22. Harris K, Edwards D, Mant J. How can we best detect atrial fibrillation? *The journal of the Royal College of Physicians of Edinburgh* 2012;42:5-22.
23. Welton NJ, McAleenan A, Thom HH, et al. Screening strategies for atrial fibrillation: a systematic review and cost-effectiveness analysis. *Health Technology Assessment* 2017;21(29)
24. Fitzmaurice DA, McCahon D, Baker J, et al. Is screening for AF worthwhile? Stroke risk in a screened population from the SAFE study. *Fam Pract* 2014;31(3):298-302. doi: 10.1093/fampra/cmu011 [published Online First: 2014/04/15]
25. Kim D, Yang PS, Yu HT, et al. Risk of dementia in stroke-free patients diagnosed with atrial fibrillation: data from a population-based cohort. *Eur Heart J* 2019;40(28):2313-23. doi: 10.1093/eurheartj/ehz386 [published Online First: 2019/06/19]
26. Hart RG, Pearce LA, Aguilar MI. Meta-analysis: antithrombotic therapy to prevent stroke in patients who have nonvalvular atrial fibrillation. *Ann Intern Med* 2007;146(12):857-67. doi: 10.7326/0003-4819-146-12-200706190-00007 [published Online First: 2007/06/20]
27. Martinez C, Katholing A, Freedman SB. Adverse prognosis of incidentally detected ambulatory atrial fibrillation. A cohort study. *Thromb Haemost* 2014;112(2):276-86. doi: 10.1160/th4-04-0383 [published Online First: 2014/06/24]
28. Lopez-Lopez JA, Sterne JAC, Thom HHZ, et al. Oral anticoagulants for prevention of stroke in atrial fibrillation: systematic review, network meta-analysis, and cost effectiveness analysis. *Bmj* 2017;359:j5058. doi: 10.1136/bmj.j5058 [published Online First: 2017/12/01]
29. Yiin GS, Howard DP, Paul NL, et al. Age-specific incidence, outcome, cost, and projected future burden of atrial fibrillation-related embolic vascular events: a population-based study. *Circulation* 2014;130(15):1236-44. doi: 10.1161/circulationaha.114.010942 [published Online First: 2014/09/12]
30. Romiti GF, Pastori D, Rivera-Caravaca JM, et al. Adherence to the 'Atrial Fibrillation Better Care' Pathway in Patients with Atrial Fibrillation: Impact on Clinical Outcomes—A Systematic Review and Meta-Analysis of 285,000 Patients. *Thromb Haemost* 2021(EFirst) [published Online First: 21.05.2021]
31. Hindricks G, Potpara T, Dagres N, et al. 2020 ESC Guidelines for the diagnosis and management of atrial fibrillation developed in collaboration with the European Association for Cardio-Thoracic Surgery (EACTS): The Task Force for the diagnosis and management of atrial fibrillation of the European Society of Cardiology (ESC) Developed with the special contribution of the European Heart Rhythm Association (EHRA) of the ESC. *European Heart Journal* 2020;42(5):373-498. doi: 10.1093/eurheartj/ehaa612

For peer review only - http://bmjopen.bmj.com/site/about/guidelines.xhtml

32. Friberg L, Rosenqvist M, Lindgren A, et al. High prevalence of atrial fibrillation among patients with ischemic stroke. *Stroke* 2014;45(9):2599-605. doi: 10.1161/strokeaha.114.006070 [published Online First: 2014/07/19]
33. King S, Fitzgerald A, Bartlett C, et al. Evidence Summary for Screening for Atrial Fibrillation in Adults: External review against programme appraisal criteria for the UK National Screening Committee: UK National Screening Committee, 2019.
34. Gomez-Outes A, Lagunar-Ruiz J, Terleira-Fernandez AI, et al. Causes of Death in Anticoagulated Patients With Atrial Fibrillation. *J Am Coll Cardiol* 2016;68(23):2508-21. doi: 10.1016/j.jacc.2016.09.944 [published Online First: 2016/12/10]
35. Pisters R, Lane DA, Nieuwlaat R, et al. A novel user-friendly score (HAS-BLED) to assess 1-year risk of major bleeding in patients with atrial fibrillation: the Euro Heart Survey. *Chest* 2010;138(5):1093-100.
36. Pritchett RV, Bem D, Turner GM, et al. Improving the Prescription of Oral Anticoagulants in Atrial Fibrillation: A Systematic Review. *Thromb Haemost* 2019;119(02):294-307.
37. Holt TA, Hunter TD, Gunnarsson C, et al. Risk of stroke and oral anticoagulant use in atrial fibrillation: a cross-sectional survey. *Br J Gen Pract* 2012;62(603):e710-e17.
38. Maura G, Billionnet C, Drouin J, et al. Oral anticoagulation therapy use in patients with atrial fibrillation after the introduction of non-vitamin K antagonist oral anticoagulants: findings from the French healthcare databases, 2011–2016. *BMJ Open* 2019;9(4):e026645. doi: 10.1136/bmjopen-2018-026645
39. Lund J, Saunders CL, Edwards D, et al. Anticoagulation trends in adults aged 65 years and over with atrial fibrillation: a cohort study. *Open Heart* 2021;8(2):e001737. doi: 10.1136/openhrt-2021-001737
40. Lown M, Moran P. Should we screen for atrial fibrillation? *BMJ* 2019;364:l43. doi: 10.1136/bmj.l43
41. Lowres N, Neubeck L, Redfern J, et al. Screening to identify unknown atrial fibrillation. A systematic review. *Thromb Haemost* 2013;110(2):213-22. doi: 10.1160/th13-02-0165 [published Online First: 2013/04/19]
42. Svennberg E, Engdahl J, Al-Khalili F, et al. Mass Screening for Untreated Atrial Fibrillation: The STROKESTOP Study. *Circulation* 2015;131(25):2176-84. doi: 10.1161/circulationaha.114.014343 [published Online First: 2015/04/26]
43. Wilson JMG, Jungner G, Organization WH. Principles and practice of screening for disease. 1968
44. Public Health England. Criteria for appraising the viability, effectiveness and appropriateness of a screening programme: GOV.UK; 2015 [updated 23/10/2015. Available from: https://www.gov.uk/government/publications/evidence-review-criteria-national-screening-programmes/criteria-for-appraising-the-viability-effectiveness-and-appropriateness-of-a-screening-programme accessed 24/03/2020.
45. US Preventive Services Task Force. Screening for Atrial Fibrillation: US Preventive Services Task Force Recommendation Statement. *Jama* 2022;327(4):360-67. doi: 10.1001/jama.2021.23732
46. Svennberg E, Friberg L, Frykman V, et al. Clinical outcomes in systematic screening for atrial fibrillation (STROKESTOP): a multicentre, parallel group, unmasked, randomised controlled trial. *The Lancet* 2021 doi: https://doi.org/10.1016/S0140-6736(21)01637-8
47. Svendsen JH, Diederichsen SZ, Højberg S, et al. Implantable loop recorder detection of atrial fibrillation to prevent stroke (The LOOP Study): a randomised controlled trial. *The Lancet* 2021 doi: https://doi.org/10.1016/S0140-6736(21)01698-6
48. Freedman B, Lowres N. High-intensity atrial fibrillation screening to prevent stroke. *The Lancet* 2021 doi: https://doi.org/10.1016/S0140-6736(21)01785-2
49. Lowres N, Freedman B. Population screening for atrial fibrillation to prevent stroke. *The Lancet* 2021 doi: https://doi.org/10.1016/S0140-6736(21)01750-5

For peer review only - http://bmjopen.bmj.com/site/about/guidelines.xhtml

50. SAFER team. The SAFER Study – Screening for Atrial Fibrillation with ECG to Reduce stroke: University of Cambridge; 2019 [Available from: https://www.safer.phpc.cam.ac.uk/ accessed 25/03/2020.
51. Engdahl J, Andersson L, Mirskaya M, et al. Stepwise Screening of Atrial Fibrillation in a 75-Year-Old Population. *Circulation* 2013;127(8):930-37. doi: doi:10.1161/CIRCULATIONAHA.112.126656
52. National Statistics: Ministry of Housing CLG. English indices of deprivation, 2019: gov.uk; 2019 [Available from: https://www.gov.uk/government/statistics/english-indices-of-deprivation-2019 accessed 11/04/2022.
53. Oxford University Innovation Ltd. Sortition - clinical trial software: University of Oxford Primary Care Clinical Trials Unit; 2022 [Available from: https://www.phctrials.ox.ac.uk/software/sortition accessed 06/05/2022.
54. Mant J, Burt J, Griffin S, et al. The SAFER study (Screening for Atrial Fibrillation with ECG to Reduce stroke): is it feasible to screen for atrial fibrillation in general practice? *International Journal of Stroke* 2020;15:551.
55. National Institute for Health and Care Excellence. Atrial Fibrillation: management: clinical guideline 180: National Institute for Health and Care Excellence, 2014.
56. Perez MV, Mahaffey KW, Hedlin H, et al. Large-Scale Assessment of a Smartwatch to Identify Atrial Fibrillation. *New England Journal of Medicine* 2019;381(20):1909-17. doi: 10.1056/NEJMoa1901183
57. Gladstone DJ, Wachter R, Schmalstieg-Bahr K, et al. Screening for Atrial Fibrillation in the Older Population: A Randomized Clinical Trial. *JAMA Cardiology* 2021 doi: 10.1001/jamacardio.2021.0038
58. Friberg L, Engdahl J, Frykman V, et al. Population screening of 75- and 76-year-old men and women for silent atrial fibrillation (STROKESTOP). *EP Europace* 2012;15(1):135-40. doi: 10.1093/europace/eus217
59. Hobbs FD, Fitzmaurice DA, Mant J, et al. A randomised controlled trial and cost-effectiveness study of systematic screening (targeted and total population screening) versus routine practice for the detection of atrial fibrillation in people aged 65 and over. The SAFE study. *Health technology assessment (Winchester, England)* 2005;9(40):iii-iv, ix-x, 1-74. doi: 10.3310/hta9400 [published Online First: 2005/10/06]
60. Marteau TM, Bekker H. The development of a six-item short-form of the state scale of the Spielberger State—Trait Anxiety Inventory (STAI). *British Journal of Clinical Psychology* 1992;31(3):301-06. doi: https://doi.org/10.1111/j.2044-8260.1992.tb00997.x
61. Buchholz I, Janssen MF, Kohlmann T, et al. A Systematic Review of Studies Comparing the Measurement Properties of the Three-Level and Five-Level Versions of the EQ-5D. *PharmacoEconomics* 2018;36(6):645-61. doi: 10.1007/s40273-018-0642-5
62. Ware JE, Kosinski M, Dewey JE, et al. How to score and interpret single-item health status measures: a manual for users of the SF-8 health survey. *Lincoln, RI: QualityMetric Incorporated* 2001;15(10):5.
63. Qualtrics. QualtricsXM 2022 [Available from: https://www.qualtrics.com/uk/ accessed 15/03/2022.
64. Campbell MK, Piaggio G, Elbourne DR, et al. Consort 2010 statement: extension to cluster randomised trials. *Bmj* 2012;345:e5661. doi: 10.1136/bmj.e5661 [published Online First: 2012/09/07]
65. Drummond MF, Sculpher MJ, Claxton K, et al. Methods for the Economic Evaluation of Health Care Programmes. Oxford: Oxford: Oxford University Press 2015.
66. Joint Formulary Committee. British National Formulary (online). London, UK: London: BMJ Group and Pharmaceutical Press, 2022.
67. Office for National Statistics. Dataset: Principal projection - UK population in age groups London, UK: Officer for National Statistics; 2022 [Available from:

For peer review only - http://bmjopen.bmj.com/site/about/guidelines.xhtml

https://www.ons.gov.uk/peoplepopulationandcommunity/populationandmigration/populationprojections/datasets/tablea21principalprojectionukpopulationinagegroups accessed 11/04/2022.

SUPPLEMENTARY MATERIALS

A – Trial invite letter

B – Trial PIS

C – Trial ICF

For peer review only

For peer review only - http://bmjopen.bmj.com/site/about/guidelines.xhtml

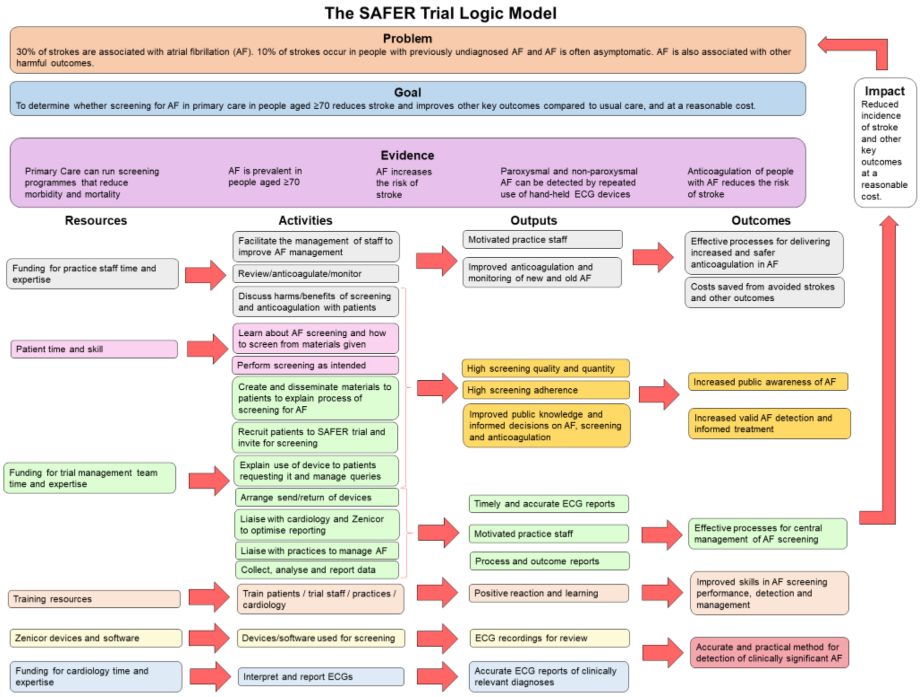

Figure 1. Logic model of the intervention in the SAFER trial

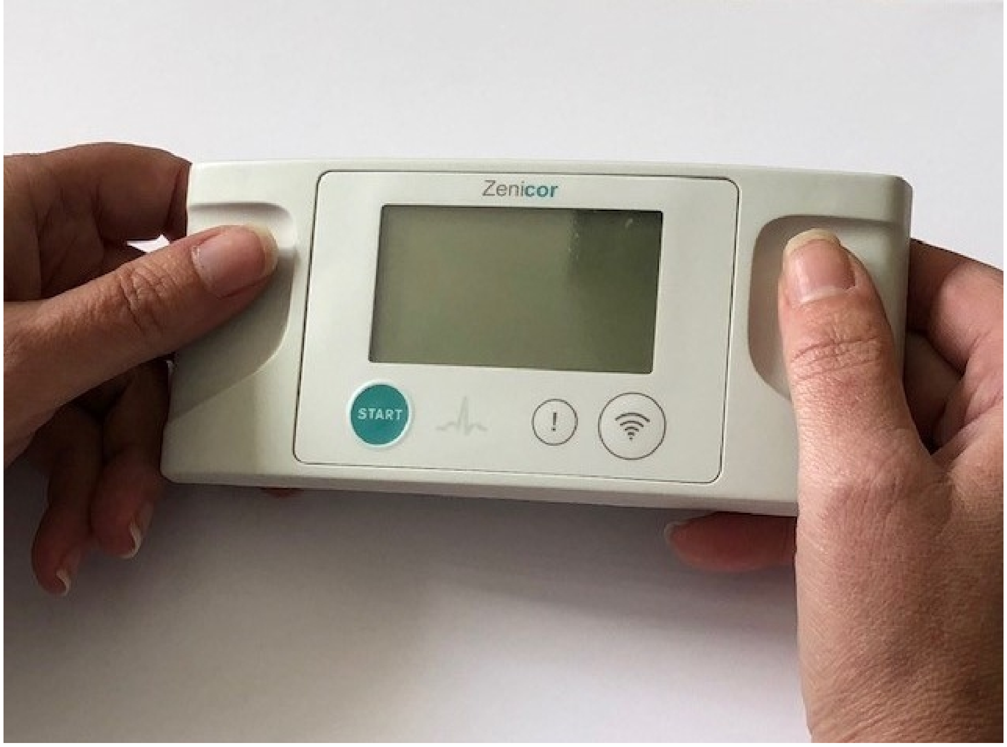

Figure 2. Zenicor hand-held ECG device used to screen for AF in the SAFER trial

225x167mm (72 x 72 DPI)

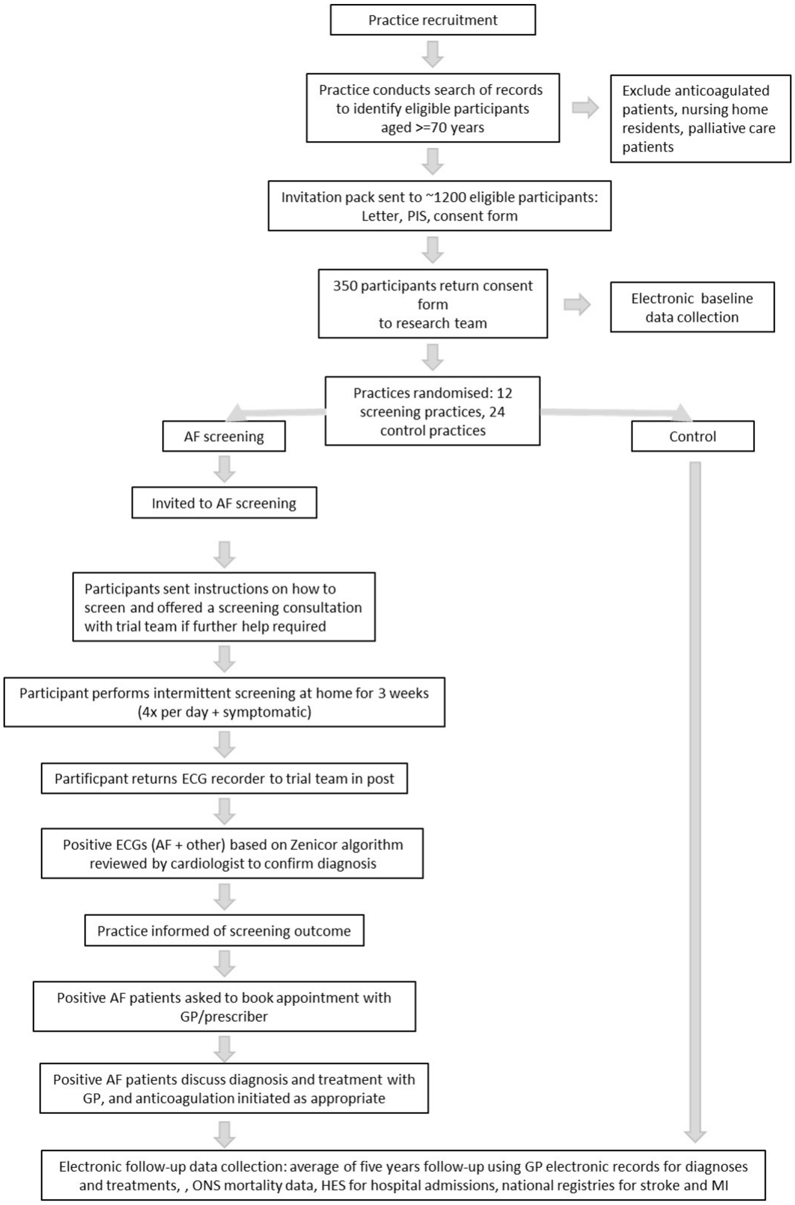

Figure 3. SAFER Trial schematic

178x271mm (150 x 150 DPI)

PRACTICE HEADED PAPER
PRACTICE ADDRESS
PRACTICE PARTNERS

Participant ID: <<Trial_ID>>

<<Title>> <<First name>> <<Surname>>
<<Address 1>>
<<Address 2>>
<<Address 3>>
<<Address 4>>
<<Address 5>>

<<Date>>

Dear <<Title>> <<First name>> <<Surname>>

For peer review only

Invitation to join a research trial:

The SAFER Trial

We are writing to let you know about a research trial that we are involved with. We are working with researchers from the University of Cambridge.

The trial aims to test whether screening at home for a common heart rate condition, Atrial Fibrillation (AF) would prevent people having strokes and heart attacks.

We are inviting you because you are in the age range where atrial fibrillation is more common. You have been selected at random from patients at your practice in this age range. You will not need to attend an appointment at the practice. You may still be able to take part if you already have atrial fibrillation.

You do not have to participate. Your decision will not affect any future health care you receive.

Enclosed with this letter is:

1. **Participant Information Sheet**
2. **Consent Form (including contact details) (complete and return to take part)**
3. **'I do not wish to take part' Reply Slip (ONLY complete and return if you DO NOT want to take part)**
4. **Freepost envelope**

Please read the **Participant Information Sheet,** the **Consent Form** and **Reply Slip** carefully. Please take your time to consider all the information. You can talk to others about your decision to take part, and contact the researchers to ask them any questions you have, or talk to your GP.

Once you have made your decision, please see instructions at the bottom of page 2 for what you need to do next. (**Note:** If the research team does not hear from you we may send you a **reminder letter**.)

For peer review only - http://bmjopen.bmj.com/site/about/guidelines.xhtml

PRACTICE HEADED PAPER
PRACTICE ADDRESS
PRACTICE PARTNERS

If you would like more information

If you have any questions please contact the SAFER Trial team by **phone** on **01223 763491** (working hours are Monday to Friday 9am-5pm, there is an answer phone on this number if they miss your call), or you can contact them by **email**, safer@medschl.cam.ac.uk. There is also more information about the trial on the website - https://www.safer.phpc.cam.ac.uk/.

Thank you for taking the time to read this letter.

Yours sincerely,

<Signature>

<Name of GP>

For peer review only

What to do next:

✓ I WOULD like to take part

If you would like to take part please complete the trial consent form. You can do this either:

Online: by following this secure link bit.ly/saferconsent and entering the code below to fill in your consent form online:

Code: <<Token>>

OR;

By post: by completing the enclosed **Consent Form**, checking that the contact details on the second page of the form are accurate. Please amend and add any if applicable. Please return the completed **Consent Form** to the research team in the Freepost envelope provided (no stamp required).

X I DO NOT want to take part

If you do not wish to take part, we would appreciate it if you would complete the **'I do not wish to take part'** Reply Slip enclosed and return it to the research team in the Freepost envelope provided (no stamp required). We would appreciate it if you would let the researchers know the reasons why you do not wish to participate in the SAFER trial.

For peer review only - http://bmjopen.bmj.com/site/about/guidelines.xhtml

UNIVERSITY OF CAMBRIDGE
Public Health and Primary Care
The Primary Care Unit

SAFER trial

The SAFER Trial –
Screening for Atrial Fibrillation with ECG to Reduce stroke

Participant Information Sheet

V1.3 03-02-2021_remote

We invite you to take part in a research trial about screening for atrial fibrillation to reduce stroke

- Before you decide whether to take part, it is important for you to understand why the research is being done and what it will involve.
- Please take your time to read the following information carefully. You can discuss it with your friends and relatives if you wish.
- You are free to decide whether or not to take part in this trial. If you choose not to take part, it will not affect the care that you receive from your doctors.
- Please don't hesitate to ask us any questions you may have, if anything is unclear, or if you would like more information.

FUNDED BY
NIHR | National Institute for Health Research

This project is funded by the National Institute for Health Research (NIHR) [Programme Grants for Applied Research (grant reference number RP-PG-0217-20007)]. The views expressed are those of the author(s) and not necessarily those of the NIHR or the Department of Health and Social Care.

For peer review only - http://bmjopen.bmj.com/site/about/guidelines.xhtml

Part 1: Trial Summary

A brief summary of the trial

- Atrial fibrillation (AF) is a common irregular heartbeat, and is a major risk factor for stroke unless treated with medication.
- We (researchers at the University of Cambridge and the University of Oxford) want to find out whether a national screening programme for atrial fibrillation (i) is possible/achievable, (ii) will prevent strokes and (iii) is good value for money for the NHS.
- We are inviting patients aged 70 years and over to take part in a research trial that aims to answer these questions. If you have atrial fibrillation you may still be able to take part.
- If you agree to take part, we will ask for your consent to give us access to relevant information from your medical and other health-related records. This will be information related to stroke and associated conditions (you can read more about this later in this information sheet). We will make sure that this information is looked after securely, remains confidential and is only used for the purposes described – all in accordance with the General Data Protection Regulations.
- Your practice may or may not offer screening for atrial fibrillation as part of the trial. If your practice does offer atrial fibrillation screening we will send you further information about this and you can choose then whether you would like to be screened or not.
- You can also visit the trial website at https://www.safer.phpc.cam.ac.uk
- If you have any questions you can phone us on **01223 763491** or email at safer@medschl.cam.ac.uk

Contents

Part 1
Trial Summary .... *Page 2*

Part 2
Further Trial Details .... *Page 3*

Part 3
Data Confidentiality .... *Page 8*

Part 4
Contact Us .... *Page 11*

How to contact us

If you have any questions you can call us on **01223 763491** (Monday to Friday 9am-5pm)

Or email us at safer@medschl.cam.ac.uk

You can also visit the trial website at www.safer.phpc.cam.ac.uk

For peer review only - http://bmjopen.bmj.com/site/about/guidelines.xhtml

Part 2: Further Trial Details

Why are we doing this trial?

Atrial fibrillation (AF) is a heart condition that causes an irregular heartbeat. It affects over 1 in 10 people over the age of 70 but does not necessarily cause symptoms.

Having atrial fibrillation increases the risk of having a stroke 5-fold, but treatment with medication can significantly lower this risk as well as lowering your risk of having a heart attack.

We want to find out whether screening people over the age of 70 years for atrial fibrillation and treating them with medication:

1. is possible/achievable in GP practices (**no GP practice visit needed**);
2. prevents the number of strokes we think it will;
3. is good value for money for the NHS.

The results will help the NHS decide whether to start a national screening programme for atrial fibrillation to reduce the number of strokes.

Why have I been invited?

You have been invited because you are in the age range where atrial fibrillation is more common. You have been selected, at random, from patients at your practice in this age range.

I have a heart condition. Can I take part?

Yes. If you have the following you can still take part:

- Atrial fibrillation and are **not** taking blood thinning (anticoagulation) medication like warfarin
- A pacemaker
- If you are taking aspirin or clopidogrel

Do I have to take part?

No. You do not have to take part. If you decide not to take part, or change your mind at any stage, your care at your GP practice will not be affected.

For peer review only - http://bmjopen.bmj.com/site/about/guidelines.xhtml

What will I have to do?

Consent

We will ask you to confirm your consent to take part. You can do this online by following the instructions in the covering letter. Alternatively, you can sign the enclosed paper consent form and post it back to us in the Freepost envelope provided.

We are asking you to give permission for specific information from your medical records to be shared with the research team at various points over the next few years. This information will be collected from your GP practice records and from other health-related records. The kind of information we will collect will include the medications you take, your use of health care services and stroke and cardiovascular disease related factors (for example weight, age, blood pressure, other medical conditions, and how well your kidneys and liver work). You will not need to do anything to provide this information. We will collect this information from everyone who consents to be part of the trial, whether they are screened for atrial fibrillation or not. Collecting this information will help us to understand whether screening for atrial fibrillation makes a difference, by comparing information about people who do and do not undergo this screening.

What else might I be invited to do?

Possibly take part in screening for atrial fibrillation

You may be invited to be screened for atrial fibrillation. This would be done at home. You would be sent the ECG recording device and instructions. There would be no need to attend an appointment at your GP practice or for anyone to visit you at home. Screening involves simply holding a small, safe, non-invasive recording device in your hands for 30 seconds at a time.

We will send you more information about what is involved and the potential benefits and harms of screening at the time if you are invited.

You are under no obligation to take part.

Not all the practices that are taking part in the trial will offer atrial fibrillation screening. Whether your practice does or not is determined by chance, so it may be that you are not invited to have screening.

For peer review only - http://bmjopen.bmj.com/site/about/guidelines.xhtml

Possibly complete questionnaires

On up to 3 separate occasions we may send you a questionnaire to complete and return in a Freepost envelope (or complete online). You are under no obligation to complete this questionnaire.

Possibly be invited to take part in other studies

Some people may be invited to take part in optional interviews and/or other studies related to atrial fibrillation and/or screening. If this is the case, you will be given further information about what these would involve. You will be free to decide at that point whether or not you want to do them.

Are there any benefits of taking part?

There may not be any direct benefit to you of taking part. However, you may find it rewarding to know that you are contributing to research that aims to prevent stroke and heart attacks in the future.

Are there any risks involved in taking part?

Identifiable medical data from your practice will be shared with the research team. All our data collection, storage and handling processes will comply with the relevant security policies and regulations. Every effort will be made to ensure the security and confidentiality of your data. Your usual medical care will not be affected by your participation in the trial.

What if I change my mind about giving access to medical and health-related records?

If you change your mind about taking part you can withdraw your consent at any time. If this is the case, please telephone us or email us using the contact details in part 4 of this information sheet. Any of your information that has been collected up to that point will be kept and used for the purposes described in this information sheet. We will not collect any further health-related information about you and your medical care at your GP practice will not be affected.

For peer review only - http://bmjopen.bmj.com/site/about/guidelines.xhtml

Who is organising and funding the trial?

The trial is being organised by the University of Cambridge working with the University of Oxford. The University of Cambridge and Cambridgeshire and Peterborough Clinical Commissioning Group (CCG) are the co-sponsors for the trial. The sponsors have overall responsibility for the conduct of the trial. It is funded by the National Institute for Health Research.

Who has reviewed / approved the trial?

To protect your interests, all research in the NHS is looked at by an independent group of people, called a Research Ethics Committee. This trial has been reviewed and given favourable opinion by the London-Central NHS Research Ethics Committee. The science has been reviewed by experts in atrial fibrillation, stroke and screening in the NHS.

How have patients and the public been involved in the trial?

Patient representatives and members of the public have been involved with the design of the trial and/or this information sheet. Patients are represented on a number of the trial research committees, and one is a co-investigator on the research grant.

What will happen if something goes wrong?

If you have any concerns about any aspect of this trial, you should ask to speak to us (the research team) and we will do our best to answer your questions:

Telephone: 01223 763491

Email: safer@medschl.cam.ac.uk

Post: The SAFER Trial, University of Cambridge, Primary Care Unit, Strangeways Research Laboratory, 2 Worts Causeway, Cambridge, CB1 8RN

If you remain unhappy and wish to complain formally, please first contact Cambridgeshire and Peterborough Clinical Commissioning Group patient experience team.

FREEPHONE: 0800 279 2535 or 01223 725 588

Email: CAPCCG.pet@nhs.net

Post: Patient Experience Team, Lockton House, Clarendon Road, Cambridge, CB2 8FH

For peer review only - http://bmjopen.bmj.com/site/about/guidelines.xhtml

Please note that due to the Covid-19 pandemic the patient experience team may not currently be able to respond to your query in a timely manner. Due to staff working remotely, contact by phone or email in the first instance is advised.

If you are unhappy with a primary care service, such as your GP practice or pharmacist, you can complain either directly to the Practice Manager of the practice or if you prefer to NHS England, the organisation which manages complaints for these services:

Telephone: 0300 311 22 33 (Monday to Friday 8am to 6pm, excluding bank holidays)

Email: england.contactus@nhs.net

Write to: NHS England, PO Box 16738, Redditch B97 9PT

In the event that something does go wrong and you are harmed during the research and this is due to someone's negligence then you may have grounds for legal action for compensation against the University of Cambridge or the NHS or an individual through their professional indemnity (if appropriate) but you may have to pay your legal costs. The normal National Health Service complaints mechanisms will still be available to you (if appropriate). The University of Cambridge has arranged insurance in case something goes wrong and you are harmed but it is not due to anybody's fault (no-fault compensation).

How will I find out the results of the trial?

At the end of this trial the results will be available to read on our trial website – please see our contact details in part 4 for the link. If you would like us to send you a copy of the results please get in touch with us.

For peer review only - http://bmjopen.bmj.com/site/about/guidelines.xhtml

Part 3: Data Confidentiality

How will information about me be kept confidential?

Your personal details will be collected from your GP practice including your name, address, contact details, date of birth, NHS number – "personal data". We will also collect information about you during your participation in the trial, some relating to your health from various sources – "trial data".

Your trial data will not include your personal data, and will be stored separately using a unique trial identification number.

All information about you (including your personal data) will be stored securely with access restricted to authorised members of the research team from the University of Cambridge and the University of Oxford. Only these people will access your personal data as they need to manage your participation in the trial, collect information from your medical and health records, or audit the data collection process. In addition, authorised staff who work for or with the sponsors of the trial or relevant regulatory authorities may require access to your personal data, your trial data and/or your medical records. This would be to check the accuracy of the trial data and ensure that it is being conducted in accordance with the relevant regulations. All information will be treated in the strictest confidence during that review process.

We will inform your GP that you are taking part in the trial.

Information from medical and health records

The research team will collect information from your GP practice medical record. Usually your practice will pass this information securely to the research team. Sometimes your GP practice will allow the research team to access your medical records directly, or use a contracted third party. This is only done with the appropriate security checks and confidentiality agreements in place.

The research team will also collect information from other centrally held health-related records. The records we will use are Hospital Episodes Statistics data, civil registration mortality data (both held by the appropriate governing body, currently NHS Digital), the Sentinel Stroke National Audit Programme (SSNAP) database, and the Myocardial Ischaemia National Audit Project (MINAP)

For peer review only

For peer review only - http://bmjopen.bmj.com/site/about/guidelines.xhtml

database. To link this information the research team need to send identifying information such as your name, address, NHS number and date of birth to NHS Digital, SSNAP and MINAP so they can identify your health records correctly. It is possible that in the future we may need to link to another health record or registry that we consider to be relevant to the purposes of the research. We will use central NHS records to provide us with your current GP practice and your address, so that we can continue to collect follow-up information should you move. We will continue to collect health record data in the event of your death during the trial and follow-up period.

Only information relevant to the purpose of the trial or understanding how to screen for atrial fibrillation will be collected. We might use this information to decide whether to invite you to take part in other related research studies.

General Data Protection Regulation

The University of Cambridge and NHS Cambridgeshire and Peterborough CCG are the co-sponsors for this trial based in the UK. The University of Cambridge will be using information from you and your medical records in order to undertake this trial and will act as the data controller for this trial. This means that we are responsible for looking after your information and using it properly. The SAFER Trial is part of an important long-term programme of research that relies on long-term follow-up of participants. We will retain your personal and trial data indefinitely to meet the purposes of medical research and any legal, accounting or reporting requirements.

Your rights to access, change or move the information that we hold are limited, as we need to manage it in specific ways in order for the research to be reliable and accurate. To safeguard your rights, we will use the minimum personally identifiable information possible.

You can find out more about how we use your information here https://www.medschl.cam.ac.uk/research/privacy-notice-how-we-use-your-research-data/

We will share your information with collaborating research organisations working with us (both internal and external to this trial) and commercial partners. Only trial data that cannot identify you will be shared. This will be governed by appropriate agreements. Some organisations may be outside of the European Economic Area, where data security regulations may be less stringent than those in the UK. The transfer of information will be done securely and in accordance with local security policies and the Data Protection Act 2018.

For peer review only - http://bmjopen.bmj.com/site/about/guidelines.xhtml

During the trial we will not be able to monitor your ability to consent to continued participation. We will keep any data already collected about you and will continue to collect data from your medical and other health records, in accordance with the consent that you granted at the start of the trial. If, however, we find out as a result of arranging your screening or through your involvement in optional interviews that you are unwell such that you are no longer able to consent to continued participation, we will withdraw you from the trial, while keeping any data about you that we have already collected.

Future updates relating to data confidentiality will be posted on the trial website https://www.safer.phpc.cam.ac.uk/. It is recommended that you visit the website regularly to keep up to date with the progress of the trial and data confidentiality information.

For peer review only

For peer review only - http://bmjopen.bmj.com/site/about/guidelines.xhtml

Part 4: Contact Us

Who do I contact if I have any questions?

Please get in touch with us - our details are below. You can also visit the trial website: https://www.safer.phpc.cam.ac.uk/ for more information.

The trial is registered with the ISRCTN https://www.isrctn.com/ISRCTN72104369.

Contact details:

If you or someone on your behalf needs to contact the research team you can do so as follows:

Phone: contact us using the following number during working hours (Monday to Friday 9am – 5pm): **01223 763491**. If we miss your call or if you call outside these hours, there is an answer phone on this number. If you leave a message we will respond to you at the earliest opportunity.

Email: safer@medschl.cam.ac.uk

Address:
The SAFER Trial
University of Cambridge
Primary Care Unit
Strangeways Research Laboratory
2 Worts Causeway
Cambridge
CB1 8RN

Website: https://www.safer.phpc.cam.ac.uk/

For peer review only

For peer review only - http://bmjopen.bmj.com/site/about/guidelines.xhtml

For peer review only

For peer review only - http://bmjopen.bmj.com/site/about/guidelines.xhtml

Participant ID: <<participant ID>> / barcode

SAFER Trial

Consent Form

Version 1.1 11-12-2020

Please complete and return this form only if you wish to join the SAFER Trial

Title: The SAFER Trial – Screening for Atrial Fibrillation with ECG to Reduce stroke

Chief Investigator: Professor Jonathan Mant, University of Cambridge

IRAS project ID: 272184

Ethics Reference number: 19/LO/1597 **Participant ID**: <<participant ID>> / barcode

If you are willing to take part in the SAFER Trial, please read the following statements and if you agree, sign and date overleaf.

1	I have read and understood the Participant Information Sheet version XX, dated DATE (NAME) for the above trial. I have had the opportunity to ask questions and I am satisfied with the answers and explanations provided.
2	I understand that my participation in this trial is voluntary and that I am free to withdraw at any time, without giving a reason and without my medical care or legal rights being affected.
3	I understand that information from my medical records will be available to the research team as part of the trial.
4	I consent to my trial data being linked to Hospital Episodes Statistics (HES), civil registration mortality data, Sentinel Stroke National Audit Programme (SSNAP) and Myocardial Ischaemia National Audit Project (MINAP). This may involve sharing my personal data with these bodies. I understand that information held and managed by NHS Digital and the registries may be used in order to provide information about my health status (including after my death), my GP practice and my address (should I move). I understand that these details will be used for research purposes only. It is possible that in the future the research team may need to link to another health record or registry not listed that they consider to be relevant to the purposes of the research and I agree to this.
5	I understand that sections of my medical notes or information related directly to my participation in this trial may be looked at by responsible individuals from the sponsors, regulatory authorities and research personnel where it is relevant to my taking part in this research. I give permission for these individuals to have access to my records.
6	I understand that my GP will be informed of my participation in this trial.
7	I understand that my unidentifiable trial data will be shared with other researchers, both internal and external to this trial, and with commercial partners. These parties may be outside the European Economic Area.
8	I understand that I may be contacted about future, related research studies, and that I am under no obligation to take part.
9	I agree to participate in this trial.

For peer review only

[TO BE PRINTED ON TRIAL HEADED PAPER]

Participant ID: <<participant ID>> / barcode

By signing this form you are consenting that you agree with all of the statements listed, and that the details listed below are correct.

Name of participant	Signature	Date

Please check the details below and amend/complete accordingly, then return this form to the trial team using the Freepost envelope enclosed. Alternatively you can complete this consent form online – please see the covering letter enclosed for instructions. As the trial will be conducted remotely, it will be helpful if you could please supply a phone number. If you also have an email address and are happy to provide this, please do so.

Title:	
First name:	
Surname:	
Date of birth (dd/mm/yyyy):	
Gender (M/F/Mx):	
Address:	
Postcode:	
Home Tel.:	
Mobile no.:	
Email:	
NHS no:	
GP Practice name:<br>**Please note:** if this is **not** your current practice and you have recently moved practice, you will not be able to take part at this point. It is possible that your new practice may take part in the future.	

The trial team will return a copy of this consent form to your GP practice for their records. If you would like a copy of your completed consent form please contact the trial team.

The trial team will only use these details in order to contact you for the purposes stated.

1x copy to be retained by the research team; 1x copy to be sent to the participant's GP practice.

For peer review only

For peer review only - http://bmjopen.bmj.com/site/about/guidelines.xhtml

SPIRIT
STANDARD PROTOCOL ITEMS: RECOMMENDATIONS FOR INTERVENTIONAL TRIALS

SPIRIT 2013 Checklist: Recommended items to address in a clinical trial protocol and related documents*

Section/item	Item No.	Description	Page
**Administrative information**			
Title	1	Descriptive title identifying the study design, population, interventions, and, if applicable, trial acronym	1
Trial registration	2a	Trial identifier and registry name. If not yet registered, name of intended registry	4
	2b	All items from the World Health Organization Trial Registration Data Set	Throughout
Protocol version	3	Date and version identifier	N/A
Funding	4	Sources and types of financial, material, and other support	19-20
Roles and responsibilitie s	5a	Names, affiliations, and roles of protocol contributors	1-3
	5b	Name and contact information for the trial sponsor	18
	5c	Role of study sponsor and funders, if any, in study design; collection, management, analysis, and interpretation of data; writing of the report; and the decision to submit the report for publication, including whether they will have ultimate authority over any of these activities	20
	5d	Composition, roles, and responsibilities of the coordinating centre, steering committee, endpoint adjudication committee, data management team, and other individuals or groups overseeing the trial, if applicable (see Item 21a for data monitoring committee)	18
**Introduction**			
Background and rationale	6a	Description of research question and justification for undertaking the trial, including summary of relevant studies (published and unpublished) examining benefits and harms for each intervention	6-7
	6b	Explanation for choice of comparators	8

For peer review only

Objectives	7	Specific objectives or hypotheses	8
Trial design	8	Description of trial design including type of trial (eg, parallel group, crossover, factorial, single group), allocation ratio, and framework (eg, superiority, equivalence, noninferiority, exploratory)	8
**Methods: Participants, interventions, and outcomes**			
Study setting	9	Description of study settings (eg, community clinic, academic hospital) and list of countries where data will be collected. Reference to where list of study sites can be obtained	8-9
Eligibility criteria	10	Inclusion and exclusion criteria for participants. If applicable, eligibility criteria for study centres and individuals who will perform the interventions (eg, surgeons, psychotherapists)	8-9
Interventions	11a	Interventions for each group with sufficient detail to allow replication, including how and when they will be administered	10-12
	11b	Criteria for discontinuing or modifying allocated interventions for a given trial participant (eg, drug dose change in response to harms, participant request, or improving/worsening disease)	8
	11c	Strategies to improve adherence to intervention protocols, and any procedures for monitoring adherence (eg, drug tablet return, laboratory tests)	9-11
	11d	Relevant concomitant care and interventions that are permitted or prohibited during the trial	8
Outcomes	12	Primary, secondary, and other outcomes, including the specific measurement variable (eg, systolic blood pressure), analysis metric (eg, change from baseline, final value, time to event), method of aggregation (eg, median, proportion), and time point for each outcome. Explanation of the clinical relevance of chosen efficacy and harm outcomes is strongly recommended	6-7, 12-16
Participant timeline	13	Time schedule of enrolment, interventions (including any run-ins and washouts), assessments, and visits for participants. A schematic diagram is highly recommended (see Figure)	11-12, 14-16, fig 3

For peer review only - http://bmjopen.bmj.com/site/about/guidelines.xhtml

Sample size	14	Estimated number of participants needed to achieve study objectives and how it was determined, including clinical and statistical assumptions supporting any sample size calculations	13-14
Recruitment	15	Strategies for achieving adequate participant enrolment to reach target sample size	9-10

Methods: Assignment of interventions (for controlled trials)

Allocation:

Sequence generation	16a	Method of generating the allocation sequence (eg, computer-generated random numbers), and list of any factors for stratification. To reduce predictability of a random sequence, details of any planned restriction (eg, blocking) should be provided in a separate document that is unavailable to those who enrol participants or assign interventions	9-10, 15-16
Allocation concealment mechanism	16b	Mechanism of implementing the allocation sequence (eg, central telephone; sequentially numbered, opaque, sealed envelopes), describing any steps to conceal the sequence until interventions are assigned	9-10, 15-16
Implementation	16c	Who will generate the allocation sequence, who will enrol participants, and who will assign participants to interventions	9-10, 15-16
Blinding (masking)	17a	Who will be blinded after assignment to interventions (eg, trial participants, care providers, outcome assessors, data analysts), and how	9-10, 15-16
	17b	If blinded, circumstances under which unblinding is permissible, and procedure for revealing a participant's allocated intervention during the trial	N/A

Methods: Data collection, management, and analysis

Data collection methods	18a	Plans for assessment and collection of outcome, baseline, and other trial data, including any related processes to promote data quality (eg, duplicate measurements, training of assessors) and a description of study instruments (eg, questionnaires, laboratory tests) along with their reliability and validity, if known. Reference to where data collection forms can be found, if not in the protocol	14-16

	18b	Plans to promote participant retention and complete follow-up, including list of any outcome data to be collected for participants who discontinue or deviate from intervention protocols	11-12, 14-16
Data management	19	Plans for data entry, coding, security, and storage, including any related processes to promote data quality (eg, double data entry; range checks for data values). Reference to where details of data management procedures can be found, if not in the protocol	16
Statistical methods	20a	Statistical methods for analysing primary and secondary outcomes. Reference to where other details of the statistical analysis plan can be found, if not in the protocol	16
	20b	Methods for any additional analyses (eg, subgroup and adjusted analyses)	16
	20c	Definition of analysis population relating to protocol non-adherence (eg, as randomised analysis), and any statistical methods to handle missing data (eg, multiple imputation)	16
**Methods: Monitoring**			
Data monitoring	21a	Composition of data monitoring committee (DMC); summary of its role and reporting structure; statement of whether it is independent from the sponsor and competing interests; and reference to where further details about its charter can be found, if not in the protocol. Alternatively, an explanation of why a DMC is not needed	18
	21b	Description of any interim analyses and stopping guidelines, including who will have access to these interim results and make the final decision to terminate the trial	18
Harms	22	Plans for collecting, assessing, reporting, and managing solicited and spontaneously reported adverse events and other unintended effects of trial interventions or trial conduct	15-16, 18
Auditing	23	Frequency and procedures for auditing trial conduct, if any, and whether the process will be independent from investigators and the sponsor	18
**Ethics and dissemination**			

For peer review only - http://bmjopen.bmj.com/site/about/guidelines.xhtml

Research ethics approval	24	Plans for seeking research ethics committee/institutional review board (REC/IRB) approval	4,18
Protocol amendments	25	Plans for communicating important protocol modifications (eg, changes to eligibility criteria, outcomes, analyses) to relevant parties (eg, investigators, REC/IRBs, trial participants, trial registries, journals, regulators)	18
Consent or assent	26a	Who will obtain informed consent or assent from potential trial participants or authorised surrogates, and how (see Item 32)	9,19
	26b	Additional consent provisions for collection and use of participant data and biological specimens in ancillary studies, if applicable	N/A
Confidentialit y	27	How personal information about potential and enrolled participants will be collected, shared, and maintained in order to protect confidentiality before, during, and after the trial	16
Declaration of interests	28	Financial and other competing interests for principal investigators for the overall trial and each study site	19-20
Access to data	29	Statement of who will have access to the final trial dataset, and disclosure of contractual agreements that limit such access for investigators	16, 19
Ancillary and post-trial care	30	Provisions, if any, for ancillary and post-trial care, and for compensation to those who suffer harm from trial participation	N/A
Disseminatio n policy	31a	Plans for investigators and sponsor to communicate trial results to participants, healthcare professionals, the public, and other relevant groups (eg, via publication, reporting in results databases, or other data sharing arrangements), including any publication restrictions	19
	31b	Authorship eligibility guidelines and any intended use of professional writers	N/A
	31c	Plans, if any, for granting public access to the full protocol, participant-level dataset, and statistical code	19
**Appendices**			
Informed consent materials	32	Model consent form and other related documentation given to participants and authorised surrogates	Appendices A, B, C

For peer review only - http://bmjopen.bmj.com/site/about/guidelines.xhtml

Biological specimens	33	Plans for collection, laboratory evaluation, and storage of biological specimens for genetic or molecular analysis in the current trial and for future use in ancillary studies, if applicable	N/A

*It is strongly recommended that this checklist be read in conjunction with the SPIRIT 2013 Explanation & Elaboration for important clarification on the items. Amendments to the protocol should be tracked and dated. The SPIRIT checklist is copyrighted by the SPIRIT Group under the Creative Commons "Attribution-NonCommercial-NoDerivs 3.0 Unported" license.

For peer review only

BMJ Open

A cluster randomised controlled trial of screening for atrial fibrillation in people aged 70 years and over to reduce stroke: protocol for the pilot study for the SAFER trial

Journal:	*BMJ Open*
Manuscript ID	bmjopen-2022-065066.R1
Article Type:	Protocol
Date Submitted by the Author:	26-Jul-2022
Complete List of Authors:	Williams, Kate; University of Cambridge Primary Care Unit, Strangeways Research Laboratory, Department of Public Health and Primary Care<br>Modi, Rakesh; University of Cambridge Primary Care Unit, Strangeways Research Laboratory, Department of Public Health and Primary Care<br>Dymond, Andrew; University of Cambridge Primary Care Unit, Strangeways Research Laboratory, Department of Public Health and Primary Care<br>Hoare, Sarah; THIS Institute, University of Cambridge<br>Powell, Alison; THIS Institute, University of Cambridge<br>Burt, Jenni; THIS Institute, University of Cambridge<br>Edwards, Duncan; University of Cambridge Primary Care Unit, Strangeways Research Laboratory, Department of Public Health and Primary Care<br>Lund, Jenny; University of Cambridge Primary Care Unit, Strangeways Research Laboratory, Department of Public Health and Primary Care<br>Johnson, Rachel; University of Bristol Medical School<br>Lobban, Trudie; Heart Arrhythmia Alliance<br>`Lown, Mark; University of Southampton School of Primary Care Population Sciences and Medical Education, Primary Care and Population Sciences<br>Sweeting, Michael J.; University of Leicester Department of Health Sciences, Department of Health Sciences, University of Leicester<br>Thom, H; University of Bristol Medical School<br>Kaptoge, Stephen; University of Cambridge Department of Public Health and Primary Care, Cardiovascular Epidemiology Unit, Department of Public Health and Primary Care<br>Fusco, Francesco; University of Cambridge Primary Care Unit, Strangeways Research Laboratory, Department of Public Health and Primary Care<br>Morris, Stephen; University of Cambridge Primary Care Unit, Strangeways Research Laboratory, Department of Public Health and Primary Care<br>Lip, Gregory; Liverpool Centre for Cardiovascular Science, University of Liverpool, Liverpool John Moores University and Liverpool Heart & Chest Hospital, Liverpool; Aalborg University Department of Clinical Medicine<br>Armstrong, Natalie; University of Leicester Department of Health Sciences, Department of Health Sciences, University of Leicester, George Davies Centre<br>Cowie, Martin; Royal Brompton Hospital, Guy's & St Thomas' NHS

	Foundation Trust; Kings College London Faculty of Life Sciences and Medicine; Imperial College London Faculty of Medicine<br>Fitzmaurice, David; University of Warwick Warwick Medical School, Health Sciences<br>Freedman, Ben; The University of Sydney Charles Perkins Centre, ben.freedman@sydney.edu.au<br>Griffin, Simon; University of Cambridge Primary Care Unit, Strangeways Research Laboratory, Department of Public Health and Primary Care; MRC Epidemiology Unit, School of Clinical Medicine, University of Cambridge<br>Sutton, Stephen; University of Cambridge Primary Care Unit, Strangeways Research Laboratory, Department of Public Health and Primary Care<br>Hobbs, Richard; University of Oxford Nuffield Department of Primary Care Health Sciences, Nuffield Department of Primary Care Health Sciences<br>McManus, Richard; University of Oxford Nuffield Department of Primary Care Health Sciences, Nuffield Department of Primary Care Health Sciences<br>Mant, Jonathan; University of Cambridge Primary Care Unit, Strangeways Research Laboratory, Department of Public Health and Primary Care<br>SAFER Authorship Group, The; University of Cambridge Primary Care Unit, Strangeways Research Laboratory, Department of Public Health and Primary Care
**Primary Subject Heading**:	Public health
Secondary Subject Heading:	Public health, General practice / Family practice, Cardiovascular medicine, Health services research
Keywords:	Adult cardiology < CARDIOLOGY, PUBLIC HEALTH, PRIMARY CARE, STROKE MEDICINE

SCHOLARONE™
Manuscripts

A cluster randomised controlled trial of screening for atrial fibrillation in people aged 70 years and over to reduce stroke: protocol for the pilot study for the SAFER trial

Corresponding author

Dr Rakesh N Modi

Primary Care Unit, Department of Public Health and Primary Care, University of Cambridge, Strangeways Research Laboratory, 2 Worts' Causeway, Cambridge, UK, CB1 8RN

rnm30@medschl.cam.ac.uk

Authors

1. Dr Kate Williams, kmw36@medschl.cam.ac.uk[1]
2. Dr Rakesh N Modi, rnm30@medschl.cam.ac.uk[1]
3. Andrew Dymond, ad486@medschl.cam.ac.uk[1]
4. Dr Sarah Hoare, sarah.hoare@thisinstitute.cam.ac.uk[2]
5. Dr Alison Powell, alison.powell@thisinstitute.cam.ac.uk[2]
6. Dr Jenni Burt, jenni.burt@thisinstitute.cam.ac.uk[2]
7. Dr Duncan Edwards, dae31@medschl.cam.ac.uk[1]
8. Dr Jenny Lund, jl897@medschl.cam.ac.uk[1]
9. Dr Rachel Johnson, rachel.johnson@bristol.ac.uk[3]
10. Ms Trudie Lobban, trudie@heartrhythmalliance.org[4]
11. Dr Mark Lown, M.Lown@soton.ac.uk[5]
12. Dr Mike Sweeting, michael.sweeting@le.ac.uk[6]
13. Dr Howard Thom, howard.thom@bristol.ac.uk[3]

For peer review only - http://bmjopen.bmj.com/site/about/guidelines.xhtml

14. Dr Stephen Kaptoge, skk22@medschl.cam.ac.uk[7]

15. Dr Francesco Fusco, ff346@medschl.cam.ac.uk[1]

16. Professor Stephen Morris, sm2428@medschl.cam.ac.uk[1]

17. Professor Gregory Y.H. Lip, gregory.lip@liverpool.ac.uk[8]

18. Professor Natalie Armstrong, natalie.armstrong@le.ac.uk[6]

19. Professor Martin R Cowie, martin.cowie@kcl.ac.uk[9]

20. Professor David Fitzmaurice, D.Fitzmaurice@warwick.ac.uk[10]

21. Professor Ben Freedman, ben.freedman@sydney.edu.au[11]

22. Professor Simon J Griffin, profgp@medschl.cam.ac.uk[1, 12]

23. Professor Stephen Sutton, srs34@medschl.cam.ac.uk[1]

24. Professor F.D. Richard Hobbs, richard.hobbs@phc.ox.ac.uk[13]

25. Professor Richard J McManus, richard.mcmanus@phc.ox.ac.uk[13]

26. Professor Jonathan Mant, jm677@medschl.cam.ac.uk[1]

27. The SAFER author group

1. Primary Care Unit, Department of Public Health and Primary Care, University of Cambridge, Strangeways Research Laboratory, 2 Worts' Causeway, Cambridge, UK, CB1 8RN
2. THIS Institute (The Healthcare Improvement Studies Institute), University of Cambridge, Clifford Allbutt Building, Cambridge Biomedical Campus, Cambridge, UK, CB2 0AH
3. Bristol Medical School, University of Bristol, Canynge Hall, 39 Whatley Road, Bristol, UK, BS8 2PS
4. Heart Arrhythmia Alliance, Unit 6B, Essex House, Cromwell Business Park, Chipping Norton, UK, OX7 5SR
5. Primary Care and Population Sciences, Faculty of Medicine, University of Southampton, Aldermoor Health Centre, Aldermoor Close, Southampton, UK, SO16 5ST
6. Department of Health Sciences, University of Leicester, George Davies Centre, University Road, Leicester, UK, LE1 7RH
7. Cardiovascular Epidemiology Unit, Department of Public Health and Primary Care, University of Cambridge, East Forvie Building, Cambridge Biomedical Campus, Cambridge, UK, CB2 0SR
8. Liverpool Centre for Cardiovascular Science, University of Liverpool, Liverpool John Moores University and Liverpool Heart & Chest Hospital, Liverpool, UK; Department of Clinical Medicine, Aalborg University, Denmark.
9. Royal Brompton Hospital, Guy's & St Thomas' NHS Foundation Trust, Sydney Street, London SW3 6NP; Faculty of Life Sciences & Medicine, King's College London, London,

UK; Faculty of Medicine, National Heart & Lung Institute, Imperial College London, London, UK

10. Health Sciences, Warwick Medical School, University of Warwick, Coventry, UK, CV4 7AL
11. Heart Research Institute, University of Sydney, Room 3114, Level 3 East, D17 - Charles Perkins Centre, NSW, Australia, 2006
12. MRC Epidemiology Unit, School of Clinical Medicine, University of Cambridge, Cambridge Biomedical Campus, Cambridge, UK, CB2 0SL
13. Nuffield Department of Primary Care Health Sciences, University of Oxford, Radcliffe Observatory Quarter, Woodstock Road, Oxford, UK, OX2 6GG

For peer review only

For peer review only - http://bmjopen.bmj.com/site/about/guidelines.xhtml

ABSTRACT

Introduction

Atrial fibrillation (AF) is a common arrhythmia associated with 30% of strokes, as well as other cardiovascular disease, dementia and death. AF meets many criteria for screening, but there is limited evidence that AF screening reduces stroke. Consequently, no countries recommend national screening programmes for AF. The Screening for Atrial Fibrillation with ECG to Reduce stroke (SAFER) trial aims to determine whether screening for AF is effective at reducing risk of stroke. The aim of the pilot study is to assess feasibility of the main trial and inform implementation of screening and trial procedures.

Methods and analysis

SAFER is planned to be a pragmatic randomised controlled trial (RCT) of over 100,000 participants aged 70 years and over, not on long term anticoagulation therapy at baseline, with an average follow-up of five years. Participants are asked to record four traces every day for three weeks on a hand-held single-lead ECG device. Cardiologists remotely confirm episodes of AF identified by the device algorithm, and general practitioners follow-up with anticoagulation as appropriate. The pilot study is a cluster RCT in 36 UK general practices, randomised 2:1 control to intervention, recruiting approximately 12,600 participants. Pilot study outcomes include AF detection rate, anticoagulation uptake, and other parameters to incorporate into sample size calculations for the main trial. Questionnaires sent to a sample of participants will assess impact of screening on psychological health. Process evaluation and qualitative studies will underpin implementation of screening during the main trial. An economic evaluation using the pilot data will confirm whether it is plausible that screening might be cost-effective.

Ethics and dissemination

The London – Central Research Ethics Committee (19/LO/1597) and Confidentiality Advisory Group (19/CAG/0226) provided ethical approval. Dissemination will be via publications, patient-friendly summaries, reports and engagement with the UK National Screening Committee.

Trial registration number: ISRCTN72104369

KEYWORDS

Atrial fibrillation; screening; randomised controlled trial; primary care; stroke prevention

For peer review only

Word count: 3653 (excluding figures/tables/box and acknowledgements onwards)

For peer review only

For peer review only - http://bmjopen.bmj.com/site/about/guidelines.xhtml

ARTICLE SUMMARY

Strengths and limitations of this study

- SAFER is a large multicentre pragmatic randomised controlled trial (RCT) planned to be the largest trial of atrial fibrillation (AF) screening that has been performed.
- This internal pilot study will have good external validity, providing data on parameters for an AF screening programme in real-world conditions.
- The process evaluation of the pilot study will inform the implementation of a large-scale AF screening programme.
- Participant recruitment prior to cluster randomisation will ensure that intervention and control participants are similar, and are likely to take up screening if offered it.
- Despite the fact that anticoagulation is indicated in some people under 70, the SAFER trial is not screening in this age group.

INTRODUCTION

Atrial fibrillation (AF) is a cardiac arrhythmia present in approximately 10% of people aged over 65 years.[1] AF is increasing in prevalence,[2] and is associated with a five-fold increase in the risk of stroke,[3] as well as other negative health outcomes (such as heart failure, dementia and death).[4-8] Whilst 30% of strokes are associated with AF, 10% of strokes occur in people unaware that they have AF because it can be asymptomatic, intermittent ('paroxysmal AF') and/or undiagnosed.[9-12] AF-related strokes tend to be more severe than strokes due to other causes, imposing burdens on patient, family, and health and social care systems. [10 13]

AF is diagnosed on an electrocardiogram (ECG).[14 15] This has traditionally been achieved by a health professional interpreting a 12-lead ECG. However, 30 seconds on a single-lead ECG is now regarded as sufficient to diagnose AF.[16-20] Furthermore, acceptable and accessible portable technologies such as wearable patches, smart watches, and hand-held devices are available that can test for AF repeatedly over longer periods of time. [21 22] These technologies are sensitive to AF,[23] and can detect paroxysmal AF.[21 24]

Treatment with oral anticoagulation can effectively[25-27] and cost-effectively[28 29] reduce risk of stroke associated with AF, especially when part of an integrated care or holistic approach to AF management, as advocated in guidelines.[30 31] However, a sizeable minority of eligible patients are not taking anticoagulants.[21 32-36] With non-Vitamin-K antagonist oral anticoagulants (NOACs; also

For peer review only - http://bmjopen.bmj.com/site/about/guidelines.xhtml

called direct oral anticoagulants, DOACs) that require substantially less monitoring, and stronger recommendations for anticoagulation in clinical guidelines,[19 31] the rates of anticoagulation are increasing, but remain suboptimal.[37-39]

Undiagnosed AF is common and can be detected with simple and portable technology, and there are effective treatments available.[18 40-42] AF screening therefore fulfils many of the criteria for initiating a national systematic screening programme.[21 40 43 44]

However, no countries endorse national AF screening programmes.[14 31 33 45] Until recently, there was no evidence from randomised controlled trials of the impact of AF screening on stroke and mortality.[33] Two trials of different approaches to AF screening published in 2021 showed promising, but inconclusive results.[46-49] Both recruited much smaller numbers than is planned for SAFER (approximately 28000 for STROKESTOP and 6000 for LOOP).[46-49] Neither showed a reduction in ischaemic stroke associated with screening although STROKESTOP reported a reduction in a composite endpoint (ischaemic or haemorrhagic stroke, systemic embolism, death, and hospitalisation for bleeding). As a result in early 2022 the US Preventive Services Task Force (USPSTF) did not change its previous recommendation that there was insufficient evidence to determine whether there was greater benefit than harm for ECG screening for AF. Thus, evidence is required from a much larger randomised trial to inform guidelines and national screening body recommendations, a gap that SAFER is intended to fill.

The SAFER trial is a large, pragmatic, open-label, primary care-based RCT which will recruit around 100,000 participants and assess whether screening for AF is effective and cost-effective at reducing stroke and other outcomes.[50] It will randomise participants after consent and will investigate ways to improve implementation of screening. It will use intermittent monitoring via hand-held ECGs which will detect higher-burden AF associated with higher clinical risk than continuous monitoring.[48] It will examine harms as well as benefits of an AF screening programme.[45]

The internal pilot study detailed in this protocol is a cluster RCT recruiting participants who will be followed up during the main trial. The objectives of the internal pilot study are to assess intermediate outcomes such as AF detection rate and anticoagulation rate, reduce uncertainty concerning key parameters for the design, conduct and sample size calculations for the main trial, examine the psychological impact of screening, and investigate ways to optimise the delivery of the AF screening intervention.

For peer review only - http://bmjopen.bmj.com/site/about/guidelines.xhtml

For peer review only

METHODS AND ANALYSIS

Aim

To inform a decision to proceed to the main trial taking account of key intermediate outcomes (AF detection rate; anticoagulation uptake in screen detected AF), an economic analysis, and a revised sample size calculation. Also to assess any psychological impact of screening, and draw lessons for how best to implement screening in the main trial.

Design

A pragmatic, primary care-based, multi-centre, two-parallel arm, open-label, practice-level cluster RCT which aims to recruit 12,600 participants from 36 practices in a 2:1 ratio of usual care (control) to screening (intervention). Participants will be followed up for 12 months for pilot study outcomes, and also for an average of five years for main trial outcomes. There will be an embedded process evaluation and qualitative studies, and an economic evaluation. The first practice was randomised on 16th April 2021. Follow up (for the internal pilot) is scheduled to finish on 30th May 2023.

Participants and setting

Participating practices will be drawn from a range of UK urban and rural settings, serving patients with a variety of different health and social needs. The vast majority of the UK population is registered with a practice that provides most AF care with referral to secondary care only for more complex cases.[19]

Eligibility

Participants

Broad eligibility criteria have been employed to maximise eternal validity (**Table 1**).

Table 1. Eligibility criteria for participants in the SAFER pilot and main trial

Inclusion criteria	Exclusion criteria (as coded on the primary care health record)
Participant has given valid informed consent	On long-term anticoagulation therapy
Aged 70 years or older	On the practice palliative care register
	Resident in a nursing or care or residential home

For peer review only - http://bmjopen.bmj.com/site/about/guidelines.xhtml

	Consented to another trial that will affect participation in SAFER
	Non-UK resident

According to guidelines, the vast majority of people aged 70 years or older with AF should be offered anticoagulation.[19] Participants with an existing diagnosis of AF on the practice electronic AF register (which includes both paroxysmal and persistent AF) but who are not being prescribed anticoagulation are included because screening these participants for AF may encourage anticoagulation use.[42 46 51]

Patients coded as resident in a nursing / care / residential home in the electronic search of patient records will be excluded due to practical difficulties.

Patients taking part in another trial will be excluded if participation in both trials could compromise either trial or affect patients' safety.

Recruitment

Practices

Practice recruitment will be managed by the National Institute for Health and Care Research (NIHR) Clinical Research Network (CRN) – a national network that coordinates and supports research delivery. The CRN will approach practices with information about the trial. Practices will express interest via an online form.

Participants

The practice will send approximately 1,200 randomly selected eligible patients an invitation pack consisting of a participant information sheet (PIS), consent form and Freepost envelope (see **Appendix A to C**). In initial practices a negative reply slip will be included in the pack so that reasons for non-participation can be analysed. The exact number invited will vary between practices to achieve recruitment targets based on their characteristics and any associations with recruitment (e.g. more invitations to people in more deprived areas).

To facilitate convenience, participants will have the option to return the consent form in a Freepost envelope, or to provide consent online. Reminder invitations, emails, Short Message Service (SMS), and/or invitation of additional eligible patients may be utilised if response rates are poor.

For peer review only - http://bmjopen.bmj.com/site/about/guidelines.xhtml

Randomisation and allocation

On the day after the recruited and consented participant target number is reached for a practice (350 participants), we will close recruitment and the practice will be randomised, stratified by practice location deprivation score[52] and prevalence of AF reported in the Quality and Outcomes Framework. No recruitment will take place in a practice once randomisation has occurred.

Randomisation will be implemented using a secure online randomisation system (Sortition®[53]) hosted by the University of Oxford Clinical Trials Unit (CTU). Practices will be randomised using random permuted blocks within 9 strata corresponding to 3 groups (tertiles) of practice location deprivation score and 3 groups (tertiles) of practice-level prevalence of AF. The block sizes will be known only to the trial statistician and the randomisation system programmer. All activity on the programme will have an audit trail.

Blinding of allocation to the trial team and to the practices will not be possible.

Intervention development

The screening intervention was developed with a range of stakeholders that included patient associations, patients, screening policy makers, GPs, and researchers. The intervention was tested in a feasibility study in 10 practices, which demonstrated that the intervention was feasible and acceptable to participants and practice staff.[54] In this feasibility study, practice staff conducted screening consultations in which participants were instructed how to use the ECG device. In response to the Covid-19 pandemic, a second feasibility study was undertaken in three practices, which showed that a 'remote model' of delivery of the intervention was feasible: participants could be instructed on how to use the ECG device through written instructions and video, and with optional telephone support from the study administrative team. This model ensured a low risk of Covid-19 transmission and reduced workload for primary care. Training of practices was also successfully delivered remotely. This included training on how to manage and discuss results with participants and online anticoagulation training to manage participants in line with current guidelines.[19 55]

The final intervention model is summarised in the logic model in **Figure 1**.

For peer review only - http://bmjopen.bmj.com/site/about/guidelines.xhtml

<<Figure 1. Logic model of the intervention in the SAFER trial>>

Screening intervention

Participants in intervention practices will receive an invitation to screening. Those who accept this will receive a call from the study team to arrange home delivery of the single-lead ECG device and written/video instructions, and to offer a subsequent screening consultation if required to provide support. In this consultation, the participants will be guided on use of the device and with the help of test ECG traces, how to produce a trace of acceptable quality.

Participants will undertake three weeks of intermittent screening (four 30 second traces each day) as well as when experiencing symptoms (e.g. palpitation, dizziness) using the portable Zenicor device (www.zenicor.com). They will transmit their ECG recordings via mobile network to a remote database by pressing a button on the device. If no traces have been received within 10 days, or if more than 25% of traces recorded on days 4 to 10 are tagged by the algorithm as low quality, the trial team will contact the participant to offer further support. We stress to participants both in information sheets and verbally (during the device delivery call) that the ECG device provided should not be used by anyone else.

Participants will be provided with a freepost envelope and asked to return the Zenicor device to the trial team at the end of the screening period.

Practices in the intervention arm are given on-line training on the NICE AF guidelines.[19]

Zenicor device

The screening device being used is the Zenicor hand-held single-lead ECG device. This device is usable in any location, allows repeated ECGs, and can store and transmit multiple ECG traces to a central system for analysis.[21 24] Photoplethysmography[56] and blood-pressure machines[57] have not proved accurate enough, and stakeholder discussion deemed patches less practical. The diagnostic model of the Zenicor device, its associated diagnostic algorithms, and subsequent cardiologist review have been used successfully at scale in the STROKESTOP AF screening trial in over 7000 participants.[42 58] The algorithm for detecting AF showed a sensitivity of 98% and specificity of 88%.[59] A photograph of the Zenicor device is shown in **Figure 2**.

<<Figure 2. Zenicor hand-held ECG device used to screen for AF in the SAFER trial>>

For peer review only - http://bmjopen.bmj.com/site/about/guidelines.xhtml

Screening results

A proprietary algorithm will analyse the ECG traces and place a digital flag on ECGs that might show AF. These will be reviewed by a cardiologist or cardiac technician who will determine whether AF or any other important rhythm disturbance is present. If there is uncertainty, the trace will be reviewed by another cardiologist. A confirmatory 12-lead ECG is not required.[60] The cardiologists will create a report with recommendations for the GP. Possible results are shown in **Table 2**.

Table 2 Categories of screening results as reported to general practices in the SAFER trial

Diagnosis	Definition
**1. AF ≥ 30 seconds**	AF is observed for a continuous period of 30 seconds. Sufficient readable beats (i.e. disregarding poor quality sections of an ECG) show AF
**2. Cannot exclude AF ≥ 30 seconds**	Indeterminate result – usually due to poor quality ECG traces
**3. Other significant arrhythmia**	This may include, but is not limited to:<br>➢ 2nd/3rd degree heart block<br>➢ Ventricular tachycardia<br>➢ Supraventricular tachycardia<br>➢ Any other significant arrhythmia
**4. No AF ≥ 30 seconds detected**	This will include, but is not limited to:<br>➢ Sinus rhythm<br>➢ AF < 30 seconds<br>➢ Bradycardia<br>➢ Ectopic beats
**5. Screening failure**	Unable to record any ECGs of sufficient quality for review

The trial team will send the screening results to the practice, including copies of relevant ECG traces for positive (AF or other) diagnoses. The GP can access ECG traces and reports for all of their patients freely via the Zenicor web-based system. Practice staff will notify participants of their screening result.

For results 1-3 (table 2), the practices will offer participants a consultation to discuss the result and its appropriate management. GPs are not provided with data on burden of AF, so this will not be considered. See **Figure 3** for a trial schematic. Practices are monitored to ensure that all patients who are found to have AF are reviewed by their GP.

<<Figure 3. SAFER Trial schematic>>

For peer review only - http://bmjopen.bmj.com/site/about/guidelines.xhtml

It is not possible to report results in 'real time'. If participants experience any symptoms, they are advised to seek medical help in the way they usually would, and not wait for the results of the screening (see Appendix D, Screening Information Leaflet).

Control practices

These will provide usual care, which might involve opportunistic screening.

Outcomes

Primary and secondary outcomes are shown in **Box 1**. The internal pilot will specifically report on outcomes that are relevant for consideration of continuation of the trial. Participants in the internal pilot study will also be followed-up for an average of five years for main trial outcomes. The process evaluation during the pilot (protocol to be published separately) will report outcomes to guide the successful delivery of the SAFER main trial and a national-scale AF screening programme.

A random sample of participants stratified by age and sex in both intervention and control arms will be sent questionnaires to assess possible psychological effects of screening. Qualitative work will also contribute to understanding the benefits and harms of screening, and participant experience.

Box 1. Primary and secondary outcomes assessed in the SAFER internal pilot study

For peer review only

For peer review only - http://bmjopen.bmj.com/site/about/guidelines.xhtml

Primary outcome:

- Atrial fibrillation
  - In intervention practices: the number of participants that had AF detected through screening.
  - In intervention and control practices: the number of newly detected AF patients in intervention practices compared with control practices.

Secondary outcomes:

- Uptake of anticoagulation
  - Proportion of participants with AF detected through screening in intervention practices who were started on anticoagulation.
  - Number of participants with newly detected AF that were started on anticoagulation in intervention and control practices.

- Parameters to refine the sample size calculation for the main trial (current assumptions in parentheses)
  - Proportion of consented participants in intervention practices who are screened over the screening period (85%)
  - Proportion of screened patients in whom newly diagnosed AF is detected (3%)
  - Proportion of participants with newly diagnosed AF from screening who commence anticoagulation (80%)
  - Proportion of participants with a known diagnosis of AF that is detected by screening who newly commence anticoagulation, despite previously not being prescribed anticoagulation (55%)

Our definition of newly detected AF is a first AF code recorded within twelve months of randomisation and no AF code in the GP records prior to the date the practice was randomised.

Sample size

Sample size calculations are based on 350 consented participants from each of the 12 intervention and 24 control practices, and the assumption that 85% of participants per intervention practice will be screened. This will provide a 90% power at 5% significance level to detect a 1.1% absolute difference in the frequency of diagnosis of new AF between intervention and control practices, assuming 3% newly diagnosed AF is detected in screened patients[42] and an intraclass correlation coefficient of 0.001.

Sending the heath questionnaire to 1,800 participants will give us 90% power to detect a 4 point difference in the Spielberger questionnaire, assuming 60% respond – the rate achieved in the SAFE trial.[60]

For peer review only - http://bmjopen.bmj.com/site/about/guidelines.xhtml

Data collection

Baseline data collection

Baseline data detailed in **Table 3** will be collected from the GP electronic medical records for all individuals who have consented to participate in the trial.

Table 3. Baseline participant data to be collected for the SAFER pilot study

Category	Variable collected
**Demographics**	Age
	Sex
	Ethnicity
	Index of Multiple Deprivation (IMD) based on participant postcode
**Comorbidities**	Atrial fibrillation
	Stroke or transient ischaemic attack
	Coronary heart disease
	Peripheral arterial disease
	Heart failure
	Hypertension
	Diabetes mellitus
	Stroke or transient ischaemic attack
	Dementia
	Depression
**Clinical scores and indices**	CHA_2DS_2-VASc score
	HAS-BLED score
	ORBIT score
	Frailty index
**Other variables**	Height
	Weight
	Alcohol intake
	Smoking status
	Severe acute respiratory syndrome coronavirus 2 PCR result

Follow-up data

This section excludes outcomes for the main trial, which will be detailed in the main trial protocol.

For peer review only - http://bmjopen.bmj.com/site/about/guidelines.xhtml

Atrial fibrillation:

1. New diagnoses of AF picked up in both intervention and control practices since screening initiation using GP electronic data
2. Positive diagnoses of AF identified by screening (intervention practices only)

Uptake of anticoagulation:

1. For patients diagnosed with AF, whether or not they are prescribed anticoagulation (intervention and control practices) using GP electronic data
2. Initiation of anticoagulation in AF detected through screening programme (intervention practices only)

Process variables:

1. Whether patients agree to screening (intervention practices only)
2. Whether patients are screened (intervention practices only)

Psychological outcomes:

The psychological effects of screening and impact on functional status will be assessed by comparing responses to the Spielberger state anxiety inventory (SAI) short form,[61] EQ5-D-5L[62] and Short-form 8 (SF-8).[63] Changes in responses over time in both groups will be quantified as well as comparisons of responses according to uptake and outcome of screening. These generic measures may be relatively insensitive to some potential specific impacts of screening for atrial fibrillation, but as they do not include reference to the screening programme they enable comparison between screening and control groups. Furthermore, they facilitate comparisons with screening programmes for other conditions, and with other unrelated health service interventions. The questionnaires will be posted to a random (MS Access RND function) sample of participants (126 per intervention practice and 36 per control practice, matched for age (70 to 73; 74 to 77; 78 years and over) and sex in six groups. The target numbers in the intervention arm are raised from our sample size calculation to increase the data available from participants who screen positive. Questionnaires will be posted to the screening group alone at baseline (pre-invitation to screening). Both groups will receive questionnaires after 8 weeks and 6 months.

Data management

For peer review only - http://bmjopen.bmj.com/site/about/guidelines.xhtml

Data sent from practices to the trial team will be labelled with participant ID number (link-anonymisation), initials and partial date of birth. The local investigator at each site is responsible for case report form (CRF) integrity. We will offer secure online data capture (including e-consent), using an established secure system that complies with sponsor security policies (Qualtrics[64]).

ECG traces on the Zenicor system will be labelled with participant ID number, initials and partial date of birth.

Participant questionnaires will be link-anonymised and returned to the trial team by post or online prior to checking and entering.

Participant identifiable data will be stored, handled and processed securely and confidentially, in accordance with sponsor data security policies, UK data laws, and ethical guidelines. Access will be restricted to specific members of the trial team. Further information is accessible on the trial website (https://www.safer.phpc.cam.ac.uk/).

Statistical analysis

Data will be analysed according to CONSORT principles and its extension for cluster trials.[65] Outcomes will be analysed using an intention-to-treat principle for primary analysis. However, as both external and internal validity are important in the pilot study, secondary analysis will be conducted according to the per-protocol principle, when necessary and justified.

The proportion of those consented who took up screening, the proportion found to have AF (both new and previously known), and proportion who were anticoagulated will be calculated. The proportion of diagnoses of new AF participants in intervention and control practices and rate of anticoagulation will be compared. Clustering by practices will be accounted for with an adjusted Chi-square test for simple comparisons and mixed effects regression models for covariates.

Process evaluation and qualitative work

A mixed-methods process evaluation will be conducted to explore how AF screening is delivered and perceived at practice and patient levels. Qualitative work will seek to understand participant experiences of being invited to, and taking part in, the study.

For peer review only - http://bmjopen.bmj.com/site/about/guidelines.xhtml

These will contribute to refining the theory of the intervention, which will help provide recommendations for an acceptable and sustainable screening programme at scale.

Economic analysis

The pilot data will be used to update a published model, composed of a decision tree followed by a Markov model.[23] The purpose of this model is to confirm that it is plausible that screening might be cost-effective using the parameters obtained in this pilot trial. All patients entering the decision tree will incur an invitation cost and the test cost will be applied to those patients who accept screening. Screen-negative patients will not accrue any additional costs and Quality Adjusted Life Years (QALYs). The remaining patients are true positive and, thus, will enter the Markov model. This model will simulate their survival trajectories accounting for their condition and, their lifetime costs and QALYs, which will be discounted at a 3.5% annual rate and half-cycle corrected.

The costs needed to implement the screening programme will be calculated using a micro-costing approach to include all the relevant costs, such as the invitation cost and cost due to the device use (e.g. shipment of the device and the training to use the device).[66] Where needed, the costs incurred by the NHS will be updated using the most recent available data, such as the British National Formulary for the cost of anticoagulant therapies.[67]

The model will be employed to perform a probabilistic analysis and compute the total costs and QALYs. The differences in costs and QALYs between the SAFER intervention and usual care will be calculated and combined to obtain the incremental cost-effectiveness ratio (ICER). Likewise, the expected value of perfect information (EVPI) will be calculated by assuming that the value of one QALY is equal to £20,000, which reflects the cost-effectiveness threshold used by the National Institute for Health and Care Excellence (NICE). Then, the EVPI will be projected to the national level considering the eligible population for the screening and assuming that the screening programme will be provided for the next 10 years.[68]

Management and oversight

The University of Cambridge and NHS Cambridgeshire & Peterborough Clinical Commissioning Group (CCG) are co-sponsors. The trial management group (consisting of the chief investigator and researchers from each group) and the programme steering committee (PSC), which has an independent chair and four independent members, will appraise data and decide on continuation

For peer review only - http://bmjopen.bmj.com/site/about/guidelines.xhtml

and course of the study in consultation with the NIHR. An active risk register has been compiled in consultation with the funder and sponsors, and will be monitored and updated throughout.

Patient and public involvement

The SAFER programme has been guided since inception by patient and public representatives who participate in all-investigator meetings. Trudie Lobban, Chief Executive and Founder of the Atrial Fibrillation Association (AFA), has been involved in the development of the research from the outset as a PPI member. The AFA represents over 64,000 people with atrial fibrillation.

Additional PPI members have been recruited independently of the AFA. Many are in the age range for AF screening; some of them either have AF or have a partner with AF. The PSC has an independent lay member who is a stroke survivor.

The PPI members are consulted throughout the trial on all aspects of the research, including: possible psychological harms of screening; participant-facing documents; how to approach participants; instructing participants on trials and screening procedures; web-based materials and qualitative data-collection material. The AFA will help with dissemination of the findings through its website and members.

ETHICS AND DISSEMINATION

Ethics

Ethical approval

The SAFER pilot trial has received a favourable ethical opinion from the London – Central NHS Research Ethics Committee (19/LO/1597) and the Confidentiality Advisory Group (19/CAG/0226). Modifications of the full protocol are detailed in amendments. Important modifications will be communicated to the sponsors, funder, collaborators, practices, participants, trial registries and disseminators as relevant.

Consent

Participants will be required to provide valid written informed consent, either via post or online. Consented participants from screening practices will be approached with an offer of AF screening.

Dissemination

For peer review only - http://bmjopen.bmj.com/site/about/guidelines.xhtml

The study will generate peer-reviewed publications to disseminate to academics, health professionals, policy-makers, patient organisations and the print and electronic media. After publication, data may be available to others according to data sharing agreements in compliance with the funder and sponsor policies. Summary documents will be made available to participants at the end of the study. PPI groups and media engagement will help disseminate findings. Accessible reports will be generated for national screening committees, commissioners and other decision makers. Funders' reports will be submitted in accordance with their policies.

ACKNOWLEDGEMENTS

We thank the NIHR Clinical Research Network for their support recruiting practices. We also thank the participating GP practices and SAFER participants for their contributions.

COMPETING INTERESTS

JM has performed consultancy work for BMS/Pfizer and Omron. FDRH reports occasional consultancy for BMS/Pfizer, Bayer and BI over the past 5 years. NA is a member of the UK National Screening Committee's Adult Reference Group. MS is a full-time employee of AstraZeneca. MRC reports consultancy for AstraZeneca, Abbott, Medtronic, Bayer, Novartis, Boehringer-Ingelheim-Lilly Alliance, Servier & Pfizer over the past 5 years. RMc's employer the University of Oxford receives consultancy and licencing payments from Omron and Sensyne for BP telemonitoring interventions. GYHL: Consultant and speaker for BMS/Pfizer, Boehringer Ingelheim and Daiichi-Sankyo. No fees are received personally. SJG has received honoraria from Astra Zeneca for lectures at postgraduate educational meetings for primary care teams about type 2 diabetes. BF has received speaker fees, honoraria, and non-financial support from the BMS and Pfizer Alliance; grants to the Institution for investigator-initiated studies from the BMS and Pfizer Alliance; and loan devices for investigator-initiated studies from Alivecor: all were unrelated to the present study but related to screening for AF.

FUNDING

The SAFER pilot study and main trial are funded by the National Institute for Health and Care Research (NIHR) [Programme Grants for Applied Research Programme (Reference Number RP-PG-0217-20007)]. The SAFER feasibility study is funded by the NIHR [School for Primary Care Research (SPCR-2014-10043, project 410)]. SAFER is a contributor to / partner of AFFECT-EU receiving funding from the European Union's Horizon 2020 research and innovation Programme under grant

For peer review only - http://bmjopen.bmj.com/site/about/guidelines.xhtml

agreement NO. 847770. RM and JL are supported by the Wellcome Trust as part of the Wellcome Trust PhD Programme for Primary Care Clinicians [grant number 203921/Z/16/Z]. JB, SH and AP are based in The Healthcare Improvement Studies Institute (THIS Institute), University of Cambridge. THIS Institute is supported by the Health Foundation, an independent charity committed to bringing about better health and healthcare for people in the UK. JB is supported by the Health Foundation's grant to the University of Cambridge for The Healthcare Improvement Studies (THIS) Institute (RG88620). JM and FDRH are NIHR Senior Investigators. FDRH acknowledges support from NIHR ARC OTV and Oxford BRC (OUT). RJM is an NIHR Senior Investigator and acknowledges support from NIHR ARC OTV. NA is supported by a Health Foundation Improvement Science Fellowship and also by the NIHR Applied Research Collaboration East Midlands (ARC EM). RJ is an NIHR-funded Academic Clinical Lecturer. The University of Cambridge has received salary support in respect of SJG from the NHS in the East of England through the Clinical Academic Reserve. BF received funding from the Medical Research Future Fund International Clinical Trial Collaboration Grant to perform SAFER-AUS as part of SAFER, and a NSW Health Senior Researcher Cardiovascular Grant for work in AF. All the funders and sponsors had no involvement in the development of this protocol and will have no involvement in any aspect of the study itself. The views expressed are those of the author(s) and not necessarily those of the NHS, the Wellcome Trust, the NIHR or the UK Department of Health and Social Care.

AUTHORS' CONTRIBUTIONS

JM is the guarantor. RNM drafted the manuscript. KW and AD are coordinating, planning gaining ethical approval, conduct and helping design the study. JM, JB, NA, DE, TL, ML, MS, GL, MC, DF, BF, SG, SS, FRH and RJM undertook design, planning and oversaw conduct of the study. RNM, SH, AP, JB, RJ and NA designed the process evaluation and qualitative studies. JL designed the collection and analysis of some of the pilot outcome data collection. TL is a PPI representative that has informed design, outcomes and dissemination plan. SM, FF and HT designed the economic evaluation and will oversee its conduct. MS and SK designed the statistical analysis and will oversee its conduct. The SAFER author group contributed to planning and design of study, applying for funding, oversaw conduct, and writing of the protocol for the ethical approval. All authors reviewed and had the option to edit the final manuscript.

REFERENCES

1. Fitzmaurice DA, Hobbs FD, Jowett S, et al. Screening versus routine practice in detection of atrial fibrillation in patients aged 65 or over: cluster randomised controlled trial. *Bmj*

For peer review only - http://bmjopen.bmj.com/site/about/guidelines.xhtml

2007;335(7616):383. doi: 10.1136/bmj.39280.660567.55 [published Online First: 2007/08/04]
2. Krijthe BP, Kunst A, Benjamin EJ, et al. Projections on the number of individuals with atrial fibrillation in the European Union, from 2000 to 2060. *Eur Heart J* 2013;34(35):2746-51. doi: 10.1093/eurheartj/eht280 [published Online First: 2013/08/01]
3. Wolf PA, Abbott RD, Kannel WB. Atrial fibrillation as an independent risk factor for stroke: the Framingham Study. *Stroke* 1991;22(8):983-8. doi: 10.1161/01.str.22.8.983 [published Online First: 1991/08/01]
4. Guha K, McDonagh T. Heart failure epidemiology: European perspective. *Current cardiology reviews* 2013;9(2):123-27.
5. Violi F, Soliman EZ, Pignatelli P, et al. Atrial Fibrillation and Myocardial Infarction: A Systematic Review and Appraisal of Pathophysiologic Mechanisms. *Journal of the American Heart Association* 2016;5(5):e003347. doi: doi:10.1161/JAHA.116.003347
6. Singh-Manoux A, Fayosse A, Sabia S, et al. Atrial fibrillation as a risk factor for cognitive decline and dementia. *European Heart Journal* 2017;38(34):2612-18.
7. Benjamin EJ, Wolf PA, D'Agostino RB, et al. Impact of atrial fibrillation on the risk of death: the Framingham Heart Study. *Circulation* 1998;98(10):946-52.
8. Burdett P, Lip GYH. Atrial fibrillation in the UK: predicting costs of an emerging epidemic recognizing and forecasting the cost drivers of atrial fibrillation-related costs. *European Heart Journal - Quality of Care and Clinical Outcomes* 2020 doi: 10.1093/ehjqcco/qcaa093
9. Perera KS, Vanassche T, Bosch J, et al. Global Survey of the Frequency of Atrial Fibrillation-Associated Stroke: Embolic Stroke of Undetermined Source Global Registry. *Stroke* 2016;47(9):2197-202. doi: 10.1161/strokeaha.116.013378 [published Online First: 2016/08/11]
10. Hannon N, Daly L, Murphy S, et al. Acute hospital, community, and indirect costs of stroke associated with atrial fibrillation: population-based study. *Stroke* 2014;45(12):3670-74.
11. Xiong Q, Proietti M, Senoo K, et al. Asymptomatic versus symptomatic atrial fibrillation: A systematic review of age/gender differences and cardiovascular outcomes. *Int J Cardiol* 2015;191:172-7. doi: 10.1016/j.ijcard.2015.05.011 [published Online First: 2015/05/15]
12. Wallenhorst C, Martinez C, Freedman B. Risk of Ischemic Stroke in Asymptomatic Atrial Fibrillation Incidentally Detected in Primary Care Compared with Other Clinical Presentations. *Thromb Haemost* 2021(EFirst) [published Online First: 30.06.2021]
13. Stewart S, Murphy NF, Walker A, et al. Cost of an emerging epidemic: an economic analysis of atrial fibrillation in the UK. *Heart* 2004;90(3):286-92. doi: 10.1136/hrt.2002.008748 [published Online First: 2004/02/18]
14. Mairesse GH, Moran P, Van Gelder IC, et al. Screening for atrial fibrillation: a European Heart Rhythm Association (EHRA) consensus document endorsed by the Heart Rhythm Society (HRS), Asia Pacific Heart Rhythm Society (APHRS), and Sociedad Latinoamericana de Estimulación Cardíaca y Electrofisiología (SOLAECE). *EP Europace* 2017;19(10):1589-623. doi: 10.1093/europace/eux177
15. Calkins H, Hindricks G, Cappato R, et al. 2017 HRS/EHRA/ECAS/APHRS/SOLAECE expert consensus statement on catheter and surgical ablation of atrial fibrillation. *Heart rhythm* 2017;14(10):e275-e444. doi: 10.1016/j.hrthm.2017.05.012 [published Online First: 2017/05/17]
16. Mairesse GH, Moran P, Van Gelder IC, et al. Screening for atrial fibrillation: a European Heart Rhythm Association (EHRA) consensus document endorsed by the Heart Rhythm Society (HRS), Asia Pacific Heart Rhythm Society (APHRS), and Sociedad Latinoamericana de Estimulacion Cardiaca y Electrofisiologia (SOLAECE). *Europace* 2017;19(10):1589-623. doi: 10.1093/europace/eux177 [published Online First: 2017/10/20]

For peer review only - http://bmjopen.bmj.com/site/about/guidelines.xhtml

17. Freedman B, Camm J, Calkins H, et al. Screening for Atrial Fibrillation: A Report of the AF-SCREEN International Collaboration. *Circulation* 2017;135(19):1851-67. doi: 10.1161/circulationaha.116.026693 [published Online First: 2017/05/10]
18. Kirchhof P, Benussi S, Kotecha D, et al. 2016 ESC Guidelines for the management of atrial fibrillation developed in collaboration with EACTS. *Eur Heart J* 2016;37(38):2893-962. doi: 10.1093/eurheartj/ehw210 [published Online First: 2016/08/28]
19. National Institute for Health and Care Excellence. Atrial fibrillation: diagnosis and management (ng196): National Institute of Health and Care Excellence, 2021.
20. Sun W, Freedman B, Martinez C, et al. Atrial Fibrillation Detected by Single Time-Point Handheld Electrocardiogram Screening and the Risk of Ischemic Stroke. *Thromb Haemost* 2021(EFirst) [published Online First: 16.08.2021]
21. Jones NR, Taylor CJ, Hobbs FDR, et al. Screening for atrial fibrillation: a call for evidence. *European Heart Journal* 2019 doi: 10.1093/eurheartj/ehz834
22. Harris K, Edwards D, Mant J. How can we best detect atrial fibrillation? *The journal of the Royal College of Physicians of Edinburgh* 2012;42:5-22.
23. Welton NJ, McAleenan A, Thom HH, et al. Screening strategies for atrial fibrillation: a systematic review and cost-effectiveness analysis. *Health Technology Assessment* 2017;21(29)
24. Fitzmaurice DA, McCahon D, Baker J, et al. Is screening for AF worthwhile? Stroke risk in a screened population from the SAFE study. *Fam Pract* 2014;31(3):298-302. doi: 10.1093/fampra/cmu011 [published Online First: 2014/04/15]
25. Kim D, Yang PS, Yu HT, et al. Risk of dementia in stroke-free patients diagnosed with atrial fibrillation: data from a population-based cohort. *Eur Heart J* 2019;40(28):2313-23. doi: 10.1093/eurheartj/ehz386 [published Online First: 2019/06/19]
26. Hart RG, Pearce LA, Aguilar MI. Meta-analysis: antithrombotic therapy to prevent stroke in patients who have nonvalvular atrial fibrillation. *Ann Intern Med* 2007;146(12):857-67. doi: 10.7326/0003-4819-146-12-200706190-00007 [published Online First: 2007/06/20]
27. Martinez C, Katholing A, Freedman SB. Adverse prognosis of incidentally detected ambulatory atrial fibrillation. A cohort study. *Thromb Haemost* 2014;112(2):276-86. doi: 10.1160/th4-04-0383 [published Online First: 2014/06/24]
28. Lopez-Lopez JA, Sterne JAC, Thom HHZ, et al. Oral anticoagulants for prevention of stroke in atrial fibrillation: systematic review, network meta-analysis, and cost effectiveness analysis. *Bmj* 2017;359:j5058. doi: 10.1136/bmj.j5058 [published Online First: 2017/12/01]
29. Yiin GS, Howard DP, Paul NL, et al. Age-specific incidence, outcome, cost, and projected future burden of atrial fibrillation-related embolic vascular events: a population-based study. *Circulation* 2014;130(15):1236-44. doi: 10.1161/circulationaha.114.010942 [published Online First: 2014/09/12]
30. Romiti GF, Pastori D, Rivera-Caravaca JM, et al. Adherence to the 'Atrial Fibrillation Better Care' Pathway in Patients with Atrial Fibrillation: Impact on Clinical Outcomes—A Systematic Review and Meta-Analysis of 285,000 Patients. *Thromb Haemost* 2021(EFirst) [published Online First: 21.05.2021]
31. Hindricks G, Potpara T, Dagres N, et al. 2020 ESC Guidelines for the diagnosis and management of atrial fibrillation developed in collaboration with the European Association for Cardio-Thoracic Surgery (EACTS): The Task Force for the diagnosis and management of atrial fibrillation of the European Society of Cardiology (ESC) Developed with the special contribution of the European Heart Rhythm Association (EHRA) of the ESC. *European Heart Journal* 2020;42(5):373-498. doi: 10.1093/eurheartj/ehaa612
32. Friberg L, Rosenqvist M, Lindgren A, et al. High prevalence of atrial fibrillation among patients with ischemic stroke. *Stroke* 2014;45(9):2599-605. doi: 10.1161/strokeaha.114.006070 [published Online First: 2014/07/19]

For peer review only - http://bmjopen.bmj.com/site/about/guidelines.xhtml

33. King S, Fitzgerald A, Bartlett C, et al. Evidence Summary for Screening for Atrial Fibrillation in Adults: External review against programme appraisal criteria for the UK National Screening Committee: UK National Screening Committee, 2019.
34. Gomez-Outes A, Lagunar-Ruiz J, Terleira-Fernandez AI, et al. Causes of Death in Anticoagulated Patients With Atrial Fibrillation. *J Am Coll Cardiol* 2016;68(23):2508-21. doi: 10.1016/j.jacc.2016.09.944 [published Online First: 2016/12/10]
35. Pisters R, Lane DA, Nieuwlaat R, et al. A novel user-friendly score (HAS-BLED) to assess 1-year risk of major bleeding in patients with atrial fibrillation: the Euro Heart Survey. *Chest* 2010;138(5):1093-100.
36. Pritchett RV, Bem D, Turner GM, et al. Improving the Prescription of Oral Anticoagulants in Atrial Fibrillation: A Systematic Review. *Thromb Haemost* 2019;119(02):294-307.
37. Holt TA, Hunter TD, Gunnarsson C, et al. Risk of stroke and oral anticoagulant use in atrial fibrillation: a cross-sectional survey. *Br J Gen Pract* 2012;62(603):e710-e17.
38. Maura G, Billionnet C, Drouin J, et al. Oral anticoagulation therapy use in patients with atrial fibrillation after the introduction of non-vitamin K antagonist oral anticoagulants: findings from the French healthcare databases, 2011–2016. *BMJ Open* 2019;9(4):e026645. doi: 10.1136/bmjopen-2018-026645
39. Lund J, Saunders CL, Edwards D, et al. Anticoagulation trends in adults aged 65 years and over with atrial fibrillation: a cohort study. *Open Heart* 2021;8(2):e001737. doi: 10.1136/openhrt-2021-001737
40. Lown M, Moran P. Should we screen for atrial fibrillation? *BMJ* 2019;364:l43. doi: 10.1136/bmj.l43
41. Lowres N, Neubeck L, Redfern J, et al. Screening to identify unknown atrial fibrillation. A systematic review. *Thromb Haemost* 2013;110(2):213-22. doi: 10.1160/th13-02-0165 [published Online First: 2013/04/19]
42. Svennberg E, Engdahl J, Al-Khalili F, et al. Mass Screening for Untreated Atrial Fibrillation: The STROKESTOP Study. *Circulation* 2015;131(25):2176-84. doi: 10.1161/circulationaha.114.014343 [published Online First: 2015/04/26]
43. Wilson JMG, Jungner G, Organization WH. Principles and practice of screening for disease. 1968
44. Public Health England. Criteria for appraising the viability, effectiveness and appropriateness of a screening programme: GOV.UK; 2015 [updated 23/10/2015. Available from: https://www.gov.uk/government/publications/evidence-review-criteria-national-screening-programmes/criteria-for-appraising-the-viability-effectiveness-and-appropriateness-of-a-screening-programme accessed 24/03/2020.
45. US Preventive Services Task Force. Screening for Atrial Fibrillation: US Preventive Services Task Force Recommendation Statement. *Jama* 2022;327(4):360-67. doi: 10.1001/jama.2021.23732
46. Svennberg E, Friberg L, Frykman V, et al. Clinical outcomes in systematic screening for atrial fibrillation (STROKESTOP): a multicentre, parallel group, unmasked, randomised controlled trial. *The Lancet* 2021 doi: https://doi.org/10.1016/S0140-6736(21)01637-8
47. Svendsen JH, Diederichsen SZ, Højberg S, et al. Implantable loop recorder detection of atrial fibrillation to prevent stroke (The LOOP Study): a randomised controlled trial. *The Lancet* 2021 doi: https://doi.org/10.1016/S0140-6736(21)01698-6
48. Freedman B, Lowres N. High-intensity atrial fibrillation screening to prevent stroke. *The Lancet* 2021 doi: https://doi.org/10.1016/S0140-6736(21)01785-2
49. Lowres N, Freedman B. Population screening for atrial fibrillation to prevent stroke. *The Lancet* 2021 doi: https://doi.org/10.1016/S0140-6736(21)01750-5
50. SAFER team. The SAFER Study – Screening for Atrial Fibrillation with ECG to Reduce stroke: University of Cambridge; 2019 [Available from: https://www.safer.phpc.cam.ac.uk/ accessed 25/03/2020.

For peer review only - http://bmjopen.bmj.com/site/about/guidelines.xhtml

51. Engdahl J, Andersson L, Mirskaya M, et al. Stepwise Screening of Atrial Fibrillation in a 75-Year-Old Population. *Circulation* 2013;127(8):930-37. doi: doi:10.1161/CIRCULATIONAHA.112.126656
52. National Statistics: Ministry of Housing CLG. English indices of deprivation, 2019: gov.uk; 2019 [Available from: https://www.gov.uk/government/statistics/english-indices-of-deprivation-2019 accessed 11/04/2022.
53. Oxford University Innovation Ltd. Sortition - clinical trial software: University of Oxford Primary Care Clinical Trials Unit; 2022 [Available from: https://www.phctrials.ox.ac.uk/software/sortition accessed 06/05/2022.
54. Mant J, Burt J, Griffin S, et al. The SAFER study (Screening for Atrial Fibrillation with ECG to Reduce stroke): is it feasible to screen for atrial fibrillation in general practice? *International Journal of Stroke* 2020;15:551.
55. National Institute for Health and Care Excellence. Atrial Fibrillation: management: clinical guideline 180: National Institute for Health and Care Excellence, 2014.
56. Perez MV, Mahaffey KW, Hedlin H, et al. Large-Scale Assessment of a Smartwatch to Identify Atrial Fibrillation. *New England Journal of Medicine* 2019;381(20):1909-17. doi: 10.1056/NEJMoa1901183
57. Gladstone DJ, Wachter R, Schmalstieg-Bahr K, et al. Screening for Atrial Fibrillation in the Older Population: A Randomized Clinical Trial. *JAMA Cardiology* 2021 doi: 10.1001/jamacardio.2021.0038
58. Friberg L, Engdahl J, Frykman V, et al. Population screening of 75- and 76-year-old men and women for silent atrial fibrillation (STROKESTOP). *EP Europace* 2012;15(1):135-40. doi: 10.1093/europace/eus217
59. Svennberg E, Stridh M, Engdahl J, et al. Safe automatic one-lead electrocardiogram analysis in screening for atrial fibrillation. *EP Europace* 2016;19(9):1449-53. doi: 10.1093/europace/euw286
60. Hobbs FD, Fitzmaurice DA, Mant J, et al. A randomised controlled trial and cost-effectiveness study of systematic screening (targeted and total population screening) versus routine practice for the detection of atrial fibrillation in people aged 65 and over. The SAFE study. *Health technology assessment (Winchester, England)* 2005;9(40):iii-iv, ix-x, 1-74. doi: 10.3310/hta9400 [published Online First: 2005/10/06]
61. Marteau TM, Bekker H. The development of a six-item short-form of the state scale of the Spielberger State—Trait Anxiety Inventory (STAI). *British Journal of Clinical Psychology* 1992;31(3):301-06. doi: https://doi.org/10.1111/j.2044-8260.1992.tb00997.x
62. Buchholz I, Janssen MF, Kohlmann T, et al. A Systematic Review of Studies Comparing the Measurement Properties of the Three-Level and Five-Level Versions of the EQ-5D. *PharmacoEconomics* 2018;36(6):645-61. doi: 10.1007/s40273-018-0642-5
63. Ware JE, Kosinski M, Dewey JE, et al. How to score and interpret single-item health status measures: a manual for users of the SF-8 health survey. *Lincoln, RI: QualityMetric Incorporated* 2001;15(10):5.
64. Qualtrics. QualtricsXM 2022 [Available from: https://www.qualtrics.com/uk/ accessed 15/03/2022.
65. Campbell MK, Piaggio G, Elbourne DR, et al. Consort 2010 statement: extension to cluster randomised trials. *Bmj* 2012;345:e5661. doi: 10.1136/bmj.e5661 [published Online First: 2012/09/07]
66. Drummond MF, Sculpher MJ, Claxton K, et al. Methods for the Economic Evaluation of Health Care Programmes. Oxford: Oxford: Oxford University Press 2015.
67. Joint Formulary Committee. British National Formulary (online). London, UK: London: BMJ Group and Pharmaceutical Press, 2022.
68. Office for National Statistics. Dataset: Principal projection - UK population in age groups London, UK: Officer for National Statistics; 2022 [Available from:

For peer review only - http://bmjopen.bmj.com/site/about/guidelines.xhtml

https://www.ons.gov.uk/peoplepopulationandcommunity/populationandmigration/populationprojections/datasets/tablea21principalprojectionukpopulationinagegroups accessed 11/04/2022.

SUPPLEMENTARY MATERIALS

A – Trial invite letter

B – Trial PIS

C – Trial ICF

D – Screening Information Leaflet

For peer review only

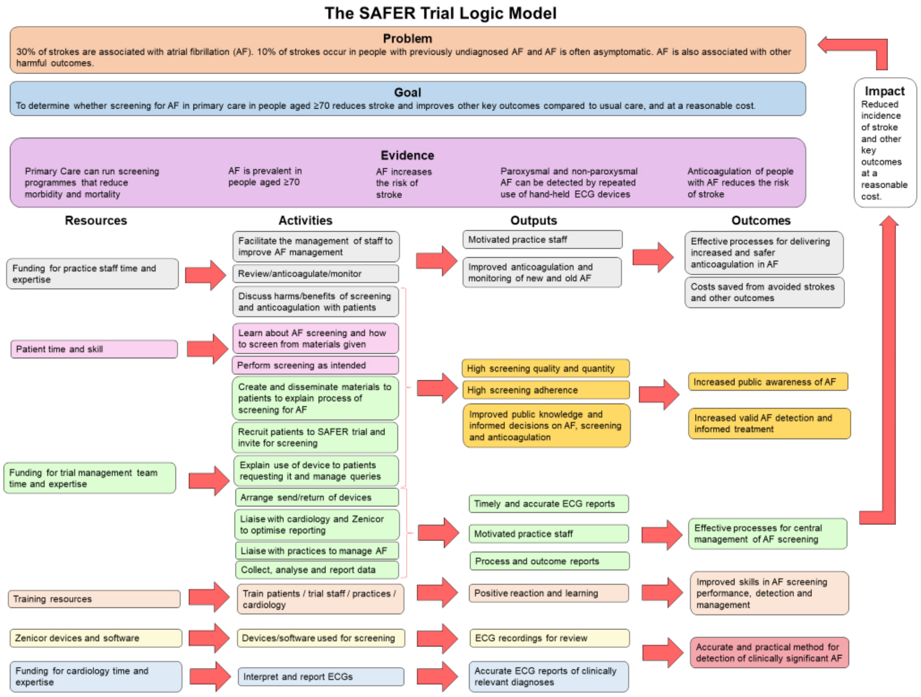

Figure 1. Logic model of the intervention in the SAFER trial

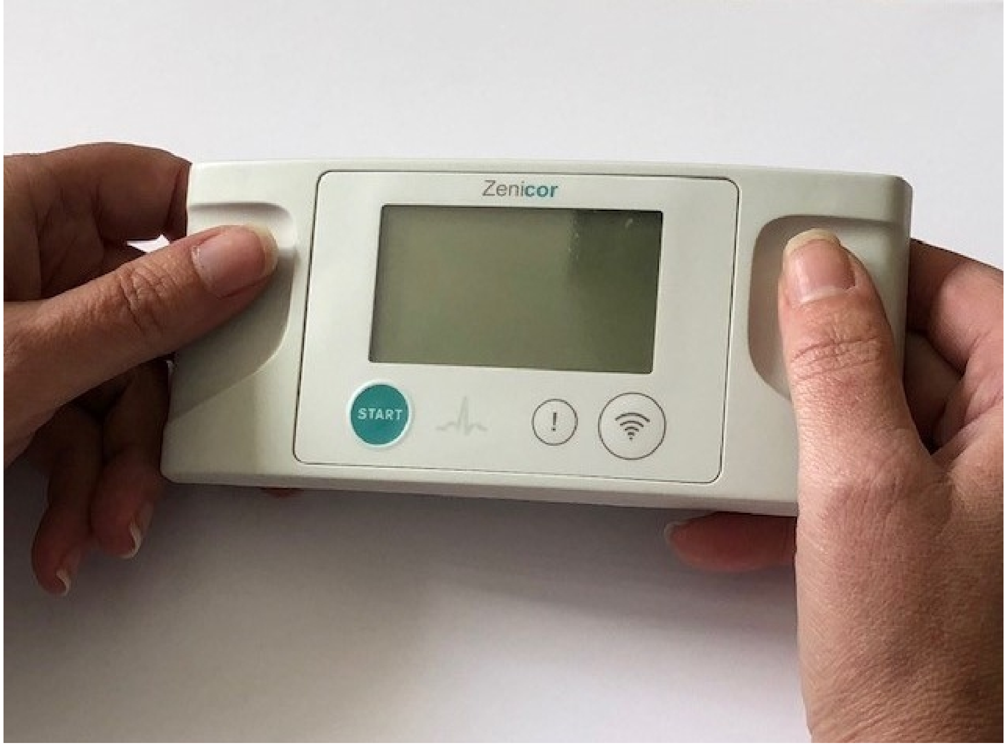

Figure 2. Zenicor hand-held ECG device used to screen for AF in the SAFER trial

225x167mm (72 x 72 DPI)

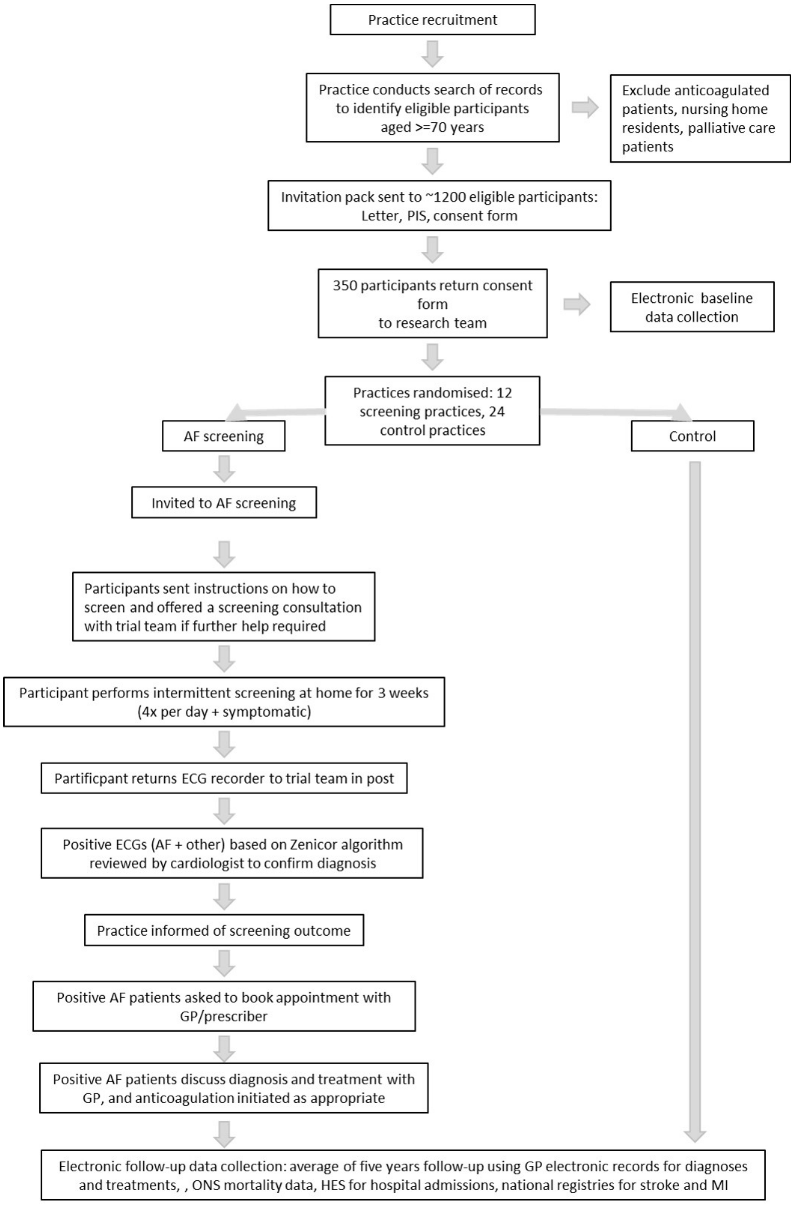

Figure 3. SAFER Trial schematic

178x271mm (150 x 150 DPI)

PRACTICE HEADED PAPER
PRACTICE ADDRESS
PRACTICE PARTNERS

Participant ID: <<Trial_ID>>

<<Title>> <<First name>> <<Surname>>
<<Address 1>>
<<Address 2>>
<<Address 3>>
<<Address 4>>
<<Address 5>>

<<Date>>

Dear <<Title>> <<First name>> <<Surname>>

For peer review only

Invitation to join a research trial:

The SAFER Trial

We are writing to let you know about a research trial that we are involved with. We are working with researchers from the University of Cambridge.

The trial aims to test whether screening at home for a common heart rate condition, Atrial Fibrillation (AF) would prevent people having strokes and heart attacks.

We are inviting you because you are in the age range where atrial fibrillation is more common. You have been selected at random from patients at your practice in this age range. You will not need to attend an appointment at the practice. You may still be able to take part if you already have atrial fibrillation.

You do not have to participate. Your decision will not affect any future health care you receive.

Enclosed with this letter is:

1. **Participant Information Sheet**
2. **Consent Form (including contact details) (complete and return to take part)**
3. **'I do not wish to take part' Reply Slip (ONLY complete and return if you DO NOT want to take part)**
4. **Freepost envelope**

Please read the **Participant Information Sheet,** the **Consent Form** and **Reply Slip** carefully. Please take your time to consider all the information. You can talk to others about your decision to take part, and contact the researchers to ask them any questions you have, or talk to your GP.

Once you have made your decision, please see instructions at the bottom of page 2 for what you need to do next. (**Note:** If the research team does not hear from you we may send you a **reminder letter**.)

For peer review only - http://bmjopen.bmj.com/site/about/guidelines.xhtml

PRACTICE HEADED PAPER
PRACTICE ADDRESS
PRACTICE PARTNERS

If you would like more information

If you have any questions please contact the SAFER Trial team by **phone** on **01223 763491** (working hours are Monday to Friday 9am-5pm, there is an answer phone on this number if they miss your call), or you can contact them by **email**, safer@medschl.cam.ac.uk. There is also more information about the trial on the website - https://www.safer.phpc.cam.ac.uk/.

Thank you for taking the time to read this letter.

Yours sincerely,

<Signature>

<Name of GP>

For peer review only

What to do next:

✓ I WOULD like to take part

If you would like to take part please complete the trial consent form. You can do this either:

Online: by following this secure link bit.ly/saferconsent and entering the code below to fill in your consent form online:

Code: <<Token>>

OR;

By post: by completing the enclosed **Consent Form**, checking that the contact details on the second page of the form are accurate. Please amend and add any if applicable. Please return the completed **Consent Form** to the research team in the Freepost envelope provided (no stamp required).

X I DO NOT want to take part

If you do not wish to take part, we would appreciate it if you would complete the **'I do not wish to take part'** Reply Slip enclosed and return it to the research team in the Freepost envelope provided (no stamp required). We would appreciate it if you would let the researchers know the reasons why you do not wish to participate in the SAFER trial.

For peer review only - http://bmjopen.bmj.com/site/about/guidelines.xhtml

UNIVERSITY OF CAMBRIDGE
Public Health and Primary Care
The Primary Care Unit

SAFER trial

The SAFER Trial –

Screening for Atrial Fibrillation with ECG to Reduce stroke

Participant Information Sheet

V1.3 03-02-2021_remote

We invite you to take part in a research trial about screening for atrial fibrillation to reduce stroke

- Before you decide whether to take part, it is important for you to understand why the research is being done and what it will involve.
- Please take your time to read the following information carefully. You can discuss it with your friends and relatives if you wish.
- You are free to decide whether or not to take part in this trial. If you choose not to take part, it will not affect the care that you receive from your doctors.
- Please don't hesitate to ask us any questions you may have, if anything is unclear, or if you would like more information.

FUNDED BY
NIHR | National Institute for Health Research
This project is funded by the National Institute for Health Research (NIHR) [Programme Grants for Applied Research (grant reference number RP-PG-0217-20007)]. The views expressed are those of the author(s) and not necessarily those of the NIHR or the Department of Health and Social Care.

For peer review only - http://bmjopen.bmj.com/site/about/guidelines.xhtml

Part 1: Trial Summary

A brief summary of the trial

- Atrial fibrillation (AF) is a common irregular heartbeat, and is a major risk factor for stroke unless treated with medication.

- We (researchers at the University of Cambridge and the University of Oxford) want to find out whether a national screening programme for atrial fibrillation (i) is possible/achievable, (ii) will prevent strokes and (iii) is good value for money for the NHS.

- We are inviting patients aged 70 years and over to take part in a research trial that aims to answer these questions. If you have atrial fibrillation you may still be able to take part.

- If you agree to take part, we will ask for your consent to give us access to relevant information from your medical and other health-related records. This will be information related to stroke and associated conditions (you can read more about this later in this information sheet). We will make sure that this information is looked after securely, remains confidential and is only used for the purposes described – all in accordance with the General Data Protection Regulations.

- Your practice may or may not offer screening for atrial fibrillation as part of the trial. If your practice does offer atrial fibrillation screening we will send you further information about this and you can choose then whether you would like to be screened or not.

- You can also visit the trial website at https://www.safer.phpc.cam.ac.uk

- If you have any questions you can phone us on **01223 763491** or email at safer@medschl.cam.ac.uk

Contents

Part 1
Trial Summary........*Page 2*

Part 2
Further Trial Details..........................*Page 3*

Part 3
Data Confidentiality............*Page 8*

Part 4
Contact Us................*Page 11*

How to contact us

If you have any questions you can call us on **01223 763491** (Monday to Friday 9am-5pm)

Or email us at safer@medschl.cam.ac.uk

You can also visit the trial website at www.safer.phpc.cam.ac.uk

For peer review only - http://bmjopen.bmj.com/site/about/guidelines.xhtml

Part 2: Further Trial Details

Why are we doing this trial?

Atrial fibrillation (AF) is a heart condition that causes an irregular heartbeat. It affects over 1 in 10 people over the age of 70 but does not necessarily cause symptoms.

Having atrial fibrillation increases the risk of having a stroke 5-fold, but treatment with medication can significantly lower this risk as well as lowering your risk of having a heart attack.

We want to find out whether screening people over the age of 70 years for atrial fibrillation and treating them with medication:

1. is possible/achievable in GP practices (**no GP practice visit needed**);
2. prevents the number of strokes we think it will;
3. is good value for money for the NHS.

The results will help the NHS decide whether to start a national screening programme for atrial fibrillation to reduce the number of strokes.

Why have I been invited?

You have been invited because you are in the age range where atrial fibrillation is more common. You have been selected, at random, from patients at your practice in this age range.

I have a heart condition. Can I take part?

Yes. If you have the following you can still take part:

- Atrial fibrillation and are **not** taking blood thinning (anticoagulation) medication like warfarin
- A pacemaker
- If you are taking aspirin or clopidogrel

Do I have to take part?

No. You do not have to take part. If you decide not to take part, or change your mind at any stage, your care at your GP practice will not be affected.

For peer review only - http://bmjopen.bmj.com/site/about/guidelines.xhtml

What will I have to do?

Consent

We will ask you to confirm your consent to take part. You can do this online by following the instructions in the covering letter. Alternatively, you can sign the enclosed paper consent form and post it back to us in the Freepost envelope provided.

We are asking you to give permission for specific information from your medical records to be shared with the research team at various points over the next few years. This information will be collected from your GP practice records and from other health-related records. The kind of information we will collect will include the medications you take, your use of health care services and stroke and cardiovascular disease related factors (for example weight, age, blood pressure, other medical conditions, and how well your kidneys and liver work). You will not need to do anything to provide this information. We will collect this information from everyone who consents to be part of the trial, whether they are screened for atrial fibrillation or not. Collecting this information will help us to understand whether screening for atrial fibrillation makes a difference, by comparing information about people who do and do not undergo this screening.

What else might I be invited to do?

Possibly take part in screening for atrial fibrillation

You may be invited to be screened for atrial fibrillation. This would be done at home. You would be sent the ECG recording device and instructions. There would be no need to attend an appointment at your GP practice or for anyone to visit you at home. Screening involves simply holding a small, safe, non-invasive recording device in your hands for 30 seconds at a time.

We will send you more information about what is involved and the potential benefits and harms of screening at the time if you are invited.

You are under no obligation to take part.

Not all the practices that are taking part in the trial will offer atrial fibrillation screening. Whether your practice does or not is determined by chance, so it may be that you are not invited to have screening.

For peer review only - http://bmjopen.bmj.com/site/about/guidelines.xhtml

Possibly complete questionnaires

On up to 3 separate occasions we may send you a questionnaire to complete and return in a Freepost envelope (or complete online). You are under no obligation to complete this questionnaire.

Possibly be invited to take part in other studies

Some people may be invited to take part in optional interviews and/or other studies related to atrial fibrillation and/or screening. If this is the case, you will be given further information about what these would involve. You will be free to decide at that point whether or not you want to do them.

Are there any benefits of taking part?

There may not be any direct benefit to you of taking part. However, you may find it rewarding to know that you are contributing to research that aims to prevent stroke and heart attacks in the future.

Are there any risks involved in taking part?

Identifiable medical data from your practice will be shared with the research team. All our data collection, storage and handling processes will comply with the relevant security policies and regulations. Every effort will be made to ensure the security and confidentiality of your data. Your usual medical care will not be affected by your participation in the trial.

What if I change my mind about giving access to medical and health-related records?

If you change your mind about taking part you can withdraw your consent at any time. If this is the case, please telephone us or email us using the contact details in part 4 of this information sheet. Any of your information that has been collected up to that point will be kept and used for the purposes described in this information sheet. We will not collect any further health-related information about you and your medical care at your GP practice will not be affected.

For peer review only - http://bmjopen.bmj.com/site/about/guidelines.xhtml

Who is organising and funding the trial?

The trial is being organised by the University of Cambridge working with the University of Oxford. The University of Cambridge and Cambridgeshire and Peterborough Clinical Commissioning Group (CCG) are the co-sponsors for the trial. The sponsors have overall responsibility for the conduct of the trial. It is funded by the National Institute for Health Research.

Who has reviewed / approved the trial?

To protect your interests, all research in the NHS is looked at by an independent group of people, called a Research Ethics Committee. This trial has been reviewed and given favourable opinion by the London-Central NHS Research Ethics Committee. The science has been reviewed by experts in atrial fibrillation, stroke and screening in the NHS.

How have patients and the public been involved in the trial?

Patient representatives and members of the public have been involved with the design of the trial and/or this information sheet. Patients are represented on a number of the trial research committees, and one is a co-investigator on the research grant.

What will happen if something goes wrong?

If you have any concerns about any aspect of this trial, you should ask to speak to us (the research team) and we will do our best to answer your questions:

Telephone: 01223 763491

Email: safer@medschl.cam.ac.uk

Post: The SAFER Trial, University of Cambridge, Primary Care Unit, Strangeways Research Laboratory, 2 Worts Causeway, Cambridge, CB1 8RN

If you remain unhappy and wish to complain formally, please first contact Cambridgeshire and Peterborough Clinical Commissioning Group patient experience team.

FREEPHONE: 0800 279 2535 or 01223 725 588

Email: CAPCCG.pet@nhs.net

Post: Patient Experience Team, Lockton House, Clarendon Road, Cambridge, CB2 8FH

For peer review only - http://bmjopen.bmj.com/site/about/guidelines.xhtml

Please note that due to the Covid-19 pandemic the patient experience team may not currently be able to respond to your query in a timely manner. Due to staff working remotely, contact by phone or email in the first instance is advised.

If you are unhappy with a primary care service, such as your GP practice or pharmacist, you can complain either directly to the Practice Manager of the practice or if you prefer to NHS England, the organisation which manages complaints for these services:

Telephone: 0300 311 22 33 (Monday to Friday 8am to 6pm, excluding bank holidays)

Email: england.contactus@nhs.net

Write to: NHS England, PO Box 16738, Redditch B97 9PT

In the event that something does go wrong and you are harmed during the research and this is due to someone's negligence then you may have grounds for legal action for compensation against the University of Cambridge or the NHS or an individual through their professional indemnity (if appropriate) but you may have to pay your legal costs. The normal National Health Service complaints mechanisms will still be available to you (if appropriate). The University of Cambridge has arranged insurance in case something goes wrong and you are harmed but it is not due to anybody's fault (no-fault compensation).

How will I find out the results of the trial?

At the end of this trial the results will be available to read on our trial website – please see our contact details in part 4 for the link. If you would like us to send you a copy of the results please get in touch with us.

For peer review only - http://bmjopen.bmj.com/site/about/guidelines.xhtml

Part 3: Data Confidentiality

How will information about me be kept confidential?

Your personal details will be collected from your GP practice including your name, address, contact details, date of birth, NHS number – “personal data”. We will also collect information about you during your participation in the trial, some relating to your health from various sources – “trial data”.

Your trial data will not include your personal data, and will be stored separately using a unique trial identification number.

All information about you (including your personal data) will be stored securely with access restricted to authorised members of the research team from the University of Cambridge and the University of Oxford. Only these people will access your personal data as they need to manage your participation in the trial, collect information from your medical and health records, or audit the data collection process. In addition, authorised staff who work for or with the sponsors of the trial or relevant regulatory authorities may require access to your personal data, your trial data and/or your medical records. This would be to check the accuracy of the trial data and ensure that it is being conducted in accordance with the relevant regulations. All information will be treated in the strictest confidence during that review process.

We will inform your GP that you are taking part in the trial.

Information from medical and health records

The research team will collect information from your GP practice medical record. Usually your practice will pass this information securely to the research team. Sometimes your GP practice will allow the research team to access your medical records directly, or use a contracted third party. This is only done with the appropriate security checks and confidentiality agreements in place.

The research team will also collect information from other centrally held health-related records. The records we will use are Hospital Episodes Statistics data, civil registration mortality data (both held by the appropriate governing body, currently NHS Digital), the Sentinel Stroke National Audit Programme (SSNAP) database, and the Myocardial Ischaemia National Audit Project (MINAP)

For peer review only - http://bmjopen.bmj.com/site/about/guidelines.xhtml

database. To link this information the research team need to send identifying information such as your name, address, NHS number and date of birth to NHS Digital, SSNAP and MINAP so they can identify your health records correctly. It is possible that in the future we may need to link to another health record or registry that we consider to be relevant to the purposes of the research. We will use central NHS records to provide us with your current GP practice and your address, so that we can continue to collect follow-up information should you move. We will continue to collect health record data in the event of your death during the trial and follow-up period.

Only information relevant to the purpose of the trial or understanding how to screen for atrial fibrillation will be collected. We might use this information to decide whether to invite you to take part in other related research studies.

General Data Protection Regulation

The University of Cambridge and NHS Cambridgeshire and Peterborough CCG are the co-sponsors for this trial based in the UK. The University of Cambridge will be using information from you and your medical records in order to undertake this trial and will act as the data controller for this trial. This means that we are responsible for looking after your information and using it properly. The SAFER Trial is part of an important long-term programme of research that relies on long-term follow-up of participants. We will retain your personal and trial data indefinitely to meet the purposes of medical research and any legal, accounting or reporting requirements.

Your rights to access, change or move the information that we hold are limited, as we need to manage it in specific ways in order for the research to be reliable and accurate. To safeguard your rights, we will use the minimum personally identifiable information possible.

You can find out more about how we use your information here https://www.medschl.cam.ac.uk/research/privacy-notice-how-we-use-your-research-data/

We will share your information with collaborating research organisations working with us (both internal and external to this trial) and commercial partners. Only trial data that cannot identify you will be shared. This will be governed by appropriate agreements. Some organisations may be outside of the European Economic Area, where data security regulations may be less stringent than those in the UK. The transfer of information will be done securely and in accordance with local security policies and the Data Protection Act 2018.

For peer review only - http://bmjopen.bmj.com/site/about/guidelines.xhtml

During the trial we will not be able to monitor your ability to consent to continued participation. We will keep any data already collected about you and will continue to collect data from your medical and other health records, in accordance with the consent that you granted at the start of the trial. If, however, we find out as a result of arranging your screening or through your involvement in optional interviews that you are unwell such that you are no longer able to consent to continued participation, we will withdraw you from the trial, while keeping any data about you that we have already collected.

Future updates relating to data confidentiality will be posted on the trial website https://www.safer.phpc.cam.ac.uk/. It is recommended that you visit the website regularly to keep up to date with the progress of the trial and data confidentiality information.

For peer review only

For peer review only - http://bmjopen.bmj.com/site/about/guidelines.xhtml

Part 4: Contact Us

Who do I contact if I have any questions?

Please get in touch with us - our details are below. You can also visit the trial website: https://www.safer.phpc.cam.ac.uk/ for more information.

The trial is registered with the ISRCTN https://www.isrctn.com/ISRCTN72104369.

For peer review only

Contact details:

If you or someone on your behalf needs to contact the research team you can do so as follows:

Phone: contact us using the following number during working hours (Monday to Friday 9am – 5pm): **01223 763491**. If we miss your call or if you call outside these hours, there is an answer phone on this number. If you leave a message we will respond to you at the earliest opportunity.

Email: safer@medschl.cam.ac.uk

Address:
The SAFER Trial
University of Cambridge
Primary Care Unit
Strangeways Research Laboratory
2 Worts Causeway
Cambridge
CB1 8RN

Website: https://www.safer.phpc.cam.ac.uk/

For peer review only - http://bmjopen.bmj.com/site/about/guidelines.xhtml

For peer review only

SAFER trial

For peer review only - http://bmjopen.bmj.com/site/about/guidelines.xhtml

[TO BE PRINTED ON TRIAL HEADED PAPER]

Participant ID: <<participant ID>> / barcode

SAFER Trial

Consent Form

Version 1.1 11-12-2020

Please complete and return this form only if you wish to join the SAFER Trial

Title: The SAFER Trial – Screening for Atrial Fibrillation with ECG to Reduce stroke

Chief Investigator: Professor Jonathan Mant, University of Cambridge

IRAS project ID: 272184

Ethics Reference number: 19/LO/1597 **Participant ID**: <<participant ID>> / barcode

If you are willing to take part in the SAFER Trial, please read the following statements and if you agree, sign and date overleaf.

1	I have read and understood the Participant Information Sheet version XX, dated DATE (NAME) for the above trial. I have had the opportunity to ask questions and I am satisfied with the answers and explanations provided.
2	I understand that my participation in this trial is voluntary and that I am free to withdraw at any time, without giving a reason and without my medical care or legal rights being affected.
3	I understand that information from my medical records will be available to the research team as part of the trial.
4	I consent to my trial data being linked to Hospital Episodes Statistics (HES), civil registration mortality data, Sentinel Stroke National Audit Programme (SSNAP) and Myocardial Ischaemia National Audit Project (MINAP). This may involve sharing my personal data with these bodies. I understand that information held and managed by NHS Digital and the registries may be used in order to provide information about my health status (including after my death), my GP practice and my address (should I move). I understand that these details will be used for research purposes only. It is possible that in the future the research team may need to link to another health record or registry not listed that they consider to be relevant to the purposes of the research and I agree to this.
5	I understand that sections of my medical notes or information related directly to my participation in this trial may be looked at by responsible individuals from the sponsors, regulatory authorities and research personnel where it is relevant to my taking part in this research. I give permission for these individuals to have access to my records.
6	I understand that my GP will be informed of my participation in this trial.
7	I understand that my unidentifiable trial data will be shared with other researchers, both internal and external to this trial, and with commercial partners. These parties may be outside the European Economic Area.
8	I understand that I may be contacted about future, related research studies, and that I am under no obligation to take part.
9	I agree to participate in this trial.

For peer review only - http://bmjopen.bmj.com/site/about/guidelines.xhtml

[TO BE PRINTED ON TRIAL HEADED PAPER]

Participant ID: <<participant ID>> / barcode

By signing this form you are consenting that you agree with all of the statements listed, and that the details listed below are correct.

Name of participant	Signature	Date

Please check the details below and amend/complete accordingly, then return this form to the trial team using the Freepost envelope enclosed. Alternatively you can complete this consent form online – please see the covering letter enclosed for instructions. As the trial will be conducted remotely, it will be helpful if you could please supply a phone number. If you also have an email address and are happy to provide this, please do so.

Title:	
First name:	
Surname:	
Date of birth (dd/mm/yyyy):	
Gender (M/F/Mx):	
Address:	
Postcode:	
Home Tel.:	
Mobile no.:	
Email:	
NHS no:	
GP Practice name:<br>**Please note:** if this is **not** your current practice and you have recently moved practice, you will not be able to take part at this point. It is possible that your new practice may take part in the future.	

The trial team will return a copy of this consent form to your GP practice for their records. If you would like a copy of your completed consent form please contact the trial team.

The trial team will only use these details in order to contact you for the purposes stated.

1x copy to be retained by the research team; 1x copy to be sent to the participant's GP practice.

For peer review only

For peer review only - http://bmjopen.bmj.com/site/about/guidelines.xhtml

UNIVERSITY OF CAMBRIDGE
Public Health and Primary Care
The Primary Care Unit

SAFER trial

Invitation to take part in screening for atrial fibrillation to reduce your risk of stroke

Screening Information Leaflet

V1.2 11-12-2020

For peer review only

FUNDED BY
NIHR | National Institute for Health Research

This project is funded by the National Institute for Health Research (NIHR) [Programme Grants for Applied Research (grant reference number RP-PG-0217-20007)]. The views expressed are those of the author(s) and not necessarily those of the NIHR or the Department of Health and Social Care.

For peer review only - http://bmjopen.bmj.com/site/about/guidelines.xhtml

Summary

This leaflet provides information on atrial fibrillation screening (also called AF screening) for people aged 70 and over who are already taking part in the SAFER research trial.

It explains what atrial fibrillation (AF) is and what happens when you take part in screening. It is important that you make a decision whether to take up this offer of screening based on all the available information.

Please read the information contained in this leaflet carefully. It will help you make this decision. If there is anything that you do not understand or have questions about, there is information at the end for where to go to ask any questions you may have. Screening is your choice.

For peer review only

For peer review only - http://bmjopen.bmj.com/site/about/guidelines.xhtml

Atrial fibrillation

Atrial fibrillation is a heart condition that causes an irregular and often abnormally fast heart rate. In some people this can cause symptoms like:

- palpitations
- breathlessness
- dizziness (feeling faint or light-headed),

but it may not cause any symptoms at all. Some people have atrial fibrillation all the time, for others it comes and goes. This can make it difficult to detect. About 3 in 100 (3%) people aged over 70 have atrial fibrillation without knowing it.

For peer review only

Risks of developing atrial fibrillation

Atrial fibrillation can affect adults of any age, but it is increasingly common as people grow older.

Screening for atrial fibrillation

Screening aims to identify atrial fibrillation, which can lead to strokes, heart failure, heart attacks and premature death. About 30% of strokes occur in people who have atrial fibrillation. Treating someone with atrial fibrillation can prevent this happening. It is also possible that atrial fibrillation may cause dementia and that management may reduce the likelihood of developing dementia.

For peer review only - http://bmjopen.bmj.com/site/about/guidelines.xhtml

Screening invitation

We are inviting you because your practice told us that you are over the age of 70 and are not on long term blood thinning (anticoagulant) medication, and you gave permission for us to include you in the SAFER research trial.

If you think you already have atrial fibrillation but do not take blood thinning (anticoagulant) medication you can still choose to be screened.

You can still take part if you take aspirin or clopidogrel. These are anti-platelet medications not anticoagulants – they thin the blood in a different way.

It is up to you to decide whether to be screened, you do not have to. Screening is your choice. If you decide not to be screened, your care at your GP practice will not be affected.

For peer review only - http://bmjopen.bmj.com/site/about/guidelines.xhtml

Screening test – 3 weeks screening at home

Screening is done using a simple, handheld device (called an electrocardiogram or 'ECG' recorder) that records your heart rhythm. The device is painless, safe, and easy to use. You simply place your thumbs on it for 30 seconds.

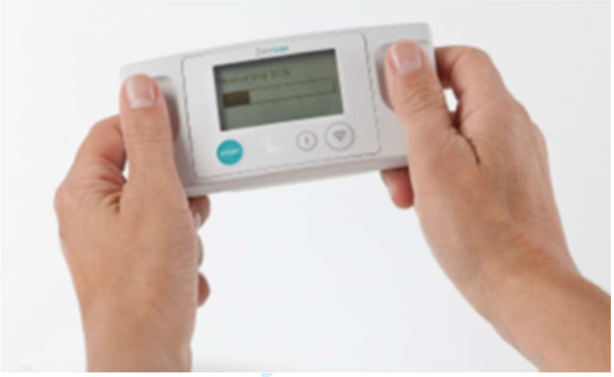

You will be contacted by the research team to check that it is convenient to send you the device. This will come with full instructions for how to use it. The device will be thoroughly cleaned before sending.

If you would like a second call from the research team after the device has arrived, to talk you through the instructions, just let them know this when they contact you about sending you the device.

You will be asked to keep the device at home for 3 weeks and use it 4 times a day and if you feel that your heart is beating irregularly.

For peer review only - http://bmjopen.bmj.com/site/about/guidelines.xhtml

There is a short video on the study website showing the ECG device being used https://vimeo.com/358042715. You may find it helpful to watch this although this is not essential as you will be sent full instructions for how to use it.

You are also welcome to contact the research team by phone during working hours (Monday to Friday 9am – 5pm) on **01223 763491** or by email **safer@medschl.cam.ac.uk**) if you have any questions about how to use the device.

The device stores the ECGs and transmits them over the mobile network. You do not need to have WiFi or a mobile phone to use the device.

At the end of the screening period you will need to return the device using the Freepost envelope that will be provided.

Next steps

The ECG traces will be analysed once the device is received back from you. The results will usually be available within 12 weeks from this date. If you have not heard by this time you may wish to contact the practice directly. If you move house, or GP practice, during this time please let the research team know.

For peer review only - http://bmjopen.bmj.com/site/about/guidelines.xhtml

It is very important that if at any point you have symptoms you seek medical help in the same way that you usually would, for example by calling 999, or contacting your GP. **Do not wait for the results of your screening test.**

Screening results

Your practice will inform you of your screening result and whether any action is required.

If you are found to have atrial fibrillation

If you are found to have atrial fibrillation, you will be invited to attend an appointment with your GP to discuss whether you need to start taking standard blood thinning (anticoagulant) medication. Your GP will help you make a decision about whether to start treatment, usually in the form of tablets.

It is likely that you will need to take this for the rest of your life. Your GP practice will arrange for appropriate monitoring of your medication.

Other health problems found by the screening

There is a very small chance that another abnormal rhythm will be detected. In this instance if any action is necessary your GP practice will contact you.

For peer review only - http://bmjopen.bmj.com/site/about/guidelines.xhtml

Potential benefits of being screened

If you are screened and found to have atrial fibrillation, you will be offered treatment which will greatly reduce your risk of having a stroke or heart attack and possibly dementia.

If another heart rhythm abnormality that is important to your health is diagnosed, you will be referred for further tests and / or treatment as necessary.

Potential harms of being screened

If you are found to have atrial fibrillation, and are started on treatment, this may increase your risk of bleeding, as your blood will not clot as well. This might include bleeding in the brain or the gut. You will have an opportunity when discussing treatment with your GP to weigh this risk up against the benefits of treatment.

Going through a screening process, like having any medical test, can cause anxiety in some people. You can speak to your nurse or doctor or the research team if you are anxious about screening. They can talk through any questions or concerns you have.

For peer review only - http://bmjopen.bmj.com/site/about/guidelines.xhtml

Being diagnosed with atrial fibrillation or another heart rhythm abnormality may affect any current or future insurance policies.

Reliability of the screening process

All the positive traces are reviewed by a cardiologist (heart specialist doctor), so the chance of an incorrect diagnosis of atrial fibrillation is very low. However, if you have the type of atrial fibrillation that comes and goes, it is possible that may not be detected if the ECG recording occurs at a point where atrial fibrillation is not present.

Storage of ECGs at the end of screening

They will be held on a secure database. If any of your ECGs show atrial fibrillation or another important abnormality they will also be stored by your practice. For more information about how we protect your data please refer to the participant information sheet sent by your practice at the start of the study. This is also available on the study website.

For peer review only

For peer review only - http://bmjopen.bmj.com/site/about/guidelines.xhtml

More information

If you have any questions about atrial fibrillation please visit the NHS Choices website https://www.nhs.uk/conditions/atrial-fibrillation

Alternatively you can speak to your GP.

If you have any questions about the SAFER Trial please visit the Trial website https://www.safer.phpc.cam.ac.uk/ or contact the research team.

Phone: contact us during working hours (Monday to Friday 9am – 5pm) on **01223 763491**. If you leave a message, we will respond to you at the earliest opportunity.

Email: safer@medschl.cam.ac.uk

Address:
The SAFER Trial
University of Cambridge
Primary Care Unit
Strangeways Research Laboratory
2 Worts Causeway
Cambridge
CB1 8RN

For peer review only

For peer review only - http://bmjopen.bmj.com/site/about/guidelines.xhtml

For peer review only

If you would like to take part in screening for atrial fibrillation, please see the accompanying letter for details of what you need to do next.

For peer review only - http://bmjopen.bmj.com/site/about/guidelines.xhtml

SAFER
trial

For peer review only

For peer review only - http://bmjopen.bmj.com/site/about/guidelines.xhtml

STANDARD PROTOCOL ITEMS: RECOMMENDATIONS FOR INTERVENTIONAL TRIALS

SPIRIT 2013 Checklist: Recommended items to address in a clinical trial protocol and related documents*

Section/item	Item No.	Description	Page
**Administrative information**			
Title	1	Descriptive title identifying the study design, population, interventions, and, if applicable, trial acronym	1
Trial registration	2a	Trial identifier and registry name. If not yet registered, name of intended registry	4
	2b	All items from the World Health Organization Trial Registration Data Set	Throughout
Protocol version	3	Date and version identifier	N/A
Funding	4	Sources and types of financial, material, and other support	19-20
Roles and responsibilitie s	5a	Names, affiliations, and roles of protocol contributors	1-3
	5b	Name and contact information for the trial sponsor	18
	5c	Role of study sponsor and funders, if any, in study design; collection, management, analysis, and interpretation of data; writing of the report; and the decision to submit the report for publication, including whether they will have ultimate authority over any of these activities	20
	5d	Composition, roles, and responsibilities of the coordinating centre, steering committee, endpoint adjudication committee, data management team, and other individuals or groups overseeing the trial, if applicable (see Item 21a for data monitoring committee)	18
**Introduction**			
Background and rationale	6a	Description of research question and justification for undertaking the trial, including summary of relevant studies (published and unpublished) examining benefits and harms for each intervention	6-7
	6b	Explanation for choice of comparators	8

For peer review only

Objectives	7	Specific objectives or hypotheses	8
Trial design	8	Description of trial design including type of trial (eg, parallel group, crossover, factorial, single group), allocation ratio, and framework (eg, superiority, equivalence, noninferiority, exploratory)	8
**Methods: Participants, interventions, and outcomes**			
Study setting	9	Description of study settings (eg, community clinic, academic hospital) and list of countries where data will be collected. Reference to where list of study sites can be obtained	8-9
Eligibility criteria	10	Inclusion and exclusion criteria for participants. If applicable, eligibility criteria for study centres and individuals who will perform the interventions (eg, surgeons, psychotherapists)	8-9
Interventions	11a	Interventions for each group with sufficient detail to allow replication, including how and when they will be administered	10-12
	11b	Criteria for discontinuing or modifying allocated interventions for a given trial participant (eg, drug dose change in response to harms, participant request, or improving/worsening disease)	8
	11c	Strategies to improve adherence to intervention protocols, and any procedures for monitoring adherence (eg, drug tablet return, laboratory tests)	9-11
	11d	Relevant concomitant care and interventions that are permitted or prohibited during the trial	8
Outcomes	12	Primary, secondary, and other outcomes, including the specific measurement variable (eg, systolic blood pressure), analysis metric (eg, change from baseline, final value, time to event), method of aggregation (eg, median, proportion), and time point for each outcome. Explanation of the clinical relevance of chosen efficacy and harm outcomes is strongly recommended	6-7, 12-16
Participant timeline	13	Time schedule of enrolment, interventions (including any run-ins and washouts), assessments, and visits for participants. A schematic diagram is highly recommended (see Figure)	11-12, 14-16, fig 3

For peer review only - http://bmjopen.bmj.com/site/about/guidelines.xhtml

Sample size	14	Estimated number of participants needed to achieve study objectives and how it was determined, including clinical and statistical assumptions supporting any sample size calculations	13-14
Recruitment	15	Strategies for achieving adequate participant enrolment to reach target sample size	9-10
**Methods: Assignment of interventions (for controlled trials)**			
Allocation:			
Sequence generation	16a	Method of generating the allocation sequence (eg, computer-generated random numbers), and list of any factors for stratification. To reduce predictability of a random sequence, details of any planned restriction (eg, blocking) should be provided in a separate document that is unavailable to those who enrol participants or assign interventions	9-10, 15-16
Allocation concealment mechanism	16b	Mechanism of implementing the allocation sequence (eg, central telephone; sequentially numbered, opaque, sealed envelopes), describing any steps to conceal the sequence until interventions are assigned	9-10, 15-16
Implementation	16c	Who will generate the allocation sequence, who will enrol participants, and who will assign participants to interventions	9-10, 15-16
Blinding (masking)	17a	Who will be blinded after assignment to interventions (eg, trial participants, care providers, outcome assessors, data analysts), and how	9-10, 15-16
	17b	If blinded, circumstances under which unblinding is permissible, and procedure for revealing a participant's allocated intervention during the trial	N/A
**Methods: Data collection, management, and analysis**			
Data collection methods	18a	Plans for assessment and collection of outcome, baseline, and other trial data, including any related processes to promote data quality (eg, duplicate measurements, training of assessors) and a description of study instruments (eg, questionnaires, laboratory tests) along with their reliability and validity, if known. Reference to where data collection forms can be found, if not in the protocol	14-16

	18b	Plans to promote participant retention and complete follow-up, including list of any outcome data to be collected for participants who discontinue or deviate from intervention protocols	11-12, 14-16
Data management	19	Plans for data entry, coding, security, and storage, including any related processes to promote data quality (eg, double data entry; range checks for data values). Reference to where details of data management procedures can be found, if not in the protocol	16
Statistical methods	20a	Statistical methods for analysing primary and secondary outcomes. Reference to where other details of the statistical analysis plan can be found, if not in the protocol	16
	20b	Methods for any additional analyses (eg, subgroup and adjusted analyses)	16
	20c	Definition of analysis population relating to protocol non-adherence (eg, as randomised analysis), and any statistical methods to handle missing data (eg, multiple imputation)	16
**Methods: Monitoring**			
Data monitoring	21a	Composition of data monitoring committee (DMC); summary of its role and reporting structure; statement of whether it is independent from the sponsor and competing interests; and reference to where further details about its charter can be found, if not in the protocol. Alternatively, an explanation of why a DMC is not needed	18
	21b	Description of any interim analyses and stopping guidelines, including who will have access to these interim results and make the final decision to terminate the trial	18
Harms	22	Plans for collecting, assessing, reporting, and managing solicited and spontaneously reported adverse events and other unintended effects of trial interventions or trial conduct	15-16, 18
Auditing	23	Frequency and procedures for auditing trial conduct, if any, and whether the process will be independent from investigators and the sponsor	18
**Ethics and dissemination**			

For peer review only - http://bmjopen.bmj.com/site/about/guidelines.xhtml

Research ethics approval	24	Plans for seeking research ethics committee/institutional review board (REC/IRB) approval	4,18
Protocol amendments	25	Plans for communicating important protocol modifications (eg, changes to eligibility criteria, outcomes, analyses) to relevant parties (eg, investigators, REC/IRBs, trial participants, trial registries, journals, regulators)	18
Consent or assent	26a	Who will obtain informed consent or assent from potential trial participants or authorised surrogates, and how (see Item 32)	9,19
	26b	Additional consent provisions for collection and use of participant data and biological specimens in ancillary studies, if applicable	N/A
Confidentialit y	27	How personal information about potential and enrolled participants will be collected, shared, and maintained in order to protect confidentiality before, during, and after the trial	16
Declaration of interests	28	Financial and other competing interests for principal investigators for the overall trial and each study site	19-20
Access to data	29	Statement of who will have access to the final trial dataset, and disclosure of contractual agreements that limit such access for investigators	16, 19
Ancillary and post-trial care	30	Provisions, if any, for ancillary and post-trial care, and for compensation to those who suffer harm from trial participation	N/A
Disseminatio n policy	31a	Plans for investigators and sponsor to communicate trial results to participants, healthcare professionals, the public, and other relevant groups (eg, via publication, reporting in results databases, or other data sharing arrangements), including any publication restrictions	19
	31b	Authorship eligibility guidelines and any intended use of professional writers	N/A
	31c	Plans, if any, for granting public access to the full protocol, participant-level dataset, and statistical code	19
**Appendices**			
Informed consent materials	32	Model consent form and other related documentation given to participants and authorised surrogates	Appendices A, B, C

For peer review only

Biological specimens	33	Plans for collection, laboratory evaluation, and storage of biological specimens for genetic or molecular analysis in the current trial and for future use in ancillary studies, if applicable	N/A

*It is strongly recommended that this checklist be read in conjunction with the SPIRIT 2013 Explanation & Elaboration for important clarification on the items. Amendments to the protocol should be tracked and dated. The SPIRIT checklist is copyrighted by the SPIRIT Group under the Creative Commons "Attribution-NonCommercial-NoDerivs 3.0 Unported" license.

For peer review only

For peer review only - http://bmjopen.bmj.com/site/about/guidelines.xhtml
